# Dynamic Evolution of the Tumor Immune Microenvironment in Malignant Tumors and Emerging Therapeutic Paradigms

**DOI:** 10.1002/mco2.70496

**Published:** 2025-12-03

**Authors:** Ying Sun, Changjian Shao, Hongtao Duan, Zhaoyang Wang, Shaopeng Xu, Chao Wang, Jiawei Xiu, Jin Liu, Xuejiao Wang, Xin Yao, Yuan Gao, Xiaolong Yan

**Affiliations:** ^1^ Department of Thoracic Surgery The Second Affiliated Hospital Air Force Medical University Xi'an China; ^2^ A State Key Laboratory of Cancer Biology Biotechnology Center School of Pharmacy Air Force Medical University Xi'an China

**Keywords:** cancer, clinical treatment strategies, dynamic evolution, tumor immune microenvironment

## Abstract

Cancer is more than just a collection of tumor cells. The complex tumor system, including the tumor immune microenvironment (TIME), is continually changing. Tumor cells are in constant communication with all stromal elements (e.g., fibroblasts, endothelial cells, and extracellular matrix) and immune effector cells (e.g., T cells, B cells, natural killer cells, dendritic cells, macrophages, and myeloid‐derived suppressor cells). Together, these intricate interactions among cell and molecular signaling pathways collectively drive tumor growth, tumor invasion, and metastasis and significantly affect the efficacy of cancer treatments. Recent investigations, from a tumor‐centric research paradigm to a complete evaluation of the local tumor microenvironment, have revealed the importance of the TIME. Although reviews in these fields typically focus on cellular/molecular breakdowns of the TIME and evasion of the immune system, a systematic study of its dynamic evolution is lacking. This review comprehensively discusses the major regulators and networks involved in the dynamic evolution of the TIME, the spatiotemporal dynamics of TIME components, metabolic reprogramming as an engine of TIME evolution, the targeting of metabolic regulators, and niches for TIME modulation, clinical and translational challenges, and future prospects. This information could help researchers explore the TIME and generate new therapeutic strategies.

## Graphical abstract

The current review comprehensively discusses the major regulators and networks involved in the dynamic evolution of the TIME, the spatiotemporal dynamics of TIME components, metabolic reprogramming as an engine of TIME evolution, targeting of metabolic regulators, and niches for TIME modulation, clinical and translational challenges, and future prospects. Through the discussion of these aspects, we hope to comprehensively summarize the entire process of the evolution of the TIME system.

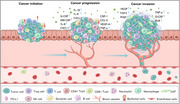

## 1 Introduction

As a complex disease, cancer has undergone profound changes in its research paradigm. Over the past few decades, research has focused mainly on tumor cells themselves, with the belief that their intrinsic genetic mutations (such as the activation of oncogenes and the inactivation of tumor suppressor genes) are the sole “engine” driving malignant transformation [1, 2, 3, 4, 5]. This “tumor‐centered” model has led to the development of numerous targeted therapeutic drugs and has achieved remarkable success [6, 7, 8, 9, 10]. However, this “autonomous nature of tumor cells” perspective cannot explain all the clinical phenomena. For instance, some healthy individuals carry cancer‐causing gene mutations but do not develop tumors [11, 12], whereas some patients develop metastatic lesions many years after the primary tumor has been removed [13]. More importantly, epidemiological data provide a more compelling answer: The carcinogenic driving factors alone can explain only a small portion (5–10%) of the cancer risk, while the vast majority (90–95%) of malignant tumors stem from our long‐term interactions with the environment (such as infections, pollutants, and psychological stress) and lifestyle (such as diet, smoking, and drinking) [14]. These features strongly suggest that the occurrence and development of tumors is a systemic disease that is jointly determined by the “seeds” (mutated cells) and the “soil” (the internal microenvironment).

For a long time, research on “soil,” the tumor microenvironment (TME), has been hindered by technical limitations. Traditional batch sequencing technology can obtain only the average signal of the cell population, thereby masking the significant heterogeneity within it. Moreover, high‐throughput molecular analysis of tissue sections, although capable of preserving spatial information, is difficult. In recent years, breakthroughs in single‐cell RNA sequencing (scRNA‐seq) [15, 16, 17, 18] and spatial transcriptomics [19, 20, 21] have finally enabled us to develop a “microscope” and a “satellite map,” allowing us to observe the gene expression status of every cell in the TIME and its precise location within the tissue with unprecedented resolution. Through these techniques, we have come to understand that a tumor is not merely a collection of cancer cells but rather a highly complex and constantly evolving “ecosystem” or “mini‐organ.” In this community, known as TIME, in addition to the “core residents”—the tumor cells—there are also various resident “neighbors,” including the stromal cells that build the infrastructure (such as fibroblasts and endothelial cells [ECs]), the immune cells that maintain order and provide defense (such as T cells, B cells, NK cells, macrophages, and dendritic cells [DCs]), and some myeloid‐derived suppressive cells (MDSCs) that have been “bought off” or “rebelled against,” among others. These residents are not isolated from each other [22]. They interact with one another through complex “crosstalk” (including various signaling molecules such as cytokines, exosomes, and various RNAs), jointly shaping the fate of the entire community—either to inhibit tumor growth or to promote its angiogenesis, immune evasion, and treatment resistance—and a series of other oncogenic phenotypes [23].

For instance, in the TIME, regulatory T cells (Tregs) and MDSCs can directly inhibit the functions of effector T cells through the secretion of cytokines such as IL‐10 and TGF‐β [24]. The upregulation of PD‐L1, an immune checkpoint molecule on the surface of tumor cells, can also induce T cell exhaustion [25]. Apart from the changes in immune cells such as T cells, the extracellular matrix (ECM) in TIME also plays a physical barrier role, preventing the infiltration of immune cells. And cancer‐associated fibroblasts (CAFs) can further remodel the ECM, exacerbating this physical barrier [26]. In addition, the hypoxic and acidic microenvironment in TIME also prompts macrophages to polarize toward the immunosuppressive M2 type [27]. These factors jointly create a complex immunosuppressive microenvironment.

In recent years, with the transformation of tumor treatment methods, immunotherapy strategies and related research have been continuously deepening. It has been discovered that TIME not only has complex metabolic interactions, but also exhibits spatiotemporal heterogeneity, which are important factors contributing to drug resistance and poses a significant challenge to tumor immunotherapy. Therefore, the purpose of this review is to integrate the latest researches and conduct a systematic review of the dynamic evolution process in TIME, aiming to provide readers with a framework for understanding the dynamic evolution of TIME. This review comprehensively discusses the major regulators and networks involved in the dynamic evolution of the TIME, the spatiotemporal dynamics of TIME components, metabolic reprogramming as an engine of TIME evolution, the targeting of metabolic regulators, and niches for TIME modulation, clinical and translational challenges, and future prospects, clarifying the dynamic evolution of TIME and its role in the tumor pathogenesis and treatment resistance and exploring its significant potential for clinical translation in oncology.

## 2 Core Drivers and Frameworks of TIME Dynamic Evolution

**FIGURE 1:**
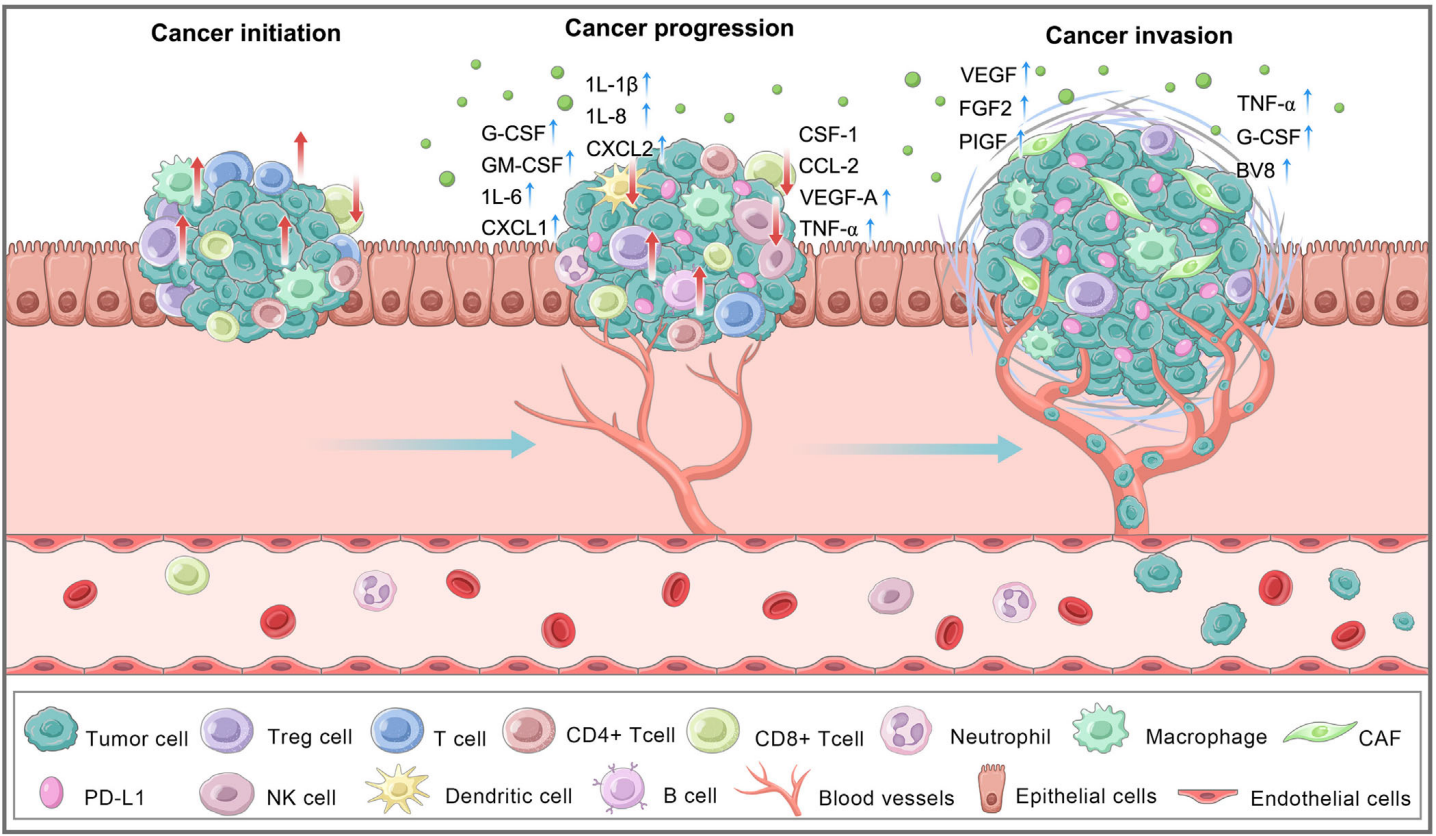
The evolution of TIME. The tumor immune microenvironment (TIME) plays an important role in the progression of cancer. The rapid progress of cancer cells requires the realization of immune attack to immune escape, accompanied by the continuous catalysis of inflammatory environment and the formation of blood vessels. With the formation of ECM remodeling and inhibited immune microenvironment in TIME, it is conducive to the invasion and metastasis of cancer.

The genetic alterations as well as the changes in immune cells and stromal cells in the microenvironment during tumor development, jointly influence the functional characteristics of the tumor. Therefore, the formation of TIME is a dynamic process (Figure 1). This phenomenon ultimately fosters an immunosuppressive microenvironment that supports tumor cell proliferation, invasion, and metastasis. In the following sections, we comprehensively discuss four key aspects: (1) tumor‐intrinsic drivers, (2) microenvironmental stressors, (3) reciprocal crosstalk among tumor–stromal–immune circuits, and (4) therapy as a selective pressure.

### 2.1 Tumor‐Intrinsic Drivers

**FIGURE 2:**
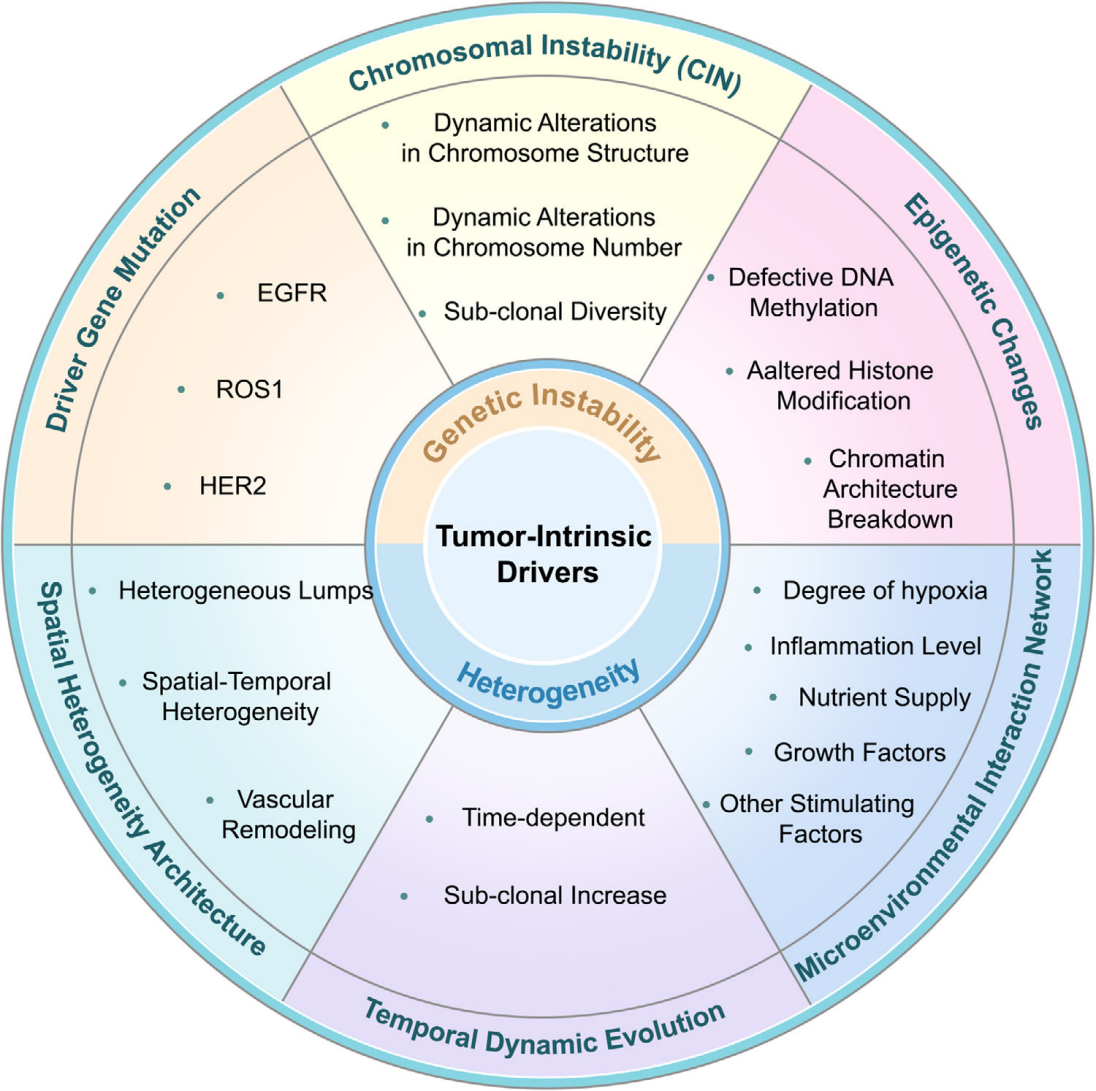
Tumor‐intrinsic drivers. The intrinsic driving factors of tumors mainly consist of two elements: genetic instability and heterogeneity. Further subdivided, genetic instability is composed of driver gene mutations, chromosomal instability, and epigenetic variations. Heterogeneity is constituted by spatial heterogeneity, temporal dynamic evolution, and microenvironmental interaction networks.

Mutations and suppression are endogenous forces that initiate cancer. Together, they control the evolutionary path of cancer and its immune response to drug therapies primarily by misregulating signaling pathways, metabolic programs, and apoptotic pathways (Figure 2).

#### 2.1.1 Genetic Instability

Intrinsic driver mechanisms include driver gene mutations, chromosomal instability (CIN), and epigenetic modifications, which are the basic molecular mechanisms of carcinogenesis. The accumulation of increasing driver gene mutations provides an abundant material base of genetic variation, which drives cancer evolution. Such variation can enable different cancer cell subpopulations to achieve various functions, such as proliferation, invasion, and survival. For example, the EGFR gene is mutated in various types of cancers, such as colorectal cancer and pancreatic carcinoma. These tumors respond poorly to anti‐PD‐1 antibody treatment [28, 29]. The reactive oxygen species (ROS)1 gene is overexpressed in 30–56% and 80–100% of primary brain tumor and metastatic oral squamous cell carcinoma patients, respectively, and is positively correlated with poor prognosis [30, 31]. In addition to dysregulated expression, genetic alterations in ROS1 include mutations, splice variants, gene amplifications, and fusions. The expression of the cancer‐associated gene HER2 is amplified in 15–20% of human breast cancers [32]. Different HER2 aberrations, including protein overexpression (2–35%), gene amplification (2–20%), and mutation (1–4%), have been reported in NSCLC [33]. HER2 protein overexpression is detected in 13–25% of locoregionally advanced/metastatic urothelial carcinomas and is commonly associated with poor prognosis [34].

CIN, characterized by dynamic alterations in chromosome structure and number, represents a higher‐order form of genomic disarray. Key mechanisms driving cancer evolution through CIN include whole‐genome doubling, somatic copy number alterations, and the emergence of subclonal diversity. Utilizing a retrospective simulation of randomized controlled trials based on real‐world cohorts, Thompson et al. [35] demonstrated that CIN signatures may serve as biomarkers capable of identifying patients who are nonresponsive to platinum‐based, taxane‐based, and anthracycline‐based chemotherapies.

Epigenetic changes differ from genetic mutations, which are irreversible and static. Epigenetic modifications lead to phenotypic plasticity by inducing differences in gene expression (e.g., epigenetic variations). Defective DNA methylation, altered histone modification, and chromatin architecture breakdown are collectively referred to as “epigenetic instability.” For example, constitutive activation of the Wnt/β‐catenin pathway in CSCs, caused by epigenetic dysregulation, results in self‐renewal, treatment resistance, and maintenance of heterogeneity [36]. A multiomic study published in 2025 revealed that tumor cells use epigenetic reprogramming for tumor cell‐based immune escape via bidirectional crosstalk between MDSCs and cancer cells, severely silencing the antitumor immune response. These findings offer an attractive rationale for pursuing integrated epigenetic‒immunotherapeutic approaches [37].

#### 2.1.2 Heterogeneity

Tumor heterogeneity, as a multidimensional, time‐varying complex systemic feature, consists of the integration of genotype and epigenetic heterogeneity based on spatial restriction, temporal fluctuation, and ecological selection. Analysis of heterogeneity from three aspects (spatial heterogeneity structure, temporal dynamic fluctuation, and the TME network) can reveal the complexity of heterogeneity.

*Spatially heterogeneous architecture*: Tumors are not clumps of homogeneous cells but rather a spatially organized, complex ecosystem. Using integrated multimodal imaging technologies, researchers in China constructed the first submicron‐resolution, comprehensive 3D pathological atlas of a mouse glioblastoma. This atlas showed the dynamic evolutionary characteristics of the TME, including vascular structures, cellular structures, and BBB permeability, offering a new means to explore heterogeneous tumors. This study revealed significant spatiotemporal differences in tumor invasiveness, vascular structures, and angiogenesis. In early stages, tumor regions were poorly defined and rough, whereas in advanced stages, they were well defined and smooth [38].

*Tumor‐bearing time*: Tumor heterogeneity is largely related to the tumor‐bearing time of the patient. As the duration of cancer progression increases, multiple subclones and subclonal mutations exist within the tumor, which drives the increase in tumor heterogeneity and affects the prognosis of patients [39, 40]. The TRACERx study conducted a longitudinal follow‐up on patients with non‐small cell lung cancer (NSCLC) who underwent early surgical resection [41]. It was found that copy number deletions and reduced transcriptional activity, which were closely related to clonal events, occurred simultaneously with the editing of new antigens [42]. Thus, this hypothesis was well explained.

*Microenvironmental interaction network*: The cell types in TIME are diverse, and different cell types are composed of heterogeneous subgroups with different phenotypes and functions, highlighting the complexity of TIME. Recent studies have shown that human breast cancer tissues contain approximately 14 different myeloid cell clusters and 17 different T cell clusters, which is about twice the amount found in normal breast tissues [43]. This indicates that immune cells exhibit significantly higher heterogeneity when activated [43].

### 2.2 Microenvironmental Stressors

#### 2.2.1 Hypoxic Microenvironment

Hypoxia is a classic and common phenomenon in the TME, which is closely related to the rapid proliferation of tumor cells and the insufficient oxygen supply. With the evolution of tumor growth, cells shift from normoxia to hypoxia. It has been shown that tumor cells respond mostly to oxygen levels, particularly hypoxia, primarily through hypoxia‐inducible factor (HIF) gene upregulation and stimulation of HIF signaling; the Nobel Prize in Physiology or Medicine was awarded for this discovery in 2019 [44, 45].

Hypoxia substantially remodels cancer cell biology in the TIME, thus affecting manifold aspects, such as cancer cell stemness, dormancy, redox adaptation, and intercellular communication. Moreover, hypoxia induces vascular ECs to upregulate the transcription of vascular endothelial growth factor (VEGF) and trigger excessive angiogenesis, which then orchestrates TIME dynamics and the response to treatment [46, 47, 48]. In addition to the VEGFA gene, Buffa et al. [49] reported a list of 15 hypoxia‐related genes: VEGFA, SLC2A1, PGAM1, ENO1, LDHA, TP11, P4HA1, MRPS1, CDKN3, ADM, NDRG1, TUBB6, ALDOA, MIF, and ACOT7. The subset of genes included in this list is referred to as the Buffa hypoxia signature and is employed as a predictor of hypoxic conditions [49].

Using the Buffa 15‐gene signature, Bhandari et al. [50] performed pancancer whole‐genome sequencing data analyses in various cancer types, including solid and hematological tumors, to explore the presence of hypoxia. The pancancer atlas revealed substantial intratumoral and interpatient heterogeneity in hypoxia both between cancer types and within individual tumor types [50]. For example, lung squamous cell carcinoma and cervical squamous cell carcinoma had the highest scores for hypoxia, and chronic lymphocytic leukemia and thyroid adenocarcinoma had the lowest scores. Greater variability in hypoxia was observed among cancer types with varying pathophysiologies (such as cholangiocarcinoma, B‐cell non‐Hodgkin lymphoma, and lung adenocarcinoma) [50, 51].

More importantly, increased hypoxia signaling with upregulated expression of hypoxia‐related genes is frequently correlated with increased genomic heterogeneity and poor overall survival (OS) and progression‐free survival (PFS) [52]. The inherent heterogeneity of the hypoxic microenvironment thus demonstrates the possibility of tailoring hypoxia‐targeting therapies in future clinical studies and treatments.

#### 2.2.2 Nutrient Deprivation

The TIME is often fed by a poorly developed or abnormal vasculature, which leads to an inadequate nutrient and/or oxygen supply and ineffective waste removal. A nutrient‐starved TIME induces a “war of attrition” between rapidly growing cancer cells and innate immune cells for bioenergetic substrates required for immune effector responses [53, 54]. Therefore, the intrinsic metabolic niche of the tumor may enable an immunosuppressive niche that needs to be targeted. However, the key point here is that those poor conditions push infiltrating immune cells to adopt “immune‐metabolism” adaptations related to an immunosuppressive phenotype. In turn, this metabolic reprogramming weakens the immune antitumor response.

### 2.3 Reciprocal Crosstalk: Tumor–Stromal–Immune Circuits

The tumor stroma is extremely important in the initiation phase of tumor development; indeed, tumor cells are capable of mobilizing fibroblasts to support tumor development. Fibroblasts stimulated into the TIME are known as CAFs. Compared with normal fibroblasts, CAFs exhibit differences in structure and function, including greater proliferation and motility. The mechanisms underlying fibroblast activation in the TIME remain unclear [55, 56]. Transforming growth factor‐β (TGF‐β), epidermal growth factor (EGF), platelet‐derived growth factor (PDGF), and fibroblast growth factor 2 (FGF2) secreted from CSCs play crucial roles in activating and recruiting CAFs. Recent research from an animal model has indicated the involvement of prostaglandin E2 (PGE2) and Wnt signaling [57, 58]. In addition, VEGFA signaling can also induce fibroblast activation to support cancer initiation [58, 59]. Therefore, the activation of CAFs also promotes tumor expansion and, in different cancers, has become an appealing therapeutic target (Figure 1).

CAF infiltration is often correlated with reduced antitumor immune reactions and poor prognosis. Inside the TIME in esophageal cancer, CAFs release the proinflammatory cytokine interleukin‐6 (IL‐6), which increases the frequency of Foxp3+ tumor‐infiltrating lymphocytes (TILs) and impairs the CD8+ T‐cell response [60]. High IL‐6 levels are also responsible for the production of indoleamine 2,3‐dioxygenase (IDO)‐producing regulatory DCs and monocyte‐derived MDSCs through the activation of signal transducer and activator of transcription 3 [61, 62]. Pancreatic cancer CAF‐secreted βig‐h3 protein directly inhibits CD8+ T‐cell proliferation in pancreatic cancer. Alternatively, βig‐h3 indirectly inhibits T‐cell cytotoxicity by increasing the expression of T‐cell immunoglobulin and mucin‐domain containing‐3 (TIM‐3), programmed death‐1 (PD‐1), cytotoxic T‐lymphocyte‐associated protein 4 (CTLA‐4), and lymphocyte‐activation gene 3 [63, 64]. CAF‐released PGE2 and IDO stimulate NK cell dysfunction. Together, these processes amplify the inhibitory effects of IL‐8‐induced M2‐polarized TAMs on NK cells, causing immunosuppression and mediating tumor immune escape in the TIME [65, 66].

Immune cells regulate the structure and function of the stroma in the TIME through multiple pathways. T lymphocytes play a role in matrix remodeling. For instance, Th2‐type cells promote matrix remodeling through the secretion of IL‐4, which not only affects the tissue repair process but also alleviates immunosuppression [67]. Macrophages and myeloid cells influence the formation of immunosuppressive stroma. For instance, in a follicular lymphoma model, TAMs indirectly regulate the phenotypes of stromal cells by maintaining B‐cell receptor activation, resulting in imbalances in chemokine secretion and alterations in the composition of the ECM [68]. MDSCs exert their immunosuppressive effects by inducing the expression of arginase 2 locally, thereby reducing the availability of arginine l‐Arg. This process indirectly affects the metabolic state of stromal cells and alters the immunosuppressive function of the stroma [69, 70].

### 2.4 Therapy as a Selective Pressure

The dynamic evolution of the TIME is jointly shaped by the intrinsic driving factors of tumors, the pressure of the microenvironment, and the complex reciprocal crosstalk among tumors, the stroma, and immunity. However, cancer treatments, as powerful external forces, exert very strong selective pressures, which in turn form the major driving forces for TIME adaptations and determine therapeutic outcomes. Cancer therapy directly eliminates specific sensitive subpopulations of tumor cells (through chemotherapy or targeted therapy). This treatment is frequently associated with either treatment resistance or relapse, because treatments that exert selective pressure will result in subpopulations with resistance to specific treatments, whether intrinsic (most often due to genetically encoded properties) or acquired. Such populations with strong immune evasion properties can now survive as outliers and thus become dominant clones. Moreover, traditional treatments such as radio‐ and chemotherapies damage the vasculature, worsening tumor hypoxia and inducing immune suppression; however, by promoting cell death, these treatments promote the release of large quantities of metabolites (such as lactate) into the tumor, modifying the nutrient distribution in the TME and resulting in the establishment of a metabolically hostile environment that inhibits immune function. Although various therapies result in the release of damage‐associated molecular patterns, such as DNA‐binding protein, HMGB1, and ATP, from eliminated tumor cells, which may act as potential immune‐activating signals, their continued release may paradoxically lead to chronic inflammation and immunosuppression.

In addition to conventional radiotherapy and chemotherapy, immunotherapy, which is a new paradigm for tumor treatment, can directly or indirectly alter the dynamic balance and functional status of immune cells in the TIME. Under this external therapeutic pressure, outcomes that are detrimental to efficacy arise: either persistent antigen exposure intensifies T‐cell exhaustion, or the therapy induces the release of large quantities of cytokines/chemokines (e.g., tumor necrosis factor [TNF]‐α, IL‐1β, IL‐6, IL‐10, and TGF‐β). These alterations remodel intercellular communication; recruit immunosuppressive cells (Tregs, MDSCs, and M2‐polarized TAMs); and ultimately establish an immunosuppressive microenvironment that is conducive to therapeutic resistance.

In summary, therapy‐related selective pressures lead to adaptive evolution of the TIME, resulting in ecosystem equilibrium, which is disadvantageous to antitumor immunity but favorable for tumor survival and expansion. Thus, it is vital to understand and opportunistically intervene in therapy‐triggered, selective pressure‐mediated adaptive TIME evolution to overcome the current therapeutic limitations and achieve a more durable response. This perspective leads beyond the single‐drug mindset and prompts the framing of treatment regimens as living strategies that transform over time and space.

## 3 Spatiotemporal Dynamics of TIME Components

As previously mentioned, the intrinsic characteristics of tumors drive tumorigenesis, whereas the intrinsic immune landscape of the TIME promotes further tumor progression. This process involves the infiltration of immune cells into the tumor site, the formation of blood vessels in the tumor, and the reconstruction of the ECM. Together, these three factors create a complex tumor immune landscape. To gain a deeper understanding of this landscape and analyze its functional dynamics, these three aspects are systematically discussed.

### 3.1 Immune Landscape: Sequential Infiltration and Functional Polarization

#### 3.1.1 M1/M2 TAMs

**FIGURE 3:**
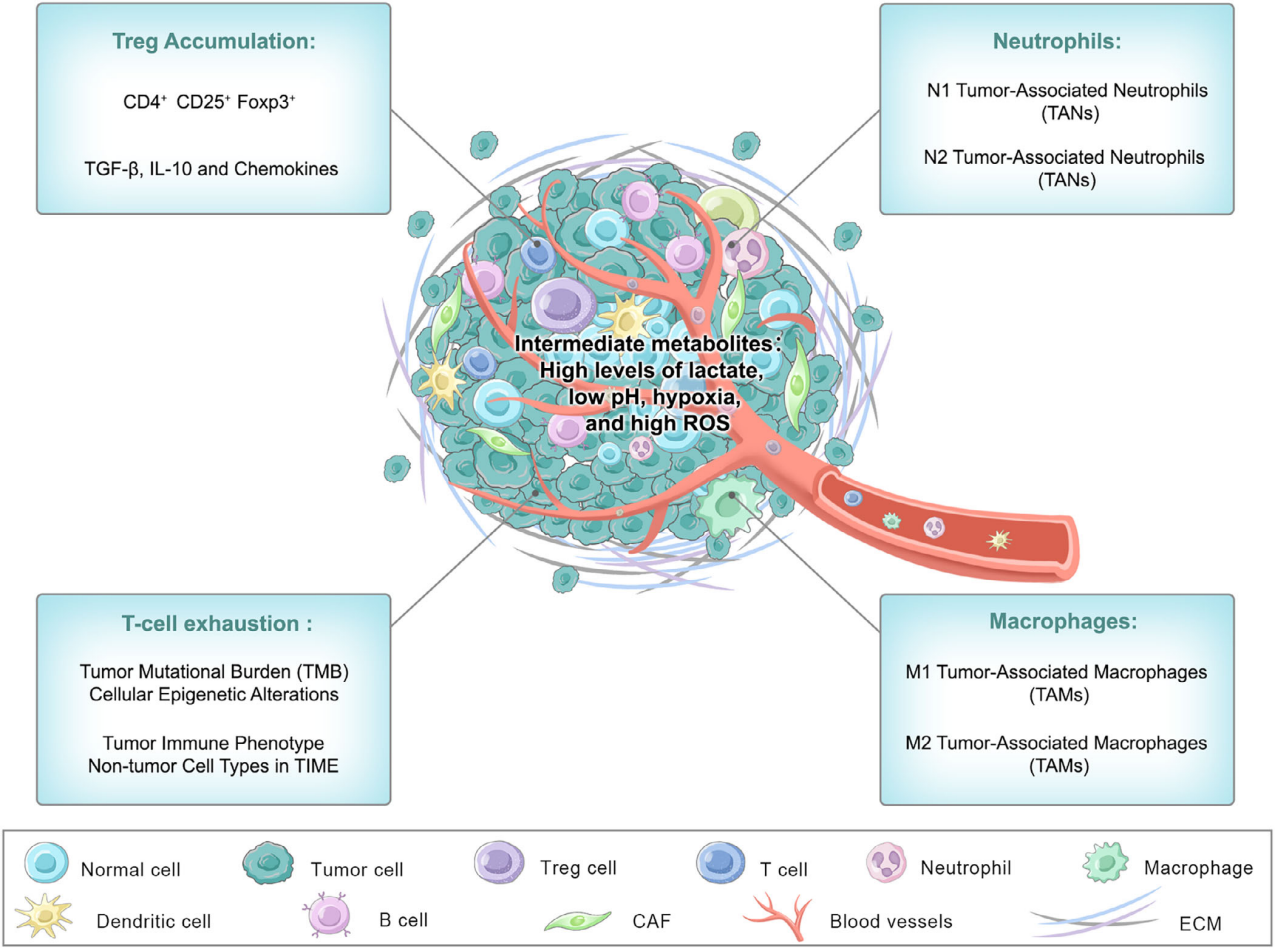
Changes of metabolism in the TIME. TIME includes cancer cells, a variety of immune cells, endothelial cells and extracellular matrix (ECM). The metabolic substrates, metabolic modes, and final metabolites of different cells are different. Cancer cells even suppress the function of cytotoxic T cells through competitive glucose uptake, ultimately leading to a tumor microenvironment that metabolizes high levels of lactic acid and low pH, hypoxia, and high levels of ROS, which in turn promotes cancer progression and immune escape.

Immunohistochemistry for a TAM marker (CD68) followed by CIBERSORT‐mediated deconvolution of gene expression profiles revealed that TAMs are the predominant myeloid cells infiltrating most human solid tumors [71, 72, 73, 74]. TAM populations show considerable dynamic heterogeneity within individual tumors and among patients [72, 75, 76]. This heterogeneity is, in part, a reflection of the plasticity that TAMs exhibit in adapting to a broad range of phenotypic states (proinflammatory M1‐polarized to anti‐inflammatory M2‐polarized), metabolism, and functional potentials in response to microenvironmental cues [77, 78]. Thus, particular TAM subsets may facilitate tumor induction and angiogenesis, therapeutic resistance, and disease progression, ultimately leading to poor clinical outcomes [73, 79, 80]. In contrast, specific subpopulations of TAMs express a tumoricidal phenotype and may synergize with immunotherapies [77, 81]. The location of TAMs within the TIME, therefore, becomes central to their protumorigenic or antitumorigenic functional output (Figure 3). In particular, TAMs localized in perivascular or hypoxic niches are typically immunosuppressive or proangiogenic [82, 83]. Conversely, TAMs inside cancer cell nests or at invasive tumor fronts have been shown to have tumor‐killing effects under specific circumstances [84, 85].

#### 3.1.2 N1/N2 Tumor‐Associated Neutrophils

Neutrophils constitute the largest subset of innate immune cells, and their physiological function contributes primarily to defending against pathogen invasion through phagocytosis and the generation of neutrophil extracellular traps (NETs) [86]. The functions of neutrophils are also considerably expanded in the TIME under different conditions. Neutrophils can directly contribute to resistance to cancer as well as tumor progression through activation. In an attempt to classify these disparate functional roles in cancer, Fridlender et al. [87] identified these polarized states with antitumorigenic N1 and protumorigenic N2 phenotypes (Figure 3), with the caveat that, in all likelihood, no one surface marker can unambiguously distinguish these two subpopulations.

In the first tumorigenic stage, neutrophils show characteristics that predispose them to CD8^+^ T‐lymphocyte activation and the induction of tumor cell apoptosis through the production of ROS and different cytokines; nevertheless, elements such as TGF‐β or granulocyte colony‐stimulating factor (G‐CSF) can induce their phenotypic activation toward the immunosuppressive N2 type [87]. Such N2‐polarized neutrophils aid in tumor progression by releasing neutrophil elastase (NE) and matrix metalloproteinases 8/9 (MMP8/9) for active ECM remodeling [88].

In addition, immunosuppressive N2 macrophages and Tregs are recruited to the TIME through cytokines (CCL2 and CCL17) and thus promote HCC development [89]. N2 neutrophils secrete IL‐8, which regulates the function of BCSCs through the activity of CXCR1/CXCR2, C‐X‐C motif chemokine receptors 1 and 2, and O‐N‐acetylglucosamine modification. A separate study revealed that this IL‐8 pathway ultimately contributes to immune escape and metastasis in breast, colon, and lung cancer [90, 91, 92].

#### 3.1.3 T‐Cell Exhaustion Dynamics

The tumor mutational burden (TMB) [93], somatic epigenetic modifications [94], tumor immune phenotype, and nontumor cell types in the TIME [95, 96] are currently established determinants that control the fate of tumor‐infiltrating T cells. Epigenetic modifications within T cells have been shown to be primary factors involved in all phases of the development of T cells, including T‐cell exhaustion. Such alterations in chromatin result in the induction of another set of transcription factors that are different from the transcription factors of effector T cells and thereby invoke metabolic remodeling, which results in T‐cell exhaustion and death. In fact, epigenetic mechanisms that control gene expression drive the conversion of effector T cells to exhausted T cells. Another example is impeded oxidative phosphorylation, which promotes this metabolic “signature” and effector T‐cell phenotype [94]. Additionally, continuing accrual of the DNA methylation program through DNA methyltransferase 3α (DNMT3A) limits the effector T‐cell response and pushes T cells toward exhaustion. Eliminating DNMT3A from progenitor‐exhausted CD8⁺ T cells in vivo restores responsiveness to immune checkpoint therapy even with persistent antigen exposure [97].

When tumor cells are killed by T cells in the TIME, increased extracellular potassium is released from necrotic cells into the tumor interstitial fluid, in turn metabolically reprogramming T cells to exhibit stem‐like qualities and self‐renewal but impairing T‐cell receptor‐mediated Akt–mTOR phosphorylation and effector function [98, 99]. In contrast, fast‐growing tumor cells actively consume glucose and secrete lactate. As lactate accumulates, the proliferation of T cells is prevented, forcing them to enter a hyporesponsive state [100]. Despite these issues, in the TIME in solid tumors, most of the limited numbers of TILs, for example, tumor‐associated macrophages (TAMs), CAFs, and MDSCs, succumb early to exhaustion in the immunosuppressive tumor environment. For example, in a hepatocellular carcinoma (HCC) murine model, M2 macrophage‐derived extracellular vesicles encapsulating miR‐21‐5p promoted CD8⁺ T‐cell exhaustion in the TIME by targeting YOD1 and activating the Yes‐associated protein (YAP)/β‐catenin pathway [101]. A TME with a high level of NETs is associated with the expression of multiple inhibitory receptors and the induction of an exhausted phenotype in both CD4⁺ and CD8⁺ T cells [102]. In conclusion, successive tumor evolution, along with changes in the TIME, leads to exhaustion and an impaired response in T cells, the collapse of antitumor immunity, and the emergence of immune tolerance and acquired resistance (Figure 3).

#### 3.1.4 Treg Accumulation Mechanisms

Tregs are specialized T lymphocytes endowed with strong immunosuppressive activities that play fundamental roles in the protection of the host against immunopathological diseases (Figure 3), that is, disorders linked to inappropriate/overactive immune responses to self and/or nonself antigens [103, 104]. CD25 has been described as a cell surface marker of Tregs by Sakaguchi et al. [105]. We subsequently reported that Foxp3, which is a transcription factor that is exclusively expressed on Tregs, programs genes to differentiate and establish the suppressive function of Tregs [106, 107]. Together, these findings establish the classic Treg profile (CD4^+^ CD25^+^ Foxp3^+^) [105]. The accumulation of Tregs in the TIME results in the formation of an immunosuppressive environment that contributes to therapeutic resistance. Studies have shown that CCL5 is released by CD133^+^ CSCs and attracts Tregs together, stimulating the proliferation of these Tregs and the generation of IL‐10 [108]. Accumulating Tregs are stimulated to invade tumors and produce MMP9 [108]. In addition, Treg populations may grow because of the effects of some cytokines in the TIME. For instance, cytokines such as TGF‐β and IL10 actively stimulate Treg growth [89]. Moreover, chemokines such as CCL2 and CCL17 also help to recruit macrophages and Tregs to the TIME, enabling the proliferation of liver HCC [89].

### 3.2 Angiogenesis: From Initiation to Abnormal Vascular Networks

#### 3.2.1 Vascular Endothelial Growth Factor

Angiogenesis is essential for tumor development. A neoplasm must develop an independent vascular architecture for survival if it grows larger than 1–2 mm and therefore must provide itself with oxygen and nutrients. Neoplastic tumor angiogenesis is thought to be an important mechanism through which micronodular lesions remain dormant and avoid progression to invasive cancer. This phenomenon is due in large part to hypoxia, as it is the fundamental driver of tumor angiogenesis. In response to hypoxia, activation of the angiogenic switch can be mediated by several molecules, but VEGF and its downstream cascades play key roles. VEGF overexpression is associated with poor clinical outcomes in patients with multiple malignancies [109, 110]. VEGF expression inhibits both angiogenesis and tumor growth in mouse models of disease when it is blocked by experimental means [111, 112]. These findings emphasize the role of VEGF as one of the key mediators of angiogenesis. In addition to VEGF, other proangiogenic factors, such as basic FGF2 and placental growth factor, and proangiogenic inflammatory molecules, such as TNF, BV8, and G‐CSF, also exist in tumors [113].

#### 3.2.2 Formation and Continuous Adaptation of the Vascular Network

Both cancer cells and host stromal cells shape the development and evolution of the vascular network in an environmentally dependent manner [113, 114]. By investigating cellular composition across 19 different types of cancer using genome‐scale functional and protein networks, Li et al. [115] quantitatively characterized the abundance of ECs and reported high heterogeneity among various tumor types. In particular, ECs account for only 0.5% of the cells in one type of cancer, in contrast to 6.6% of the TME in nasopharyngeal carcinoma and the greatest relative number of cells among all cancers in HCC in the studied cohort. The strong enrichment of ECs in liver tumors clearly indicates that ECs play important roles in local disease progression, which could stem from the specific organ‐related functions of these cells, such as the modulation of vasculo‐endocrine communication or formation of the immunosuppressive vasculature.

Moreover, TAMs facilitate the recruitment/activation of ECs via a variety of secreted factors, such as VEGFA and CXCL8 [116, 117, 118], to ensure an “economic benefit” for tumor growth by supplying tumor cells with nutrition. Notably, TAMs can maintain their protumorigenic effector role even in low‐oxygen areas. In the TIME, MDSCs increase epithelial‒mesenchymal transition (EMT) through the production of IL‐6, which supports CSC properties, increases angiogenesis, and promotes metastasis [119, 120]. CAFs increase angiogenesis through the secretion of angiogenic factors into the TIME [121].

Notably, the tumor vasculature and immune cells also interact bidirectionally. Angiogenesis induced by tumors actively contributes to immune escape and suppression. For example, tumor‐associated ECs frequently display reduced expression of vascular adhesion molecules (such as ICAM‐1 and VCAM‐1), which are required for the homing and trafficking of immune cells and act as a barrier to prevent immune cells from entering the tumor interior [122]. In contrast, IDO, TIM3, and PD‐L1, which inhibit immune responses (tumor vascularization), show upregulated expression during tumor vascularization [123].

#### 3.2.3 Vascular Normalization Window

The integrity of the circulatory system is vital for drug delivery and therapeutic effectiveness [124, 125, 126, 127, 128, 129, 130, 131]. Vascular hyperpermeability, impaired perfusion, and the tumor ECM make up the three major physical limits preventing adequate delivery of antitumor agents to tumors. Among them, vascular hyperpermeability, which is most detrimental to drug delivery, immediately decreases drug delivery efficiency. Abnormal perfusion results in increased intratumoral IFP, which presents a biological challenge [132]. The ECM represents one of the primary challenges associated with the constriction of the tumor vasculature and the trapping of therapeutics [133]. Numerous studies suggest that antiangiogenic treatment in combination with chemotherapeutics may have a positive influence because of the induction of vascular normalization and a decrease in IFP, resulting in modulation of the therapeutic effect [134]. Clinical trials using similar therapeutic strategies have yielded promising results. A phase 2 trial (NCT00035656) demonstrated that cediranib, an effective inhibitor of all three VEGF receptors (VEGFR‐1, VEGFR‐2, and VEGFR‐3), elicits vascular normalization and enhances blood perfusion in glioblastoma patients [135]. A second phase II trial (NCT05400070) in which perioperative sintilimab and anlotinib were combined with chemotherapy demonstrated a high rate of pCR in patients with resectable NSCLC and closely associated vascular normalization [136]. A major challenge with exploiting vascular normalization is in recognizing and targeting the brief therapeutic window (clinically measured as only a few days or a couple of weeks in length). Studies have recently investigated dynamic surveillance strategies (e.g., with imaging biomarkers, such as increased MRI parameters including Ktrans) or peripheral biomarkers (e.g., the Ang‐2/VEGF ratio and MMP expression) for evaluating the permeability and timing of treatment [137].

### 3.3 ECM Remodeling: Stiffness, Composition, and Signaling in Progression and Metastasis

#### 3.3.1 Stiffness of the ECM During Tumor Progression and Metastasis

Dynamic changes in the mechanics of the ECM are crucial factors that regulate malignant tumor growth and tumor spread. Pathological stiffening of the ECM (a commonly seen characteristic of many solid tumors, where the stiffness of the ECM in tumors is 8–10‐fold greater than that in normal tissues) has been reported [138]; this stiffening is caused by the additional crosslinking of collagen, deposition of matrix and structural remodeling [139].

Lysyl oxidase (LOX) family enzymes serve as pivotal catalysts for crosslinking, significantly increasing matrix rigidity and stability by facilitating covalent bond formation between collagen fibrils. A clear example occurred in the breast cancer cell model, where LOX mediated an increase in the ECM crosslinking density of highly migratory cells, resulting in a unique wavy fiber structure and an increase in hardness. The diameter of the collagen fibers in these matrices (1.24 ± 0.31 micrometers) was significantly smaller than that in the matrices of low metastasis cells (1.89 ± 0.42 micrometers) [138]. Furthermore, the expression of the IV type collagen subunit COL4A2 was upregulated by 2.3 times in the ECM of highly metastatic cells, and it showed a significant positive correlation with the content of hydroxyproline (*r* = 0.82) and the elastic modulus (*r* = 0.79). Silencing COL4A2 can reduce the elastic modulus of the ECM in highly metastatic cells by 37%, decrease the penetration rate of cancer cells by 61%, and reduce the number of metastatic nodules in the lungs of mice by 55% [138]. Overall, the increase in ECM hardness promotes the proliferation and invasion of cancer cells. On the other hand, cancer cells further recruit stromal cells to accelerate ECM remodeling, thereby forming a self‐reinforcing positive feedback loop.

Surprisingly, the mechanical properties of ECM are not uniform and exhibit temporal and spatial heterogeneity. At the core of the tumor, the fibers in the ECM are disordered and scattered; while near the tumor boundary, the fibers become dense and thick; at the front of tumor infiltration, the fibers show radial arrangement. These phenomena may be related to the escape of tumor cells [140].

#### 3.3.2 ECM Composition and Signaling

CAFs are the main effector cells that accelerate ECM remodeling. Activated CAFs form a tumor‐promoting microenvironment rich in dense fibers by secreting large amounts of type I/III collagen, fibronectin, and proteoglycans, and accompanied by the upregulation of MMPs and their inhibitors. Analysis of samples from melanoma and breast cancer patients revealed that the ECM fibers were arranged radially in the outermost region of the tumors, and this arrangement structure ultimately facilitated the spread of cancer cells [140]. The highly structured ECM has the ability to transmit mechanical signals and directly activate the expression of genes promoting invasion in cancer cells. Furthermore, CAFs exert contractile force on the basement membrane by secreting proteases, creating discontinuous areas. Cancer cells can utilize these areas for migration [141, 142, 143].

Except CAFs, macrophages are also one of the sources for increased collagen in the ECM. Studies have shown that an increase in ECM stiffness can induce macrophages to take up arginine, thereby promoting an increase in collagen and affecting the composition of the ECM [144]. Strangely, macrophages can also utilize the amino acids produced by collagen degradation for arginine biosynthesis. However, another study has found that an increase in intracellular arginine levels stimulates the production of inducible nitric oxide synthase (iNOS) and reactive nitrogen species, promoting collagen deposition and fibrosis in pancreatic stellate cells [145]. In addition to CAFs and macrophages, neutrophils can remodel the ECM structure by secreting MMP8/9 and NE, not only accelerating angiogenesis but also significantly enhancing the invasiveness and progression of malignant tumors [146, 147].

## 4 Metabolic Reprogramming as the Engine of TIME Evolution

Metabolic reprogramming is one of the important markers of malignant tumors. During tumor proliferation, tumor cells preferentially utilize glucose, which is known as the Warburg effect [148, 149, 150, 151, 152, 153, 154]. In the TIME complex environment, the metabolic characteristics exhibited by different cell types vary. The glycolytic pathway dominates the energy supply for cells such as T cells and B cells; fatty acids dominate the energy supply for Tregs and M2‐type macrophages [155]. Nutritional competition in the microenvironment leads to acidosis in the cancer ecosystem, and together with hypoxia and high levels of ROS, it hinders the function of immune cells [156]. In a study on renal cell carcinoma, it was found that the infiltration level of CD8⁺ T cells was significantly negatively correlated with the expression of glucose transporter 1, indicating that glucose metabolism in the TME affects the infiltration of effector T cells [157]. In summary, the metabolic products between cells can affect the dynamic evolution of TIME (Figure 2).

### 4.1 Nutrient Competition and Metabolic Adaptations

#### 4.1.1 Glucose Metabolism

Malignant tumor cells prefer glycolysis over oxidative phosphorylation as the primary energy metabolism pathway for maintaining their proliferation and metastasis [148]. This shift largely occurs because compared with oxidative phosphorylation, glycolysis produces adenosine triphosphate (ATP), which is necessary to meet the increased bioenergetic needs of uncontrollable proliferation [158, 159]. Furthermore, cancer cells exploit alternative biosynthetic pathways, such as the pentose phosphate pathway and serine metabolism, to provide the needed macromolecular precursors that underwrite cell replication [149, 150].

#### 4.1.2 Amino Acid Metabolism

In addition to the catabolism of glucose to produce ATP, malignant cells use glutamine, serine, arginine, fatty acids, and lipids for rapid cell proliferation [160]. Under conditions of nutrient starvation, for example, glucose or glutamine starvation, cancer cells upregulate de novo serine synthesis by controlling the transcription of master metabolism enzymes in the serine synthesis pathway, such as PHGDH, PSAT1, and PSPH. This mechanism maintains the redox balance, mobilizes the oncogene c‐Myc, and ultimately drives survival in tumor cells [151]. Evidence suggests that cancer cells have unusually high levels of glutamine uptake. This specific preference for nutrients is programmed in cells from the outset through mTORC1 signaling in the metabolic roles of glucose and glutamine. As inhibiting glutamine uptake promotes the utilization of glucose by resident cellular populations, glutamine metabolism thus appears to inhibit glucose uptake, and TIME glucose availability is less constrained. Consequently, intrinsic programs in resident cells result in the selective uptake of glucose by immune cells and of glutamine by cancer cells. This cell‐selective partitioning of nutrients might be exploited to design therapeutic and imaging strategies for increasing and tracking metabolic programs and the activities of distinct cellular subsets within the TIME [161].

#### 4.1.3 Lipid Metabolism

Lipid metabolism regulates tumor growth, metastasis, and recurrence [162]. CD8^+^ T cells can recognize cancer‐specific antigens and destroy cancer cells through effector molecules such as perforin and granzyme, which are positively correlated with patient prognosis. Although CD8^+^ T cells typically use aerobic glycolysis to maintain effector function, studies have shown that an increase in the concentration of free fatty acids in the blood circulation or TIME can lead to a decrease in CD8^+^ T lymphocyte activity [163]. High cholesterol levels are positively correlated with CD8^+^ T‐cell depletion [52]. For example, in melanoma mouse models, cholesterol induces endoplasmic reticulum stress and activates the X‐box‐binding protein 1 signaling pathway, thereby promoting the upregulation of programmed death receptor‐1 (PD‐1) expression on the surface of CD8^+^ T lymphocytes, which may lead to better clinical benefits if immunotherapy is combined with cholesterol‐lowering therapy [164]. In addition to CD8^+^ T cells, other immune cells in the TIME also support lipid uptake and transport. For example, in triple‐negative breast cancer, CAFs increase the uptake of foreign fatty acids in the TIME by upregulating fatty acid transporter family protein (FATP)1 expression [165] or promote the proliferation of cancer cells by transferring lipids to cancer cells through exosomes [166]. Hypoxia and glucose deficiency in the TIME also promote TILs to maintain energy levels and effective function by increasing fatty acid intake and catabolism, changing the original manner in which oxidative phosphorylation is relied upon [167]. Changes in lipid metabolism are important indicators of tumor progression and immune cell growth and function in the TIME (Figure 2).

### 4.2 Key Metabolites as Signaling Hubs and Effectors

#### 4.2.1 Lactate

Lactic acid has long been considered a byproduct of cell metabolism in the TIME (cancer cells, stromal cells, and immune cells) [168], and new evidence suggests that lactic acid may be a metabolite in the TIME, promoting TAM polarization toward the M2 type in the microenvironment [169, 170] and the secretion of a variety of cytokines related to metabolic function, such as IL‐6, TNF, and CCL5, thereby directly or indirectly accelerating glycolysis in tumor cells [171]. Moreover, in a high‐lactate microenvironment, the survival ability of Tregs is enhanced, and this phenomenon is closely related to the dual regulatory mechanism mediated by Foxp3. By inhibiting the expression of the Myc gene and downregulating the glycolytic process, Foxp3 promotes the continuous accumulation of lactic acid in the microenvironment. This accumulation of acidic metabolic products, in turn, provides suitable conditions for the survival of Tregs. This metabolic–immune interaction ultimately results in an immunosuppressive microenvironment that is conducive to the escape of cancer cells [172].

Recent research has shown that lactic acid triggers the posttranslational modification of proteins under the action of enzymes and participates in gene transcription regulation, tumor angiogenesis, and immune cell functions in the microenvironment. In the TIME, the intracellular accumulation of lactic acid increases the expression of VEGF and promotes tumor angiogenesis [173]. Moreover, it leads to lactylation of the histone H3K18la site, enhances M2 polarization of TAMs through a series of signals, and promotes tumor progression [174]. Some scholars have reported that lactylation enhances the immunosuppressive function of Tregs, and together with MDSCs, they form a barrier for tumor immune escape [175].

#### 4.2.2 Reactive Oxygen Species

Recent data indicate that ROS, which are biologically active molecules with strong oxidizing activity, can have toxic effects on various biological macromolecules through pathways such as oxidative DNA damage, protein denaturation, and lipid peroxidation. Clinical studies have confirmed that imbalances in ROS homeostasis are prevalent in patients with malignant tumors. The characteristic manifestations include a significant increase in the intracellular ROS concentration. The pathological mechanism involves mainly the compensatory downregulation of endogenous antioxidant enzyme systems (such as SOD and CAT) and the concurrent occurrence of multiple oxidative stress responses, including mitochondrial electron transport chain dysfunction, abnormal NADPH oxidase (NOX) activity, and excessive activation of the cyclooxygenase (COX) pathway [176], which are strongly associated with tumorigenesis, immunosuppression, and TIME reprogramming [177]. HIF stabilization under hypoxic conditions requires an increase in mitochondrial ROS, which further leads to autophagy and carcinogenicity [178, 179]. Moreover, key signaling molecules involved in the processes of tumor occurrence, development, and metastasis (including PDGF, integrin, granulocyte‒macrophage colony‐stimulating factor [GM‐CSF], γ‐interferon and TGF‐β) can exert bidirectional regulatory effects on NOX‐dependent ROS production in tumor‐infiltrating immune cells. Notably, changes in the level of ROS in the TME can significantly influence the functional state of myeloid suppressor cells through feedback mechanisms while regulating the phenotypic polarization of TAMs, the activation of CAFs, and the immune response of T lymphocytes [180, 181]. Therefore, targeting tumor ROS can provide a new direction for clinical treatment.

### 4.3 Emergence of Pathological Niches Driven by Metabolism

**FIGURE 4:**
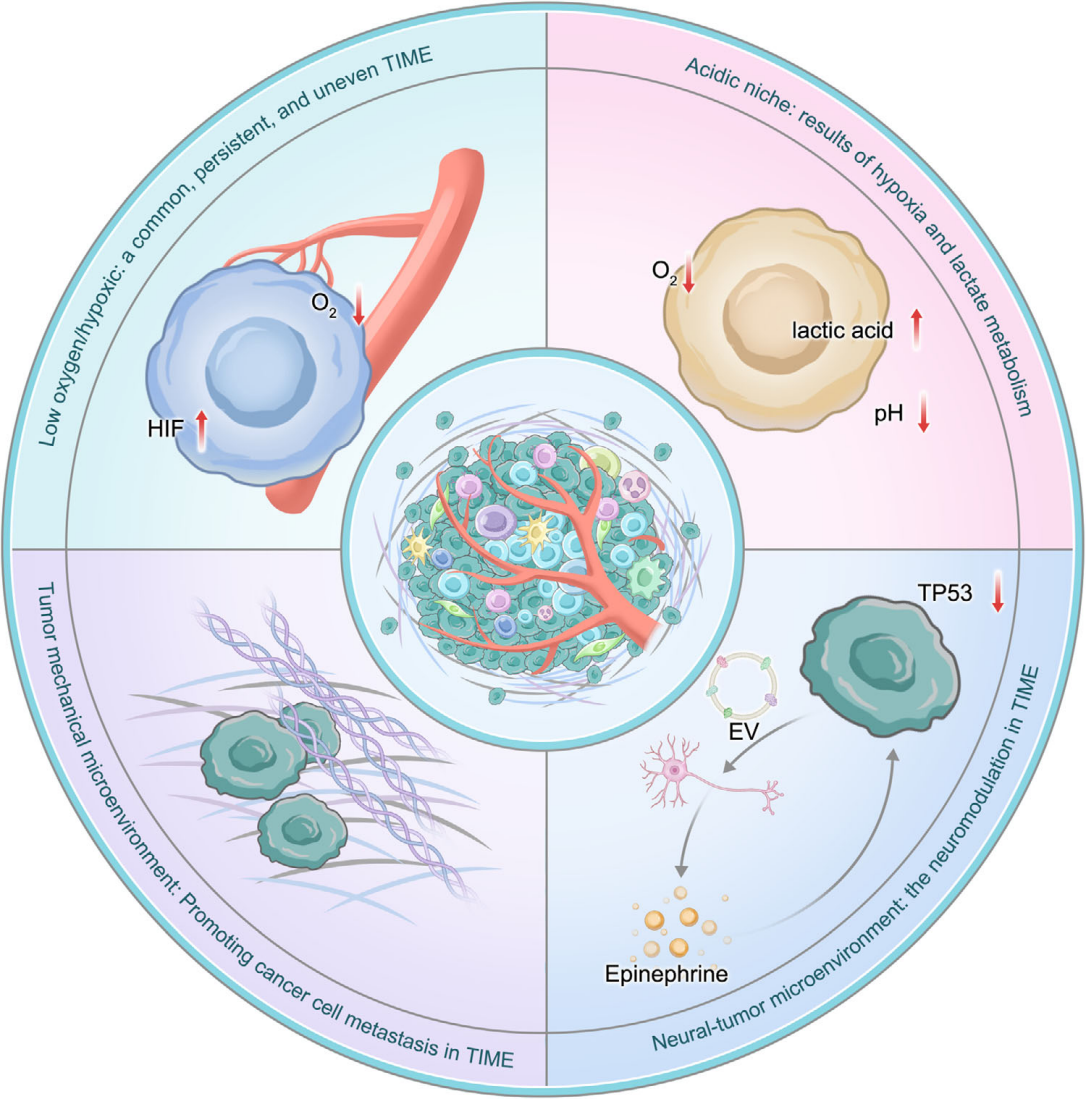
The formation of niche in TIME. Anoxic niche, acid niche, innervation niche, and mechanical niche significantly affect the development of cancer. However, the interaction forms of these microenvironments are complex, and only a clear understanding of the functions of each microenvironment and their interactions can provide a basis for later combination therapy.

Many studies have confirmed that anoxic [182, 183], acidic [184, 185], innervation [186, 187, 188, 189, 190], and mechanistic niches [191, 192, 193, 194, 195] significantly influence cancer development (Figure 4). However, these niches interact in complex forms, and only a clear understanding of the function of each niche and the crosstalk among them can provide the basis for relevant combination therapies.

#### 4.3.1 The Acidic Niche: The Result of Hypoxia and Lactic Acid Metabolism

An imbalance in pH is among the important features of cancer. Malignant tumor cells exhibit characteristic acid‒base imbalance phenomena. The intracellular pH of these cells is significantly higher than that of normal somatic cells, while the extracellular microenvironment presents an abnormal acidic state. Studies have shown that this intracellular alkalization phenomenon plays a crucial regulatory role in the evolution of tumors through mechanisms such as activating glycolytic metabolic pathways, enhancing cell proliferation and migration capabilities, and inhibiting programmed cell death signal transduction. The formation of this acidic niche is closely related to the anoxic and metabolic microenvironments, as an anoxic microenvironment leads to increased lactic acid production, lactic acid metabolism, and carbon dioxide hydration [183]. Studies have shown that excessive lactic acid in the TIME is exported by monocarboxylic acid transporter (MCT4) and enters cancer cells through the cotransport of MCT1 and H^+^, reducing the pH in cancer cells and leading to cancer cell acidosis, a process that subsequently promotes the invasion and metastasis of cancer cells [196]. In addition, acidosis in the TIME has a regulatory effect on immune cells. For example, a low‐pH environment induces macrophages to differentiate toward the M2 phenotype and can activate neutrophils or DCs to inhibit the cytotoxic activity of TILs [197]. Moreover, acidic niches have synergistic effects on lactic acid metabolism, providing a supportive microenvironment for cancer development.

#### 4.3.2 Innervation Niche: Neuromodulation in the TIME

As an integral component of the TIME, the nervous system niche is defined as the unique microenvironment structure in which the nervous system regulates the biological behavior of tumors through certain mechanisms. Clinical studies have shown that the disordered functions of the autonomic nervous system are closely associated with the occurrence, progression, metastasis, and spread of several common solid tumors and hematologic malignant diseases and can also independently influence the clinical outcome of patients [198, 199, 200]. Recent studies have further revealed that tumor‐associated nerves play a role in regulating antitumor immune activity. Neurotransmitters (such as norepinephrine) and neuropeptides released by tumor‐associated nerves can directly act on immune cells, inducing T‐cell exhaustion and inhibiting the function of effector cells. This state of depletion weakens the efficacy of immune checkpoint inhibitors such as PD‐1/PD‐L1 [201, 202, 203]. Sensory nerves reduce the activity of CD8⁺ T cells in the TME by releasing neuropeptides and simultaneously decreasing the proportion of Th1‐type CD4⁺ T cells, thereby promoting immune escape. Surgical or pharmacological blockade of sensory nerves can increase T‐cell activity [204, 205]. Tumors infiltrate nerves and glial cells and express immune checkpoint molecules (such as IGSF9) on their surfaces. These molecules directly inhibit T‐cell activation. Targeting IGSF9 can restore T‐cell function and inhibit tumor growth, suggesting that IGSF9 is a novel immune checkpoint target [206]. Activation of the sympathetic nerve can recruit MDSCs, further enhancing T‐cell exhaustion [207]. Tumor‐associated nerves inhibit antitumor immunity through the accumulation of Blmp1⁺ Treg cells [208]. Glial cells regulate T‐cell function through the neuroimmune circuit [209]. Neural signals limit T‐cell infiltration and reduce the efficacy of immunotherapy by modulating tumor metabolism (such as glycolysis mediated by ENO1) and vascular abnormalities (such as adhesion molecules regulated by COUP‐TFII) [210, 211].

#### 4.3.3 Mechanistic Niche: Promotion of Cancer Cell Metastasis in the TIME

The mechanical microenvironment in the TIME is very important for tumor invasion and metastasis [186, 187, 188, 189, 190]. The structural composition of this mechanical microenvironment mainly includes the following four aspects: intracellular structural proteins (such as vimentin, actin, and neurofilament), ECM components (including collagen and fibrin), signaling molecules that mediate cell communication (represented by integrins), and stromal cell populations (mainly fibroblasts) [212]. Studies have shown that when cancer cells or activated stromal cells change their microenvironment through ECM remodeling, the mechanical properties of interstitial tissue can undergo significant changes, specifically through dynamic changes in physical parameters such as tissue stiffness and the elastic modulus [213, 214]. For example, CAFs can jointly regulate the biological activities of matrix components such as collagen and fibronectin by releasing bioactive molecules such as MMPs and TGF‐β. These cells also mediate the abnormal metabolism of hyaluronic acid, resulting in excessive deposition and abnormal crosslinking phenomena, ultimately leading to dynamic remodeling and degradation of the ECM. Such pathological microenvironmental changes can further drive EMT and enhance the self‐renewal and stemness maintenance capabilities of tumor stem cells [215, 216, 217, 218]. Increased matrix hardness can upregulate angiogenesis through the mechanosensitive signaling pathway mediated by MMP activity in ECs [219]. The mechanical microenvironment affects cell morphology, tumor suppressor secretion, and tumor treatment response in the TIME. A high‐stiffness microenvironment can induce tumor cells to release cytokines and recruit immune cells [220, 221]. Before invasion by human breast cancer cells, the number of macrophages is greatest when the matrix is the stiffest and when the TGF‐β signal is the strongest. Similarly, in a mouse breast cancer model, high levels of collagen were observed to upregulate the expression of COX2 and promote the release of secretory cytokines, thereby increasing the recruitment of macrophages and neutrophils. These results confirm that the stiffness of the matrix results in the formation of a mechanical niche that regulates the activity and function of tumor cells and immune cells in the TIME.

## 5 Targeting Metabolic Drivers and Niches for TIME Modulation

**FIGURE 5:**
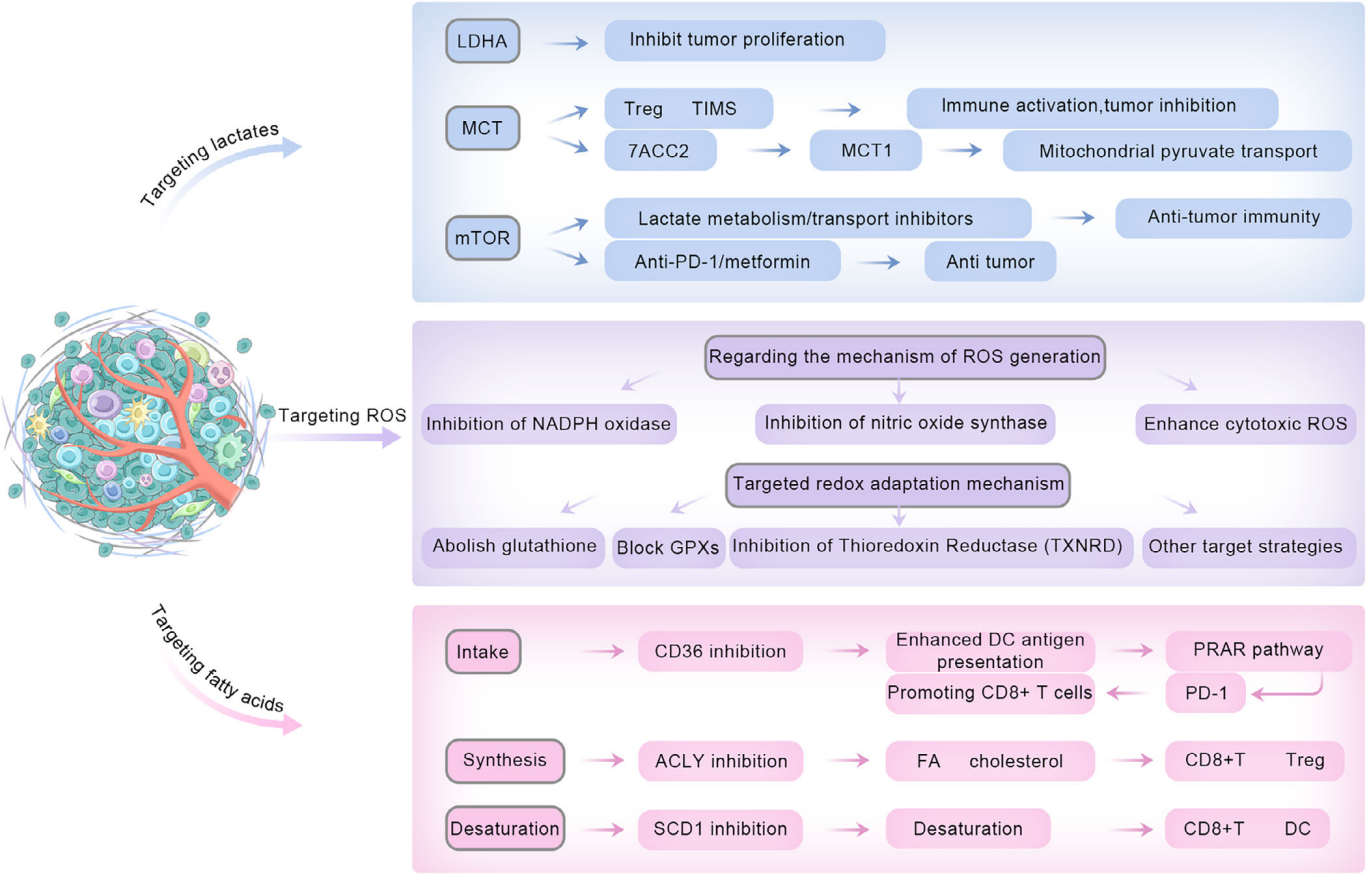
Therapeutic strategies in targeted TIME. According to the TIME changes in cancer progression, such as the accumulation of lactic acid in the microenvironment promotes immunosuppression; abnormal lipid metabolism in cancer cells and immune cells in microenvironment; changes in the polarization type of macrophages lead to the creation of immunosuppressive environments, and so on, resulting in therapeutic strategies that regulate these changes, thereby promoting the effectiveness of cancer treatment.

Tumor cells and their nontumor cells shape an immunosuppressive microenvironment through metabolic reprogramming. This section will systematically review therapies targeting key metabolites and effectors and further explore how to rationally combine these therapies with existing immunotherapies or other targeted therapies, with the aim of synergistically reshaping the TIME, overcoming immune resistance, and ultimately enhancing the depth and persistence of the antitumor immune response (Figure 5 and Table 1).

**TABLE 1:** Targeted drugs that inhibit metabolic molecules in TIME.

Targeting the lactate metabolism pathway				
Targeted molecules	Drug use	Tumor type	Clinical phase	Clinical serial number
MCT1	AZD3965	Advanced tumor	Phase I	NCT01791595
mTOR	SAR245409	Solid tumors	Phase Ib	NCT01390818
	TAK‐228	Soft tissue sarcomas	Phase II	NCT02987959
	Sirolimus	Advanced solid tumors/NSCLC	Phase I	NCT05840510
	Sirolimus	Malignant peripheral nerve sheath tumors	Phase II	NCT03433183
	BEZ235	Advanced solid tumors	Phase I	NCT01343498
	Everolimus	Diffuse large B‐cell lymphoma	Phase Ib/II	NCT01854606
	AZD2014	Solid cancer	Phase II	NCT03166904
	AZD2014	Endometrial carcinoma	Phase I/II	NCT02730923
	AZD2014	Gastric cancer	Phase II	NCT03061708
	VS‐5584	Nonhematologic malignancies or lymphoma	Phase I	NCT01991938
	AZD2014	Gastric cancer	Phase II	NCT03082833
	P7170	Solid tumors	Phase I	NCT01762410
	AZD2014	Prostate cancer	Phase I	NCT02064608
	PF‐04691502 And PF‐05212384	Endometrial cancer	Phase II	NCT01420081
	Rapamycin	Solid tumors	Phase I	NCT00337376
	MLN0128	Prostate cancer	Phase II	NCT02091531
	RAD001	Solid tumors	Phase Ib/II	NCT00985374
	BEZ235	Pancreatic neuroendocrine tumors	Phase II	NCT01658436
	RAD001	Solid tumors		NCT01341834
Targeting intracellular oxidative homeostasis				
Targeted molecules	Drug use	Tumor type	Clinical phase	Clinical serial number
ROS	ROS‐scavenging nanoenzymes	Head and neck cancer		NCT07064577
	ROS‐scavenging nanozyme	Head and neck cancer		NCT07086638
	ROS‐scavenging amino acid‐derived lipids	Head and neck cancer		NCT07081074
	MIT‐001	Head and neck cancer	Phase II	NCT04651634
NOX	NOX A12	Multiple myeloma	Phase IIA	NCT01521533
	NOX A12	Lymphocytic leukemia	Phase IIA	NCT01486797
	NOX‐H94	Cancer	Phase IIa	NCT01691040
	NOX‐A12	Colorectal and pancreatic cancer	Phase I/II	NCT03168139
	NOX—A12	Pancreatic cancer	Phase II	NCT04901741
	NOX‐A12	Glioblastoma	Phase I/II	NCT04121455
	APX‐343A	Solid tumors	Phase I	NCT07123415
NOS	LCI‐PED‐NOS‐EXER‐001	Tumors		NCT05058508
	L‐NMMA	Triple negative breast cancer	Phase Ib/II	NCT02834403
	NG‐nitro‐l‐arginine	Solid tumors	Phase I	NCT01324115
GSH	2B3‐101	Breast cancer		NCT01818713
	GSH	Ovarian cancer	Phase III	NCT02311907
	GSH	NSCLC		NCT06896422
	GSH	Hepatocellular carcinoma		NCT02321579
	NOV‐002	Ovarian cancer	Phase II	NCT00345540
	NOV‐002	Breast cancer	Phase II	NCT00499122
	2B3‐101	Glioma	Phase I/​IIa	NCT01386580
	N‐acetyl cysteine	Polycystic ovary syndrome		NCT06836128
Targeting lipid metabolism				
Targeted molecules	Drug use	Tumor type	Clinical phase	Clinical serial number
Fatty acids	Omega 3	Breast cancer		NCT02295059
	Omega‐3	Cancers	Phase I/II	NCT00003077
	Omega‐3	Gastric cancer		NCT01910948
	Polyunsaturated fatty acids	Prostate cancer		NCT00458549
	Omega‐3	Breast cancer		NCT02278965
	Omega‐3	Breast cancer		NCT00114296
	Omega‐3	Lung cancer	Phase II	NCT03936621
	Omega‐3	Breast cancer		NCT04268134
	Eicosapentaenoic acid (EPA)	Colorectal cancer		NCT01070355
	Omega‐3	Colorectal adenoma		NCT06427109
	Omega‐3	Colorectal cancer		NCT03661047
	Valproic acid	Cancers	Phase I	NCT00496444
	Omega‐3	Breast cancer	Phase II	NCT01869764
Fatty acids synthase inhibition	Omeprazole	Prostate cancer	Phase II	NCT04337580
	PPIs	Breast cancer		NCT02595372
Fatty acid desaturase	MTI‐301	Solid cancers	Phase I	NCT06911008
Signal blocker	RGX‐104	Lung cancer		NCT05911308
	RGX‐104	Lung and endometrial cancer	Phase I	NCT02922764
Targeting hypoxia and angiogenesis				
Targeted molecules	Drug use	Tumor type	Clinical phase	Clinical serial number
HIF	DFF332	Renal cancer	Phase I/Ib	NCT04895748
	EZN‐2968	Solid tumors		NCT01120288
	PT2977, MK‐6482	Clear cell renal cell carcinoma	Phase II	NCT03634540
	MK‐6482	Renal cell carcinoma	Phase II	NCT04489771
	NKT2152	Clear cell renal cell carcinoma	Phase I/II	NCT05119335
	MK‐6482	Renal cell carcinoma		NCT04195750
	MK‐6482	Clear cell renal cell carcinoma		NCT05899049
	NKT2152	Clear cell renal cell carcinoma	Phase II	NCT05935748
	MK‐6482	Renal cell carcinoma	Phase I/II	NCT05468697
	MK‐6482	Clear cell renal cell carcinoma	Phase III	NCT05239728
	MK‐6482	Renal cell carcinoma	Phase 1	NCT05030506
Antiangiogenesis	VEGF trap	Ovarian epithelial cancer, peritoneal cancer, or fallopian tube cancer	Phase I/II	NCT00436501
	AI‐081	Solid tumors	Phase I/II	NCT06635785
	Avastin, IBI305	Hepatocellular carcinoma	Phase I/II	NCT06537908
	AK112	Solid tumors	Phase 1a/1b	NCT04047290
	Bevacizumab	Pancreatic cancer	Phase II	NCT00066677
	Ivonescimab	Hepatocellular carcinoma	Phase II	NCT06375486
	VEGF trap	Breast cancer	Phase II	NCT00369655
	VEGF Trap	Colorectal cancer	Phase II	NCT00407654
	Avastin	Rectal cancer	Phase II	NCT00113230
	VEGF trap	Endometrial cancer	Phase II	NCT00462826
	Ivonescimab	Nasopharyngeal carcinoma	Phase II	NCT07064902
	VEGF trap	Gliomas	Phase II	NCT00369590
	AZD2171	Liver cancer	Phase II	NCT00427973
Targeting acidic microenvironment				
Targeted molecules	Drug use	Tumor type	Clinical phase	Clinical serial number
CAIX	68Ga‐OncoCAIX	Cancers	Phase I	NCT06840548
	SLC‐0111	Pancreatic ductal cancer	Phase 1b	NCT03450018
	18F‐VM4‐037	Kidney cancer	Phase II	NCT01712685
	[F‐18]VM4‐037	Cancers		NCT00884520
	cG250	Renal cell carcinoma		NCT00520533

Data in table from ClinicalTrials.gov.

### 5.1 Targeting Lactate Pathways

#### 5.1.1 LDHA Targeting

Increased expression of LDHA is strongly correlated with poor prognosis in patients with almost all types of malignant tumors. The inhibition of LDHA activity has become a successful therapeutic method for tumor inhibition. The competitive inhibition of the LDHA substrate pyruvate by oxamate, which can effectively inhibit the abnormal proliferation of gastric cancer cells, has been studied and confirmed [222, 223]. A competitive inhibitor of the NADH binding site is also effective for inhibiting tumor cell proliferation and disease progression [224].

#### 5.1.2 MCT Targeting

Lactate metabolism symbiosis in the TME is essentially induced by a lactate‐secreting mechanism regulated by the monocarboxylate transporters MCT4 and MCT1 [225]. Clinical studies have demonstrated that the upregulation of MCT1 and MCT4 expression is ubiquitous in numerous malignant human tumors [226]. In view of the key role of these two transporter proteins in the regulation of lactate homeostasis, utilizing the metabolic interaction pathways affected by MCT to overcome tumor chemoresistance is a promising strategy. In particular, the recent development of MCT subtype‐selective inhibitors, such as the small‐molecule compound 7ACC2, has suggested tremendous therapeutic potential, as 7ACC2 exerts antitumor effects on a broad spectrum of solid tumors through the dual inhibition of MCT1 and the mitochondrial pyruvate transporter [227, 228]. Other high‐selectivity MCT1 inhibitors, including AR‐C155858, AZD3965, and BAY‐8002, not only have inhibitory effects on MCT1 but also modulate the antitumor response via immune regulatory mechanisms [229, 230]. Notably, in addition to directly reducing tumor metabolism, MCT‐targeted therapy can redirect tumor immune escape through dual actions. We have demonstrated that Tregs and TAMs support their immunosuppressive state via the MCT1‐mediated uptake of lactate and that MCT1 deficiency can effectively inhibit the infiltration of these immunosuppressive cells by suppressing infiltration and switching the immunosuppressive state, restoring antitumoral immunity in the hypoxic/high‐lactate TME [231, 232]. Similarly, preclinical data support that MCT1 inhibitors selectively inhibit tumor growth but spare the functional status of CD4^+^/CD8^+^ T cells and potentiate the synergistic effects of targeting tumor cells and regulating the TIME [233]. These results indicate that further detailed research on the multidimensional roles of the MCT family in metabolic reprogramming and immune regulation networks of the TME will contribute the most to the optimization of precise tumor treatment.

#### 5.1.3 mTOR Targeting

Research has indicated that mTOR signaling plays a crucial role in mediating the regulation of lactate metabolism. Although mTOR inhibitors alone have limited anticancer effects, mTOR inhibitors have synergistic cancer activity with anti‐PD‐1 antibodies or other treatments (such as metformin) [234, 235]. Therefore, mTOR inhibitors should be combined with lactic acid metabolism/transport inhibitors to enhance anticancer immunity.

### 5.2 Targeting ROS Homeostasis

Under normal physiological conditions, ROS are crucial for activating and differentiating immune cells; however, many tumor types are characterized by excessively high levels of ROS, which can lead to numerous pathological consequences, such as oxidative stress, lipid peroxidation, protein oxidation, and DNA damage, resulting in either immune cell dysfunction or death [236, 237]. Recent data suggest that the pharmacological manipulation of cancer cell‐specific levels of ROS can provide more favorable selectivity between normal and malignant cells when other therapeutic modalities are applied, which involves reducing the level of tumor cell death associated with off‐target toxicity.

#### 5.2.1 ROS Scavengers

Targeting ROS is a successful and specific approach toward the partial reversal of some of the mechanisms underlying the acquired resistance exhibited by tumor cells to anticancer therapies. This targeting can be achieved in two ways, that is, modulation of the mechanisms responsible for ROS generation or modulation of the redox adaptative response (Figure 4), both of which represent important translational potential. However, it should be remembered that therapeutically delivered ROS modulators affect cancer cells, normal cells, and their microenvironments and that some ROS (such as H_2_O_2_) may leak from one cell and diffuse to another. Moreover, ROS generated inside cells can induce bystander reactions in nearby cells, increasing biological activity and possibly influencing tumor cell lysis [238].

However, such ROS‐mediated bystander effects could also adversely affect healthy cells. Consequently, a comprehensive assessment of the overall impact of ROS modulation, evaluated at both the tissue and organismal levels, is essential to fully understand the systemic consequences.

#### 5.2.2 Inhibition of ROS Generation

Various elements can induce ROS generation and impair redox balance in the TIME; these include not only intrinsic tumor cell features but also components of tumor‐associated stroma cells, the interplay among which ultimately influences tumor behavior. All these inputs are well supported by current reports indicating that they often lead to the activation of NOX family enzymes, which are key drivers of the pathological accumulation of ROS. As such, in this section, we devote our discussion to the design of therapies that target tumor redox balance through blocking ROS production at the source.

*Inhibition of NOX*: Oncogenic transformation, such as the upregulation of NOX expression driven by Ras, can lead to increased ROS production [239]. Studies have confirmed that NOX4 expression is significantly elevated in tumor stem cells. Preclinical experiments have shown that NOX inhibitors based on the structure of diiodo‐2‐cyclopropene acid not only effectively inhibit the in vitro proliferation of pancreatic cancer patient‐derived cells but also significantly block the growth of orthotopic transplanted tumors in vivo [240, 241]. Further mechanistic studies have revealed that NOX4 mediates ROS signaling pathways in the TME. On the basis of this pathological mechanism, targeting NOX4 through siRNA‐mediated gene silencing or the specific inhibitor GKT137831 (setanaxib) has been confirmed to effectively reverse the resistance of tumors to immune checkpoint inhibitors [242].

*Inhibition of NOS*: NOS is divided into induction type (iNOS), neural type (nNOS), and endothelial type (eNOS); among them, iNOS and eNOS are closely related to malignant tumors and are thought to be related to tumor occurrence, development, invasion and metastasis. NOS3 (eNOS) expression is significantly higher in gastric adenocarcinoma tissues than in normal tissues. In CAFs, p53 dysfunction can upregulate endothelial NOS (eNOS/NOS3) expression and promote the secretion of proinvasive chemokines. Mechanistic studies have shown that pharmacological inhibition of NOS through a three‐dimensional organotypic culture model can significantly reduce the level of procancer cytokine synthesis and effectively inhibit the invasive phenotype of oral cancer cells. These findings confirm the crucial role of the NOS signaling pathway in the regulation of the TME [243].

*Increased ROS cytotoxicity*: Owing to the pathological process of oxidative stress‐induced irreversible damage to cells under severe conditions, controlling the homeostasis of ROS metabolism pathways in tumor cells has potential as an antitumor method. Recently, it was reported that the multitarget tyrosine kinase inhibitor (TKI) anlotinib could effectively increase superoxide anion accumulation and mediate the cascade reaction of apoptosis through the upregulation of NOX5, which is a catalytic enzyme for ROS generation in vitro [57]. This drug may also demonstrate effective antitumor activity in a PDX animal model of squamous cell carcinoma [244].

#### 5.2.3 Targeting Redox Adaptation Mechanisms

Redox dysregulation is another feature of tumor cells: owing to their highly active status and dysfunctional metabolism, even a transient redox imbalance is likely to rapidly lead to tumor cell death. This redox‐sensitive state, or “redox sensitivity,” provides promising treatment opportunities to exploit impaired redox homeostasis [245, 246, 247, 248]. Tumor cells can respond to these conditions by upregulating several cellular mechanisms to counteract the resulting redox imbalance, the most important of which involve the glutathione (GSH) and thioredoxin (TXN) systems [248, 249, 250, 251, 252]. Thus, therapeutic targeting to dysregulate the GSH and TXN routes is a promising strategy to increase the efficacy of redox‐targeted approaches in clinical oncology.

*Deactivation of GSH*: Numerous in vitro data have indicated that as a plant compound with the ability to bind GSH, phenethyl isothiocyanate (PEITC) promotes the specific production of ROS and apoptosis in malignant cancer cells by lowering the levels of intracellular GSH [253, 254]. This strategy is illustrated by effective tumor selectivity, in which conventional chemotherapy is highly effective at eliminating drug‐resistant cancer cells but causes few systemic toxic effects on normal tissue cells. Notably, its analog, LBL21, can exterminate subpopulations of drug‐resistant cells with stem cell‐like properties in NSCLC. The results of animal experiments further demonstrated that following treatment with the same dose of PEITC, mice bearing mutant heterozygous oral cancer cells showed powerful inhibition of tumorigenesis; the mechanism underlying these observations could be related to reestablishing the function of p53 and blocking both GSH metabolism and the synergistic effect of ROS‐mediated cell cycle arrest [254]. Importantly, a subsequent phase II clinical trial confirmed that daily intake of Nutri‐PEITC nutritional jelly markedly increased the PFS of patients with advanced oral cancer, effectively increasing their quality of life and providing evidence for the clinical translation of phytochemicals.

*Blocking GSH peroxidase (GPX)*: Inhibiting GPX activity results in the destruction of intracellular redox homeostasis, initiating oxidative stress and leading to programmed cell death. In particular, the inhibition of GPX2 (e.g., using the TKI lenvatinib) specifically activates hepatoma apoptosis through the accumulation of ROS. Additionally, the role of GPX4 can be regulated through the small‐molecule compounds FINO2 and FIN56 to induce ferroptosis in multiple models. These findings highlight the importance of the role of the GPX family in redox regulation [255].

*Inhibition of TXN reductase (TXNRD)*: TXNRD plays a key role in the redox cycle of the TXN system. Specific inhibition of TXNRD1 by 6‐gingerol (a natural phenolic compound isolated from ginger extract) [245, 246, 256], dietary curcumin monoketone derivatives131, or N‐heterocyclic carbene complexes may lead to the overaccumulation of ROS in cells [245, 246, 256], triggering the apoptotic pathway and ultimately resulting in ferroptosis. Interestingly, preclinical studies have shown that a specific inhibitor of TXN reductase 3 (TXNRD3), cinobufagin, can successfully reverse sorafenib resistance in vitro and in vivo, providing very solid evidence supporting the efficacy of targeting TXNRD3 to treat tumors [247].

### 5.3 Targeting Lipid Metabolism

#### 5.3.1 Intervention in Fatty Acid Intake

The available evidence has shown that major lipid metabolism disorders occur in the TIME. As an important phagocytic receptor, CD36 plays a central role in long‐chain fatty acid (LCFA) absorption and low‐density lipoprotein oxidation and plays vital roles in the regulation of lipid metabolism, cell lipid intake, immune recognition, and other critical biological processes. Studies have shown that the antitumor effect of CD36‐specific inhibitors can occur through many mechanisms. These inhibitors can inhibit the progression of tumor metastasis and angiogenesis and dramatically improve the survival status and immune function of CD8^+^ T cells by increasing the antigen‐presenting ability of DCs, inhibiting PPAR signaling, decreasing the expression of Tregs, and upregulating PD‐1 expression [257]. Interestingly, they can also inhibit immune cell homeostasis through controlling lipid peroxidation reactions and ferroptosis [248]. In addition, FATPs are vital transmembrane carriers of LCFAs and facilitate fatty acid metabolism in normal tissues and malignant tumor cells. Recent studies have shown that blocking FATP2 specifically can significantly reduce the amino acid metabolism capability of MDSCs and inhibit the synthesis of PGE2, thus improving the efficacy of the immunotherapy response [249]. It has been further reported that blocking or inhibiting FATP expression can effectively increase tumor tissue lipid uptake and inhibit invasive tumor growth [250, 251] (Figure 4).

#### 5.3.2 Intervention in Fatty Acid Synthesis

Regulators of the lipid metabolism pathway, including ATP carboxylase cleavage enzyme (ACLY), acetyl‐CoA synthetase (ACSS), and fatty acid synthase (FASN), play important biological roles in tumor metabolic reprogramming. ACLY is a key enzyme that catalyzes the transformation from citrate to acetyl‐CoA, which can produce precursors for fatty acid and cholesterol synthesis and subsequently build a molecular link between carbohydrate metabolism and lipid metabolism. This enzyme and its metabolic products regulate the activation of Tregs and CD4^+^ and CD8^+^ T cells via the IL‐2 signaling cascade [252, 258]. Targeted blockade of ACLY leads to decreased intracellular fatty acid/cholesterol levels and inversely inhibits the glucose metabolism pathway [259]. Notably, the ACSS family (notably ACSS2) can synthesize acetyl‐CoA via acetate metabolic bypass, which induces drug resistance in tumor cells to ACLY inhibitors. Thus, combination therapy with ACSS2 inhibitors can effectively inhibit acetate‐dependent tumor development and increase the antitumor efficacy of ACLY inhibitors. Preclinical experiments have indicated that the expression level of ACSS2 in cervical squamous cell carcinoma is strongly positively correlated with that of PD‐L1 as well as the degree of infiltration of B cells, CD4^+^/CD8^+^ T cells, and CAFs in the TME [260], indicating that this inhibitor can achieve therapeutic effects by remodeling the immune microenvironment. FASN is the limiting enzyme that catalyzes fatty acid synthesis, and FASN inhibition promotes the assembly of lipid rafts and the activation of TLR4 signaling. However, FASN can specifically block the secretion of ROS, IL‐10, and TNF‐α (immunosuppressive factors) by M2 TAMs and block the activation of Tregs [261], indicating the multidimensional regulation of the TIME.

#### 5.3.3 Intervention in Fatty Acid Desaturation

Stearoyl‐CoA desaturase‐1 (SCD1), a key rate‐limiting enzyme that regulates the biosynthesis of monounsaturated fatty acids, plays a core metabolic role by catalyzing the conversion of saturated fatty acids to monounsaturated fatty acids within cells. SCD1‐targeted inhibitors interfere with lipid metabolic homeostasis by specifically inhibiting its catalytic activity, thereby exerting antitumor effects. These drugs not only have synergistic effects with molecular targeted therapies but also promote the antigen‐presenting function of DCs in the TME and the activation and infiltration of CD8^+^ T cells, ultimately significantly enhancing the clinical response to PD‐1 inhibitor therapy [262].

#### 5.3.4 Metabolic Signaling Blockers

Various effects of the cholesterol and oxysterol/LXR axis and SREBPs act as tumor signals. Studies have demonstrated that in HCC, the cholesterol metabolite oxysterol stimulates LXR, which, in turn, results in the upregulation of ABCA1, mediating cholesterol efflux and leading to inhibition of the secretion of proinflammatory mediators such as IL‐1β and, hence, acquired therapeutic tolerance [263]. In addition, the TTPAL–SREBP2 pathway in ESCC not only promotes cholesterol synthesis but also (through unknown mechanisms) induces PD‐L1 expression to sabotage tumor immunotherapy. The SREBP inhibitor betulinic acid stops the formation of the SREBP–SCAP complex in the nucleus and inhibits tumor growth [264].

### 5.4 Targeting Hypoxia and Angiogenesis

As detailed in Sections 2.2 (*Hypoxia*) and 3.2 (*Angiogenesis*), a self‐sustaining circuit exists between tumor hypoxia and angiogenesis: hypoxia induces HIF activation, which prompts the release of factors such as VEGF that stimulate aberrant neovascularization, resulting in the development of a leaky and tortuous vasculature and thereby further exacerbating hypoxia [137]. This self‐perpetuating pathway not only drives tumor progression but also underlies therapeutic resistance. Studies of brain metastases demonstrate a codependent relationship between HIF‐1α and VEGF, whereby these factors collaboratively facilitate tumor cell colonization and growth within the brain.

#### 5.4.1 HIF Inhibitors

HIFs play key roles in facilitating the successful growth of cancer through coordinating the transcription of genes that contribute to adaptation to hypoxia. There are two major functional isoforms: HIF‐1 and HIF‐2. In preclinical studies, inhibition of HIF‐1 and/or HIF‐2 activity led to increases in growth, angiogenesis, and metastasis in mouse models [265]. Small‐molecule inhibitors with differing degrees of HIF specificity have been described for inhibiting tumorigenic processes in vivo in mice; however, as yet, no inhibitor specific to HIF‐1 has been approved in the clinic.

Belzutifan is a specific HIF‐2α inhibitor that has recently been approved as a treatment for renal cell carcinoma. The approval of belzutifan marks the entrance of tailored targeted antiangiogenic treatment. Belzutifan is a powerful new therapy; when it is combined with a standard regimen, it has great potential to increase the survival of patients with numerous types of cancer. Dual HIF‐1 and HIF‐2 inhibitors are being actively developed. It is our expectation that these agents will emerge as important members of the cancer therapeutic arsenal, especially given their potential synergistic effects with immune checkpoint blockade [265].

#### 5.4.2 Antiangiogenesis

Traditional antiangiogenic drugs, including VEGF antibodies and VEGFR TKIs, focus on suppressing tumor neovascularization through blocking the VEGF signaling pathway. However, early clinical trials revealed shortcomings of single‐target antiangiogenic drugs in terms of acquired resistance, serious adverse events, and a short “window of opportunity” for vascular normalization [266]. In recent years, immunotherapy has provided insights into how antiangiogenic therapy can be augmented with other immunotherapeutic methods [267]. Further evidence has revealed that even at a low dose, targeting VEGF signaling can induce tumor vasculature normalization. Such an effect is mediated at least in part by blocking the polarization of TAMs toward the immunosuppressive M2‐like phenotype [268] and by reversing the VEGF‐mediated inhibition of the maturation of DCs. New evidence suggests that using bevacizumab in combination with ipilimumab is more effective [269]. In addition, antiangiogenic treatment after adoptive cell transfer [270] or cancer vaccination [268] might also facilitate TIL extravasation.

#### 5.4.3 Proangiogenic Approaches

Unlike antiangiogenic therapy, proangiogenic therapy refers to a temporal change in the tumor vascular network and function rather than the obliteration of the whole vasculature. Proangiogenic therapy represents a novel strategy to further improve existing treatments by adjusting the hypoxic and immune microenvironments. HIF prolyl hydroxylase inhibitors (HIF‐PHIs) operate by stabilizing HIF‐α and mimicking a pseudohypoxic environment in a normoxic environment to promote immune cell functional reprogramming. HIF‐PHIs induce positive effects in microsatellite‐stable colorectal cancer: HIF‐PHIs substantially upregulate the numbers of CD8^+^ and CD4^+^ TILs and decrease the percentage of Foxp3^+^ Tregs. In addition, they upregulate IL‐2 expression in the spleen and tumors through CD4^+^ T cells and increase CD8^+^ T‐cell differentiation from stem‐like cells to effector‐like cells [271].

### 5.5 Targeting the Acidic Microenvironment

#### 5.5.1 Buffers

Currently, two pH‐responsive intelligent delivery systems are under investigation. One is doxycycline (Doxy)@CaCO_3_‐PEG, which is based on nanotechnology and can achieve the precise spatiotemporal release of drugs. Specifically, Doxy@CaCO_3_‐PEG can dissolve in the acidic TME (pH 6.5) of triple‐negative breast cancer and release encapsulated Doxy, locally activating Tet‐On CAR‐T cells and ultimately completely inhibiting metastasis [272]. The second system, vitamin E succinate (VES)–diselenide bond (Se–Se)–poly‐l‐lysine (PLL), is a dual‐responsive nanocarrier with VES as the core; this core is linked by a Se–Se to PLL, and this bond degrades under conditions of low pH (6.5) and high ROS levels. This delivery system can neutralize the acidic microenvironment, induce tumor cell necroptosis, and block M2 macrophage polarization to fully suppress spontaneous metastasis in lung adenocarcinoma models [273].

In addition to such pH‐sensitive systems, many biomimetic buffering agents have been recently suggested. Inspired by nature, a new generation of microenvironment‐responsive systems known as psiL@M1M was introduced. In this system, the M1 macrophage membrane was placed on a polydopamine nanoporous membrane to encapsulate siLDHA, which downregulated LDHA, whereas TNRs immobilized on the membrane triggered the interaction with TNF‐α, inhibiting inflammation. This combination inhibits the post‐PTT inflammatory cascade as well as lactate generation in an additive fashion and seems to enhance survival in recurrent murine models in a demonstrable fashion [274].

#### 5.5.2 CAIX/XII Inhibitors

In recent years, substantial improvements have been made in the design of inhibitors that act on CA IX/XII, a key player in tumor‐specific acidification. A recently discovered benzene sulfonamide compound, reported in 2025, is characterized by a more than 10‐fold increase in inhibitory potency against CAIX over the classic inhibitor AAZ, with a *K*_i_ of 0.317 µM [275]. Independent efforts resulted in another CAIX‐targeting compound with a PROTAC‐like scaffold design that selectively triggered the lysosomal degradation of CAIX, resulting in a hypoxia‐induced sustained decrease of >90% in the protein level of CAIX [275].

### 5.6 Rational Combinations

Combination therapies involving simultaneous tumor metabolic microenvironment targeting, immune checkpoint inhibition, antiangiogenic therapy, and chemotherapy/radiotherapy can be regarded as a new therapeutic paradigm to overcome not only the difficulty of converting “cold” tumors but also therapeutic resistance, yielding more effective strategies.

*Targeting amino acid metabolism*: The glutaminase inhibitor JHU083 in combination with anti‐PD‐1 therapy was found to robustly increase the infiltration of CD8^+^ T cells (threefold increase) and the fraction of memory T cells (>50%), thereby suppressing immune evasion [276]. The inhibition of both IDO and TDO through epacadostat in combination with CTLA‐4 blockade decreases the frequency of Tregs, with a complete response achieved in 60% of the melanoma model replicates [277]. Supplementation with arginine potentiates anti‐PD‐1 efficacy (twofold improvement) in pancreatic cancer models by reprogramming memory T‐cell metabolic activity [278].

*Modulating lipid metabolism*: Neutralizing antibodies targeting CD36 inhibit T‐cell ferroptosis, increasing the anti‐PD‐1 response rate from 20 to 65% in ovarian cancer. The AKR1B1 inhibitor epalrestat significantly reverses resistance when it is combined with lenvatinib [226]. Activation of the bile acid signaling pathway using the FXR agonist obeticholic acid increases the anti‐PD‐1 response rate (2.3‐fold improvement) in HCC. This effect is mediated through the restoration of gut barrier integrity and a reduction in M1 macrophage infiltration [279].

*Vascular modulation*: Coadministration of afatinib and anlotinib extends the median PFS to 5.8 months in patients with EGFR‐TKI resistance (vs. 3.2 months for monotherapy) via a bidirectional synergistic mechanism [280].

*Combining radiotherapy/chemotherapy with immunometabolic modulation*: For recurrent glioma, an arm of steroids and immunotherapy, namely, nivolumab + ipilimumab and bevacizumab with stereotactic radiotherapy, resulted in a median OS of 15.6 months (historical control: 9 months) [281]. Enhancing immune recognition: Adoptive T‐cell therapy enhances immune surveillance by eightfold and bypasses the immunosuppressive effect of classic chemotherapy [282].

## 6 Clinical Challenges and Translational Hurdles

This chapter systematically elaborates on the core challenges of the TIME in cancer treatment and the obstacles encountered in its translational applications. First, starting from the inherent characteristics of the TIME, it highlights the functional heterogeneity and dynamic plasticity of its cellular components (such as CAFs, TAMs, and tumor‐associated neutrophils [TANs]), which leads to the failure of single‐targeted therapeutic strategies. Subsequently, a detailed analysis was conducted on how various treatment modalities (including radiotherapy, chemotherapy, targeted therapy, and immunotherapy) induce the evolution of the TIME and trigger the mechanism of drug resistance, highlighting the complexity of the interaction between therapeutic intervention and dynamic changes in the microenvironment. Furthermore, to overcome the limitations of static measurements and achieve precision medicine, the necessity of dynamically assessing biomarkers for TIME status was discussed. In addition, strategies for drug delivery tailored to specific microenvironments were assessed to enhance therapeutic efficacy; finally, the issues of therapeutic window and toxicity management were examined, emphasizing the importance of achieving a balance between efficacy and patient tolerance.

### 6.1 Heterogeneity and Dynamic Adaptation of the TIME

**FIGURE 6:**
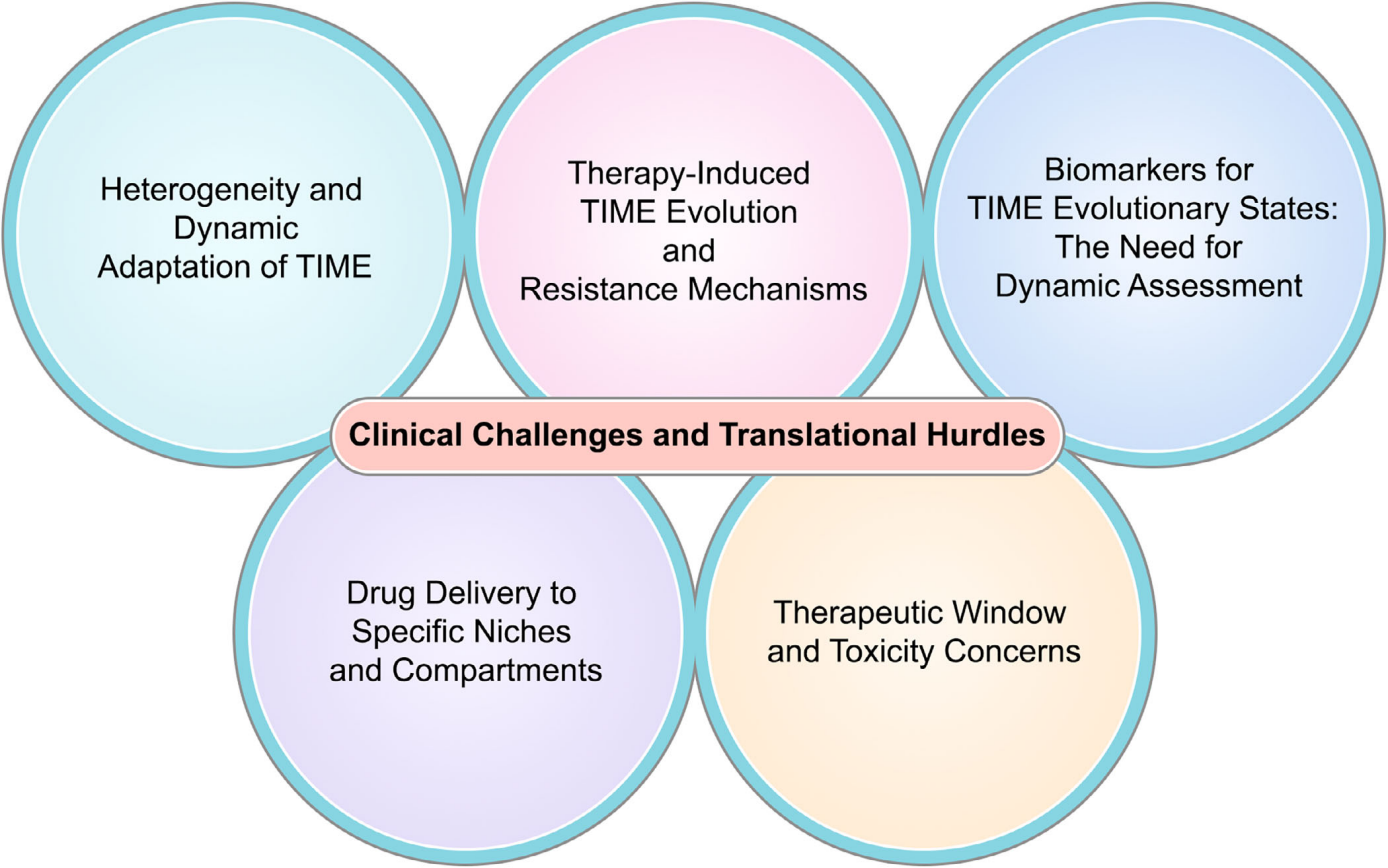
Clinical challenges and translational hurdles. The heterogeneity and dynamics of TIME determine that cancer patients face significant clinical challenges. The tumor microenvironment changes during the effective clinical treatment process, and cancer patients develop varying degrees of drug resistance. Therefore, the dynamic assessment of the evolution state of TIME has significant value and application. However, it is also accompanied by transformation obstacles such as drug delivery, toxicity risks, and treatment windows.

The involvement of multiple cellular players in the TIME makes the identification of effective therapeutic interventions extremely difficult (Figure 6). Recent studies have revealed the dual (pro‐oncogenic and oncosuppressive) roles played by important stromal components, such as CAFs, TAMs, and TANs. In pancreatic ductal adenocarcinoma (PDAC), alpha‐SMA‐positive CAFs contribute to the enrichment of CSCs. Such survival‐biased expansion fosters an increase in highly aggressive tumors, resulting in poor survival [283]. Similar functional plasticity also occurs for TAMs and TANs. For example, TAMs have potential antitumorigenic capacity, as revealed by preclinical approaches that target CD47 [284]. In contrast, pro‐oncogenic N2‐polarized TANs become protumorigenic; however, N1‐polarized TANs can be established by blocking TGFβ signaling [285]. These findings further demonstrate the natural plasticity, as well as the functional dichotomy, of CAFs, TAMs, and TANs in the TIME. These paradoxical observations are commensurate with the functional spatiotemporal heterogeneity of the TIME. This natural heterogeneity serves as one rationale for the failure to observe consistent clinical benefit with clinical trials of targeting CAFs and/or TAMs individually. Therefore, an urgent research focus should be the generation of strategies to discriminate these contrasting functions of TIME components and specifically address individual functional subpopulations. This dynamic flexibility is needed for the TIME ecosystem.

### 6.2 Therapy‐Induced TIME Evolution and Resistance Mechanisms

#### 6.2.1 Radiation Therapy

Ionizing radiation (IR) induces a chronic immunosuppressive TME, mediating therapeutic resistance [286]. IR results in the secretion of GM‐CSF, which promotes the migratory capacity of MDSCs to tumor areas and reprograms the programmed death‐ligand 1 (PD‐L1) pathway, triggering treatment resistance [287, 288]. In colorectal cancer mouse models, radiation‐induced activation of the stimulator of interferon genes (STING) pathway stimulates MDSC recruitment and subsequently suppresses CD8+ T‐cell and DC activity [289, 290, 291]. In HNSCC, radiation induces tumor cell upregulation of chemokine receptor type 2 (CCR2), resulting in the accumulation of TNFα‐producing monocytes and Tregs [292]. In ovarian cancer, macrophages associated with tumors (TAMs) release CCL22, which recruits Tregs to the margin of the tumor [293]. Esophageal cancer patients who are receiving radiation therapy overexpress the 12‐lipoxygenase (12‐LOX) gene in their tumors. The overexpression of 12‐LOX results in the polarization of THP‐1+ macrophages toward an immunosuppressive M2 macrophage phenotype through the expression of the chemokine CCL5, ultimately leading to tumor radioresistance [294].

#### 6.2.2 Chemotherapy

Evidence indicates that prolonged chemotherapy can promote chemoresistance by modulating TAMs through mechanisms such as monocyte/macrophage recruitment, depletion of monocyte/macrophage lineages, and regulation of macrophage polarization [295]. In breast cancer, paclitaxel significantly enhances TAM infiltration into tumors, and combining paclitaxel with macrophage depletion strategies improves patient survival [296]. Hughes et al. [297] demonstrated in murine models that treatment with various chemotherapeutic agents enriches the TIME with M2‐polarized TAMs, which promotes tumor recurrence. In murine models of human luminal B breast cancer, doxorubicin treatment results in the recruitment of CCR2‐expressing myeloid cells. Notably, after Ccr2 gene knockout, host mice exhibit enhanced responsiveness to doxorubicin [298].

#### 6.2.3 Targeted Therapy

Long‐term targeted therapy may mitigate treatment resistance through the recruitment of N2‐type TANs. VEGF receptor TKI (VEGFR‐TKI) treatment increases IL‐17A expression by γδ T cells. The recruitment of TANs results in N2‐type, immunosuppressive and VEGFR‐TKI‐resistant cells [299]. TAN infiltration and resistance have been observed in HCC after sorafenib treatment, and these effects are increased by the HIF‐1α/nuclear factor‐κB signaling axis [299].

#### 6.2.4 Immunotherapy

Research has indicated that the blockade of certain immune checkpoints elicits compensatory upregulation of other inhibitory checkpoints, reinitiating immunosuppression. For instance, in anti‐PD‐1 therapeutic‐resistant lung cancer murine and patient models, T cells adhering to PD‐1 antibodies exhibit elevated expression of TIM‐3 [300]. After anti‐CTLA‐4 therapy for prostate tumors, PD‐L1 and V‐domain Ig suppressor of T‐cell activation expression significantly increases on CD4^+^ T cells, CD8+ T cells and CD68+ macrophages, and PD‐L1 expression increases on tumor cells [301].

### 6.3 Biomarkers for TIME Evolutionary States: The Need for Dynamic Assessment

The TIME is not static in terms of tumorigenesis or tumor progression. Therapeutic interventions lead to dynamic changes in the TIME as tumor progression occurs [302]. As such, the dynamic TIME determines both tumor progression and response to therapy, as well as the acquisition of drug resistance. Ongoing clinical applications that adhere to static and “snapshot” measurements are highly limited in terms of not dynamically measuring and tracking the state of the TIME or treatments in real time. Hence, real‐time evaluation of TIME evolution is essential for navigating future personalized and precision treatments. Existing data show that the most extensively studied biomarkers include both intrinsic tumor cell features, such as TMB, neoantigen burden and quality, driver gene mutations, and activated oncogenic pathways, as well as features of TIME composition and dynamics, such as the infiltration profiles of T cells and myeloid cells, immune checkpoint molecule expression, immunomodulatory signaling pathways, and spatial architecture. From a clinical perspective, by combining patient‐specific biomarker profiles, customized and effective therapeutics can be developed.

### 6.4 Drug Delivery to Specific Niches and Compartments

The goal of intratumoral drug delivery is to provide continuous, high intratumoral drug concentrations with systemic toxicity. GM‐CSF has been given intratumorally in several trials [303] and has shown potential to improve melanoma antigen recognition and decrease the number of Tregs and MDSCs in the TIME [304]. Next, a phase III study was designed to compare the intratumoral administration of GM‐CSF encoding the oncolytic virus talimogene laherparepvec (T‐VEC). Compared with subcutaneous administration, intratumoral T‐VEC resulted in a better durable response rate and a trend toward longer median OS [305]. These results led to the approval of the United States Food and Drug Administration for its use in treating unresected cutaneous, subcutaneous, and nodal lesions in patients with recurrent melanoma after initial surgery [306]. Moreover, synergistic effects have been observed for conjugated immunotherapies. For example, patients with late‐stage melanoma gain a significant advantage from the combination of GM‐CSF with ipilimumab over ipilimumab alone, leading to long‐term survival with minimal toxicity [302]. Currently, a phase III trial (NCT02263508) is being conducted to assess T‐VEC combined with pembrolizumab in patients with unresectable melanoma [307].

### 6.5 Therapeutic Window and Toxicity Concerns

The therapeutic window for cancer patients in clinical determines the efficacy of cancer treatment, the quality of life of patients in the later stage, and the OS rate, and is a crucial factor in cancer treatment decisions. The patient's response to drug treatment is another crucial factor determining the effectiveness of cancer treatment, and it is also an inherent challenge for achieving individualized and timely treatment outcomes. In this context, clinical researchers need to adopt the principle of choosing the appropriate treatment strategy that not only achieves the desired therapeutic effect but also minimizes side effects as much as possible. Ultimately, they should determine the best treatment strategy for each patient's unique therapeutic window. By carefully optimizing the drug dosage, actively monitoring and managing toxic and side effects, patients can remain within the therapeutic window for as long as possible. Currently, with the continuous advancement of treatment drugs, targeted drugs, immunotherapies, and antibody–drug conjugates have provided the possibility to further expand the therapeutic window, which will help increase the clinical benefits of tumor treatment and offer more precise personalized medical care.

## 7 Future Perspectives and Emerging Frontiers

With the development of technologies such as single‐cell transcriptome sequencing and spatial transcriptome sequencing, cancer research has entered a new stage of multidisciplinary, new technologies, and multidimensional development. This chapter will systematically explore the cutting‐edge directions of future oncology research from multiple aspects, including technology integration, clinical translation, engineering intervention, new treatment strategies, and dynamic clinical trial design.

### 7.1 Deciphering TIME Evolution With Advanced Technologies

#### 7.1.1 Single‐Cell Sequencing

scRNA‐seq is promising for deciphering the genomics of individual cellular programs and allows for a more in‐depth dissection of various heterogeneous subpopulations of cells in the TIME as well as the discovery of previously uncharacterized cellular functions. For example, using scRNA‐seq, we discovered a subpopulation of CAFs defined by the expression of the protein LRRC15 (LRRC15⁺) and elevated TGF‐β signaling. This LRRC15^+^ CAF subset has been implicated as a driver of resistance to immunotherapy [308]. Antigen‐presenting CAFs, which express MHC class II molecules and can activate CD4+ T cells in an antigen‐specific manner, were similarly identified first in both murine PDAC models and human pancreatic cancer using scRNA‐seq [309]. These results suggest that unbiased scRNA‐seq data analyses can be helpful for better subpopulation classification.

#### 7.1.2 Spatial Multiomics

Spatial resolution omics platforms have made substantial progress in recent years, evolving from early single‐modality spatial transcriptomic approaches to current platforms that are able to simultaneously capture two omics modalities from a single tissue section. Such modalities can include, for instance, the combination of spatial transcriptomics with proteomics (e.g., DBiT‐seq) or spatial epigenomics (e.g., SpatialATAC‐RNA‐seq and spatial‐CUT&Tag‐RNA‐seq). More recently, Yanxiang Deng at the University of Pennsylvania established an ultrasensitive spatial multiomic method named Spatial‐Mux‐seq, which allows the simultaneous profiling of chromatin accessibility, two histone modifications, the transcriptome and the proteome, providing the first insights into multilayer gene regulatory networks [310].

#### 7.1.3 Intravital Imaging

Intravital imaging was first used in living animals for intravital pharmacokinetic screening in drug development via real‐time, noninvasive tracking of a drug. Since then, continuous fluorescence probe technology has increased the capabilities of intravital imaging. Recently, Rhobo6 technology, a fluorophore‐based approach for ultrarapid, nondestructive and stable observation of the ECM in vivo, was developed by Antonio Fiore et al. at Howard Hughes Medical Institute. This technology has high selectivity and slow photobleaching and is not restricted by the depth of tissue that can be imaged [311].

#### 7.1.4 Artificial Intelligence

Machine learning (ML) methods are being applied to help organize and interpret quantitative datasets drawn from the TME. These datasets include whole‐slide histopathological images; flow and mass cytometry data; and bulk, single‐cell, and spatial transcriptomic data [312, 313, 314]. ML‐based interpretations provide insights into the correlation between TIL profiles and additional data, including tumor DNA sequencing profiles, tumor immune profiles, tumor types, treatment effectiveness, and patient survival [315].

The convergence of scRNA‐seq, spatial transcriptomics, intravital imaging, and ML will have exciting new implications. Combining these methods can offer new ways for in vitro emulation of the human TME as well as for bridging the gap between in vivo and in vitro conditions, at least for some research purposes, and eventually even simulating in vitro conditions to compute and predict individual patient treatment outcomes.

### 7.2 Integrating TIME Evolutionary Mapping into Precision Oncology

Owing to the marked heterogeneity in the TIME between individuals, personalized medicine is rapidly becoming an important practice in oncology. Examples include the detection of certain mutational events or the overexpression of particular proteins, which can be used to define subgroups of patients who will respond to targeted treatments with maximal benefit to their particular tumor's molecular signature or patient‐derived models with a changing landscape for the preclinical evaluation of treatment responses. These models accurately simulate patient‐specific tumors and their TMEs; therefore, these systems yield more realistic predictions of treatment responses. In vitro experiments of therapeutic agents in such patient‐specific models before clinical evaluation allow the identification of promising approaches. Hence, the introduction of personalized treatment strategies has the potential to greatly increase the accuracy, effectiveness, and safety of cancer treatment, as well as lower the related health‐care costs.

### 7.3 Engineering the TIME: From Understanding to Directing Evolution

In the past, the main goal of cancer immunotherapy has been to increase the immune responses; in contrast, the current ultimate goal of cancer immunotherapy has shifted toward achieving a more fundamental understanding of the TIME such that its evolution can be actively manipulated to facilitate antitumor immunity. In the following section, we describe how our previous work has provided a systemic view of the deep complexity, dynamics, and heterogeneity of the TIME and how this view can be used to formulate corresponding therapeutic strategies. All these strategies attempt to overcome the existing immunosuppressive balance, alter the TIME to generate immune cell infiltration, increase the immune response, and promote an environment that is favorable to a persistent antitumor immune response, highlighting the need for targeted therapeutics. At present, a large number of studies have shown that a single drug treatment is no longer sufficient to address the complexity and heterogeneity of TIME. The available data strongly suggest that the application of combination therapies comprising two, three, or more components is needed to improve immunotherapy response rates and response durability. In this context, combination therapy constitutes an unavoidable paradigm. Nevertheless, this requirement poses great challenges, including the sophisticated development of stratification tools and real‐time monitoring tools; the dynamic evaluation of TIME evolution under therapeutic selective pressure to adapt the treatment strategy on time; and the more active and accurate control of drug dosing during treatment to achieve greater benefits and lower risk.

### 7.4 Novel Therapeutic Avenues

A novel therapeutic horizon based on targeted therapies and immunotherapies exists, but there are major limitations, such as low efficacy rates and toxic side effects [316]. The development of nanomedicines offers two key benefits: targeted delivery of drugs to tumor tissues and reduced toxicity to normal tissues. Several nanobodies (Nbs) have already been moved to the clinical stage over the past few years. For example, ciltacabtagene autoleucel can be used to treat multiple myeloma [317] and increase the accumulation of K2 in melanoma tumors [318, 319]. One other approach to extend the persistence of nanotherapeutics systemically is to engineer multimodal constructs. Compared with the anti‐PD‐L1 antibody avelumab, the lentivirus‐mediated delivery of the K2 Nb–Fc fusion protein improves antitumor effects in various tumor types, particularly in a three‐dimensional melanoma model [320]. Second, the humanized bispecific Nb–Fc fusion construct KN046, which targets PD‐L1/CTLA‐4, has demonstrated potent therapeutic activity in preclinical studies, and treatment with KN046 in combination with nanoparticle albumin‐bound paclitaxel (nab‐paclitaxel) is being investigated in a phase II clinical trial (NCT03872791); the results of the trial are promising for different solid tumors [321, 322].

### 7.5 Clinical Trial Design Incorporating TIME Dynamics

**FIGURE 7:**
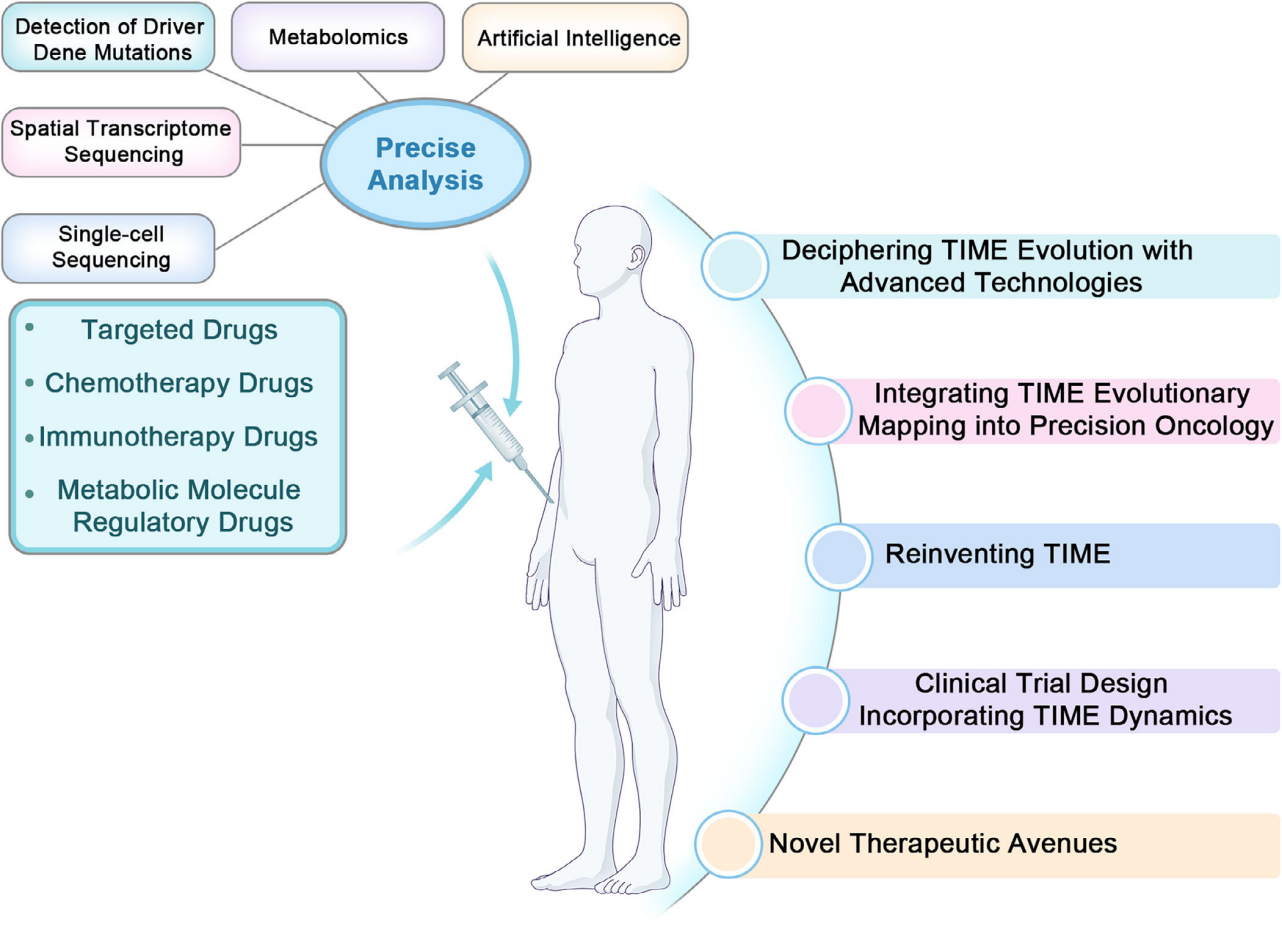
The development trend of future individualized precision treatment. By integrating information and data from single‐cell transcriptome sequencing, spatial transcriptome sequencing, and driver gene alterations through artificial intelligence data analysis technology, a TIME dynamic evolution map is obtained and incorporated into individualized precision treatment. Through clinical trials of the TIME dynamic factors, a new treatment approach for cancer patients has been proposed.

The conventional static evaluation approach to characterize the TIME and therapeutic response is insufficient to capture the temporal dynamics of the TIME and response. Clinical trials that reflect dynamic TIME elements, therefore, pose an important emerging frontier toward realigning examinations from static time points to longitudinal temporal detection. Exploiting the combination of scRNA‐seq, spatial transcriptomics, transcriptomics, proteomics, and metabolomics, researchers can now systematically investigate the pretreatment gene expression profile(s) of tumors; the mutational load, composition, and spatial distribution of TIME cell components; and dynamic changes in molecular pathways in the microenvironment to identify robust candidate biomarkers. The application of such dynamically identified biomarkers may further rationalize both the planning and design of clinical trials. Thus, protocols are needed for the dynamic monitoring of responses to therapeutic interventions through frequent longitudinal biospecimen sampling and analysis (Figure 7).

## 8 Conclusion and Future Prospects

Although therapeutic strategies targeting the TIME based on clinically approved drugs and cell therapies have been developed through significant research and exploration of the TIME over the past two decades, TIME‐related therapies still face several challenges. First, the sensitivity of each patient to immunotherapy varies. Those who respond positively to immunotherapy are called hot tumors, whereas those who do not respond are called cold tumors. The key differences between cold tumors and hot tumors in the immune microenvironment mainly lie in the composition of immune cells and the activity levels of related signaling pathways. Hot tumors are typically rich in T cells (particularly CD8⁺ T cells) and natural killer cells, accompanied by active pro‐inflammatory signals, such as activation of the STING pathway, as well as high expression of immune checkpoint molecules. Overall, they exhibit a high level of immune activity [323, 324, 325, 326, 327, 328]. In contrast, cold tumors exhibit distinct immunosuppressive characteristics: not only is there a scarcity of T‐cell infiltration in their microenvironment, but various immunosuppressive cells (such as Tregs and TAMs) as well as immunosuppressive factors (such as TGF‐β and galectin‐3) frequently accumulate, leading to the formation of an immunosuppressive or inhibitory state [329, 330, 331, 332, 333, 334, 335]. The formation of cold tumors involves mainly the following aspects: first, insufficient presentation of tumor antigens leads to ineffective activation of T cells [330, 336]; second, the absence or presence of chemokines and the presence of a stromal barrier prevent T cells from infiltrating the tumor site [330, 331]; and third, excessive activation of immunosuppressive cells and related signaling pathways occurs [329, 337]. Therefore, transforming cold tumors into hot tumors has become an important clinical strategy. The main intervention directions include enhancing immune recognition through immune priming and antigen release [338, 339, 340, 341], regulating the immunosuppressive microenvironment to weaken immunosuppressive signals [324, 326, 334, 336], and promoting the effective infiltration of immune cells such as T cells [325, 328, 329, 333, 342]. These approaches aim to reverse the “cold” phenotype of tumors and enhance the antitumor immune response.

Second, treatment for drug resistance also limits the wide application of TIME therapy. Various factors mediate immune therapy resistance. In addition to the factors mentioned in the text, intratumoral microorganisms, as components of the TIME, can also reshape the immune state of the TIME by directly infiltrating tumor cells and immune cells [343, 344]. Some studies have reported that certain microorganisms can induce the activation of immunosuppressive cells, namely, CD68^+^ SPP1^+^ macrophages, thereby inhibiting immune activity [345]. Microbial metabolites can alter the phospholipid metabolism of CD8^+^ T cells, thereby reducing their antitumor function [346]. Of course, not all intratumoral microorganisms inhibit cellular immunity. The Streptococcus genus enhances the antitumor immune response by increasing the infiltration of CD4^+^ CXCL13^+^ T cells [347], and the presence of Streptococcus in esophageal cancer can predict the response to anti‐PD‐1 therapy [348]. Moreover, the presence of *Escherichia coli* in lung cancer is associated with a proinflammatory TIME and may improve the efficacy of ICI [349]. All of these circumstances indicate that the microorganisms within tumors have great potential and value for clinical application in tumor diagnosis and treatment. However, owing to the low biomass, high heterogeneity, and technical limitations of the microorganisms within tumors, in the future, multiomics technologies need to be combined to further analyze the sources of the microorganisms, their colonization pathways, and the spatiotemporal dynamics of their interactions with tumors and surrounding cells so that they can be used more accurately for diagnosis and treatment.

Finally, the high heterogeneity of tumors remains a key obstacle to precise classification in diagnosis. For example, biomarkers such as PD‐L1 expression and the TMB are heterogeneously distributed within and between tumors, which makes it difficult for single‐point biopsy to comprehensively reflect the true status of the TIME, thus limiting therapeutic individualization. Multimodal data are the trend of clinical integration for future individualized treatment. However, in terms of technology transformation, these studies still face the dual challenges of high cost and complex operation. To effectively address these challenges, in the future, artificial intelligence can be utilized to assist in analyzing the distribution of genes, changes in metabolic pathways, accumulation of metabolites, composition of microenvironment cells, and microbial communities in the TIME of different patients. After integrating these information, an algorithm can be derived to accurately capture the dynamics and heterogeneity of TIME, thereby providing assistance for individualized precision treatment of patients. Not only that, we can also further explore the interaction networks among tumors, intratumoral microbial communities, and immune cells. While revealing new resistance mechanisms, we can also discover potential immune regulation targets.

Furthermore, most of the current clinical treatments and research on TIME are limited to the local microenvironment of tumors or certain specific types of cancers, and do not monitor the overall immune status of patients during the treatment process. Overall, there is indeed a certain bias. However, in reality, the human body is a holistic system. The immune system itself has the ability to regulate across organs and throughout the body, and it may also affect the local microenvironment of tumors. Therefore, in the future, we need to break the limitations of local biopsies or single‐cell diagnosis and treatment, and treat the human body as a whole for systematic treatment. This shift in treatment approach and concept implies that we should dynamically assess the “cancer‐host” immune regulatory interactions for different cancer types, population characteristics, and treatment stages, and understand their temporal and spatial heterogeneity as well as their dynamic evolution process.

In conclusion, the future treatment and research of TIME pose higher requirements for clinicians and researchers. They not only need to master traditional and emerging treatment strategies, but also combine the multiomics results and advanced tools such as artificial intelligence used in clinical practice to understand the dynamic evolution process of patients' TIME, and truly achieve individualized and precise treatment.

## Author Contributions

Ying Sun: Writing—review and editing, writing—original draft, visualization, and conceptualization. Changjian Shao: Writing—original draft and investigation. Hongtao Duan: Writing—original draft and data curation. Zhaoyang Wang: Writing—original draft and software. Shaopeng Xu: Visualization. Chao Wang: Investigation. Jiawei Xiu: Investigation. Jin Liu: Data curation. Xuejiao Wang: Software. Xin Yao: Visualization. Yuan Gao: Writing—review and editing and conceptualization. Xiaolong Yan: Writing—review and editing, writing—original draft, and conceptualization. All authors have read and approved the final manuscript.

## Conflicts of Interest

The authors declare no conflict of interest.

## Ethics Statement

The authors have nothing to report.

## Data Availability

The authors have nothing to report.

## References

[1] S. Tan , Y. Ming , J. Guo , et al., “A circRNA Promotes Esophageal Squamous Cell Carcinoma Progression by Inhibiting TRIM25‐mediated Degradation of IGF2BP family Members,” Molecular Cancer 24, no. 1 (2025): 243. 41044609 10.1186/s12943-025-02442-3 PMC12495880

[2] A. Indeglia , A. Valdespino , G. Pantella , et al., “Targeted Citrullination Enables p53 Binding to Non‐Canonical Sites,” Molecular Cell 85, no. 19 (2025): 3588–3604. e3511. 41043392 10.1016/j.molcel.2025.09.004 PMC12671568

[3] J. Li , T. Deng , Y. Gu , et al., “Efficacy and Safety of glecirasib in Solid Tumors With KRAS G12C Mutation: A Pooled Analysis of Two Phase I/II Trials,” Cancer Communications (London, England) (2025). 10.1002/cac2.70056 PMC12629856 41037823

[4] C. Shen , T. Cui , L. Yang , et al., “KRAS‐induced STN1 (OBFC1) Promotes Proper CTC1‐STN1‐TEN1 Complex‐Independent DNA Double‐strand Break Repair and Cell Cycle Checkpoint Maintenance in Pancreatic Cancer,” Nucleic Acids Research 53, no. 18 (2025): gkaf983. 41036624 10.1093/nar/gkaf983 PMC12489472

[5] D. Godugu , R. Chilamakuri , and S. Agarwal , “STAT3 axis in Cancer and Cancer Stem Cells: From Oncogenesis to Targeted Therapies,” Biochimica Et Biophysica Acta Reviews on Cancer (2025): 189461. 41033404 10.1016/j.bbcan.2025.189461 PMC13283684

[6] X. Huang , J. Liang , Y. Yi , et al., “Targeting Inosine Metabolism to Enhance EGFR‐targeted Therapy in Lung Adenocarcinoma,” Cancer Letters (2025): 218069. 41033606 10.1016/j.canlet.2025.218069

[7] E. S. M. van Aken , B. Devnani , A. Prelaj , et al., “ESMO‐ESTRO Consensus Statements on the Safety of Combining Radiotherapy With Immune Checkpoint Inhibitors, VEGF(R) Inhibitors, or Multitargeted Tyrosine Kinase Inhibitors,” Annals of Oncology: Official Journal of the European Society for Medical Oncology 208 (2025): 110910. 10.1016/j.annonc.2025.09.008 41016600

[8] S. Dai , Y. Chen , W. Cai , et al., “Combination Immunotherapy in Hepatocellular Carcinoma: Synergies Among Immune Checkpoints, TKIs, and Chemotherapy,” Journal of Hematology & Oncology 18, no. 1 (2025): 85. 41013723 10.1186/s13045-025-01739-6 PMC12465164

[9] X. Le , J. P. Robichaux , M. Nilsson , et al., “Poziotinib for EGFR Exon 20‐insertion NSCLC: Clinical Efficacy of the Phase 2 ZENITH Trial and Differential Impact of EGFR Exon 20 Insertion Location on Sensitivity,” Nature Communications 16, no. 1 (2025): 8358. 10.1038/s41467-025-61817-8 PMC12460621 40993146

[10] J. Ariyadamrongkwan and C. Muanprasat , “Pathophysiological Mechanisms Underlying Diarrhea Across Generations of EGFR‐TKIs: The Role of ERBB Signaling and Potential Therapies,” Biomedicine & Pharmacotherapy = Biomedecine & Pharmacotherapie 192 (2025): 118562. 40972401 10.1016/j.biopha.2025.118562

[11] B. M. Lichtenberger and M. Kasper , “Cellular Heterogeneity and Microenvironmental Control of Skin Cancer,” Journal of Internal Medicine 289, no. 5 (2021): 614–628. 32976658 10.1111/joim.13177

[12] I. Martincorena , A. Roshan , M. Gerstung , et al., “Tumor Evolution. High Burden and Pervasive Positive Selection of Somatic Mutations in Normal human Skin,” Science (New York, NY) 348, no. 6237 (2015): 880–886. 10.1126/science.aaa6806 PMC4471149 25999502

[13] K. Kuzmin , H. Schmidt , M. Kafi Kang , et al., “A Graph Homomorphism Approach for Unraveling Histories of Metastatic Cancers and Viral Outbreaks Under Evolutionary Constraints,” Nature Communications 16, no. 1 (2025): 8027. 10.1038/s41467-025-63411-4 PMC12394457 40877260

[14] P. Anand , A. B. Kunnumakkara , C. Sundaram , et al., “Cancer Is a Preventable Disease That Requires Major Lifestyle Changes,” Pharmaceutical Research 25, no. 9 (2008): 2097–2116. 18626751 10.1007/s11095-008-9661-9 PMC2515569

[15] F. Tang , C. Barbacioru , Y. Wang , et al., “mRNA‐Seq Whole‐transcriptome Analysis of a Single Cell,” Nature Methods 6, no. 5 (2009): 377–382. 19349980 10.1038/nmeth.1315

[16] N. Navin , J. Kendall , J. Troge , et al., “Tumour Evolution Inferred by Single‐cell Sequencing,” Nature 472, no. 7341 (2011): 90–94. 21399628 10.1038/nature09807 PMC4504184

[17] D. A. Jaitin , E. Kenigsberg , H. Keren‐Shaul , et al., “Massively Parallel Single‐cell RNA‐seq for Marker‐free Decomposition of Tissues Into Cell Types,” Science (New York, NY) 343, no. 6172 (2014): 776–779. 10.1126/science.1247651 PMC4412462 24531970

[18] A. P. Patel , I. Tirosh , J. J. Trombetta , et al., “Single‐cell RNA‐seq Highlights Intratumoral Heterogeneity in Primary Glioblastoma,” Science (New York, NY) 344, no. 6190 (2014): 1396–1401. 10.1126/science.1254257 PMC4123637 24925914

[19] Y. Wang , B. Liu , Q. Min , et al., “Spatial Transcriptomics Delineates Molecular Features and Cellular Plasticity in Lung Adenocarcinoma Progression,” Cell Discovery 9, no. 1 (2023): 96. 37723144 10.1038/s41421-023-00591-7 PMC10507052

[20] F. Wang , J. Long , L. Li , et al., “Single‐cell and Spatial Transcriptome Analysis Reveals the Cellular Heterogeneity of Liver Metastatic Colorectal Cancer,” Science Advances 9, no. 24 (2023): eadf5464. 37327339 10.1126/sciadv.adf5464 PMC10275599

[21] C. Ma , C. Yang , A. Peng , et al., “Pan‐cancer Spatially Resolved Single‐cell Analysis Reveals the Crosstalk Between Cancer‐associated Fibroblasts and Tumor Microenvironment,” Molecular Cancer 22, no. 1 (2023): 170. 37833788 10.1186/s12943-023-01876-x PMC10571470

[22] T. Wu and Y. Dai , “Tumor Microenvironment and Therapeutic Response,” Cancer Letters 387 (2017): 61–68. 26845449 10.1016/j.canlet.2016.01.043

[23] D. Hanahan and L. M. Coussens , “Accessories to the Crime: Functions of Cells Recruited to the Tumor Microenvironment,” Cancer Cell 21, no. 3 (2012): 309–322. 22439926 10.1016/j.ccr.2012.02.022

[24] M. Malvicini , M. Ingolotti , F. Piccioni , et al., “Reversal of Gastrointestinal Carcinoma‐induced Immunosuppression and Induction of Antitumoural Immunity by a Combination of Cyclophosphamide and Gene Transfer of IL‐12,” Molecular Oncology 5, no. 3 (2011): 242–255. 21515097 10.1016/j.molonc.2011.03.007 PMC5528288

[25] X. Chu , W. Tian , Z. Wang , et al., “Co‐inhibition of TIGIT and PD‐1/PD‐L1 in Cancer Immunotherapy: Mechanisms and Clinical Trials,” Molecular Cancer 22, no. 1 (2023): 93. 37291608 10.1186/s12943-023-01800-3 PMC10249258

[26] F. Wu , J. Yang , J. Liu , et al., “Signaling Pathways in Cancer‐associated Fibroblasts and Targeted Therapy for Cancer,” Signal Transduction and Targeted Therapy 6, no. 1 (2021): 218. 34108441 10.1038/s41392-021-00641-0 PMC8190181

[27] Y. Zhang , Y. Yang , J. Shi , et al., “A Multimodal Strategy of Fe(3)O(4)@ZIF‐8/GOx@MnO(2) Hybrid Nanozyme via TME Modulation for Tumor Therapy,” Nanoscale 13, no. 39 (2021): 16571–16588. 34585187 10.1039/d1nr04196g

[28] J. F. Gainor , A. T. Shaw , L. V. Sequist , et al., “EGFR Mutations and ALK Rearrangements Are Associated With Low Response Rates to PD‐1 Pathway Blockade in Non‐Small Cell Lung Cancer: A Retrospective Analysis,” Clinical Cancer Research: an Official Journal of the American Association for Cancer Research 22, no. 18 (2016): 4585–4593. 27225694 10.1158/1078-0432.CCR-15-3101 PMC5026567

[29] C. K. Lee , J. Man , S. Lord , et al., “Checkpoint Inhibitors in Metastatic EGFR‐Mutated Non‐Small Cell Lung Cancer‐A Meta‐Analysis,” Journal of Thoracic Oncology: Official Publication of the International Association for the Study of Lung Cancer 12, no. 2 (2017): 403–407. 27765535 10.1016/j.jtho.2016.10.007

[30] H. J. Jun , S. Woolfenden , S. Coven , et al., “Epigenetic Regulation of c‐ROS Receptor Tyrosine Kinase Expression in Malignant Gliomas,” Cancer Research 69, no. 6 (2009): 2180–2184. 19276365 10.1158/0008-5472.CAN-08-3351

[31] C. H. Shih , Y. J. Chang , W. C. Huang , et al., “EZH2‐mediated Upregulation of ROS1 Oncogene Promotes Oral Cancer Metastasis,” Oncogene 36, no. 47 (2017): 6542–6554. 28759046 10.1038/onc.2017.262 PMC5702718

[32] G. Villacampa , T. Pascual , P. Tarantino , et al., “HER2DX and Survival Outcomes in Early‐stage HER2‐positive Breast Cancer: An Individual Patient‐level Meta‐analysis,” The Lancet Oncology 26, no. 8 (2025): 1100–1112. 40675165 10.1016/S1470-2045(25)00276-1

[33] P. Trillo Aliaga , G. Spitaleri , I. Attili , et al., “HER2 in Non‐Small Cell Lung Cancer (NSCLC): Evolution of the Therapeutic Landscape and Emerging Drugs‐A Long Way to the Top,” Molecules (Basel, Switzerland) 30, no. 12 (2025): 2645. 40572608 10.3390/molecules30122645 PMC12195848

[34] F. Ciciriello , G. Pezzicoli , A. Biasi , et al., “HER2‐Positive Urothelial Carcinoma: Current Evidence on Targeted Agents and Immunotherapy‐Based Combinations,” Targeted Oncology 20, no. 5 (2025): 755–765. 40608208 10.1007/s11523-025-01165-1

[35] J. S. Thompson , L. Madrid , B. Hernando , et al., “Predicting Resistance to Chemotherapy Using Chromosomal Instability Signatures,” Nature Genetics 57, no. 7 (2025): 1708–1717. 40551015 10.1038/s41588-025-02233-y PMC12283407

[36] L. Li , L. Borodyansky , and Y. Yang , “Genomic Instability en Route to and From Cancer Stem Cells,” Cell Cycle (Georgetown, Tex) 8, no. 7 (2009): 1000–1002. 19270518 10.4161/cc.8.7.8041

[37] D. Singh , “Advancing Precision Oncology: Overcoming Treatment Resistance for Personalized Cancer Care,” Current Molecular Medicine 25, no. 7 (2025): 779–781. 40077818 10.2174/0115665240375997250312050425

[38] C. Huang , X. Xin , X. Hao , et al., “Construction of a Whole‐brain Panorama for Glioma Vasculature Reveals Tumor Heterogeneity and Blood‐brain Barrier Disruption,” Science Advances 11, no. 30 (2025): eadw8330. 40700511 10.1126/sciadv.adw8330 PMC12285729

[39] N. McGranahan , A. J. Furness , R. Rosenthal , et al., “Clonal Neoantigens Elicit T Cell Immunoreactivity and Sensitivity to Immune Checkpoint Blockade,” Science (New York, NY) 351, no. 6280 (2016): 1463–1469. 10.1126/science.aaf1490 PMC4984254 26940869

[40] Y. Wolf , O. Bartok , S. Patkar , et al., “UVB‐Induced Tumor Heterogeneity Diminishes Immune Response in Melanoma,” Cell 179, no. 1 (2019): 219–235. e221. 31522890 10.1016/j.cell.2019.08.032 PMC6863386

[41] H. Warren and J. Olsburgh , “Management of Renal Cell Carcinoma and Other Renal Masses in the Kidney Graft,” Current Urology Reports 21, no. 1 (2020): 8. 32048068 10.1007/s11934-020-0959-4

[42] R. Rosenthal , E. L. Cadieux , R. Salgado , et al., “Neoantigen‐directed Immune Escape in Lung Cancer Evolution,” Nature 567, no. 7749 (2019): 479–485. 30894752 10.1038/s41586-019-1032-7 PMC6954100

[43] E. K. Sloan , S. J. Priceman , B. F. Cox , et al., “The Sympathetic Nervous System Induces a Metastatic Switch in Primary Breast Cancer,” Cancer Research 70, no. 18 (2010): 7042–7052. 20823155 10.1158/0008-5472.CAN-10-0522 PMC2940980

[44] L. Schito and G. L. Semenza , “Hypoxia‐Inducible Factors: Master Regulators of Cancer Progression,” Trends in Cancer 2, no. 12 (2016): 758–770. 28741521 10.1016/j.trecan.2016.10.016

[45] G. L. Semenza and G. L. Wang , “A Nuclear Factor Induced by Hypoxia via De Novo Protein Synthesis Binds to the human Erythropoietin Gene Enhancer at a Site Required for Transcriptional Activation,” Molecular and Cellular Biology 12, no. 12 (1992): 5447–5454. 1448077 10.1128/mcb.12.12.5447-5454.1992 PMC360482

[46] A. Palazon , P. A. Tyrakis , D. Macias , et al., “An HIF‐1α/VEGF‐A Axis in Cytotoxic T Cells Regulates Tumor Progression,” Cancer Cell 32, no. 5 (2017): 669–683. e665. 29136509 10.1016/j.ccell.2017.10.003 PMC5691891

[47] D. Ribatti , “Tumor Refractoriness to anti‐VEGF therapy,” Oncotarget 7, no. 29 (2016): 46668–46677. 27081695 10.18632/oncotarget.8694 PMC5216828

[48] D. Fukumura , L. Xu , Y. Chen , et al., “Hypoxia and Acidosis Independently Up‐regulate Vascular Endothelial Growth Factor Transcription in Brain Tumors in Vivo,” Cancer Research 61, no. 16 (2001): 6020–6024. 11507045

[49] F. M. Buffa , A. L. Harris , C. M. West , et al., “Large Meta‐analysis of Multiple Cancers Reveals a Common, Compact and Highly Prognostic Hypoxia Metagene,” British Journal of Cancer 102, no. 2 (2010): 428–435. 20087356 10.1038/sj.bjc.6605450 PMC2816644

[50] V. Bhandari , C. H. Li , R. G. Bristow , et al., “Divergent Mutational Processes Distinguish Hypoxic and Normoxic Tumours,” Nature Communications 11, no. 1 (2020): 737. 10.1038/s41467-019-14052-x PMC7002770 32024819

[51] S. Ye , M. Lee , D. Lee , et al., “Association of Long‐term Use of Low‐Dose Aspirin as Chemoprevention with Risk of Lung Cancer,” JAMA Network Open 2, no. 3 (2019): e190185. 30821825 10.1001/jamanetworkopen.2019.0185 PMC6484667

[52] L. Ma , M. O. Hernandez , Y. Zhao , et al., “Tumor Cell Biodiversity Drives Microenvironmental Reprogramming in Liver Cancer,” Cancer Cell 36, no. 4 (2019): 418–430. e416. 31588021 10.1016/j.ccell.2019.08.007 PMC6801104

[53] R. J. DeBerardinis and N. S. Chandel , “Fundamentals of Cancer Metabolism,” Science Advances 2, no. 5 (2016): e1600200. 27386546 10.1126/sciadv.1600200 PMC4928883

[54] N. N. Pavlova and C. B. Thompson , “The Emerging Hallmarks of Cancer Metabolism,” Cell Metabolism 23, no. 1 (2016): 27–47. 26771115 10.1016/j.cmet.2015.12.006 PMC4715268

[55] K. M. McAndrews , D. J. McGrail , N. Ravikumar , et al., “Mesenchymal Stem Cells Induce Directional Migration of Invasive Breast Cancer Cells Through TGF‐β,” Scientific Reports 5 (2015): 16941. 26585689 10.1038/srep16941 PMC4653660

[56] P. Y. Lam , “Biological Effects of Cancer‐secreted Factors on human Mesenchymal Stem Cells,” Stem Cell Research & Therapy 4, no. 6 (2013): 138. 24456712 10.1186/scrt349 PMC4056665

[57] H. R. Hofer and R. S. Tuan , “Secreted Trophic Factors of Mesenchymal Stem Cells Support Neurovascular and Musculoskeletal Therapies,” Stem Cell Research & Therapy 7, no. 1 (2016): 131. 27612948 10.1186/s13287-016-0394-0 PMC5016979

[58] V. Plaks , N. Kong , and Z. Werb , “The Cancer Stem Cell Niche: How Essential Is the Niche in Regulating Stemness of Tumor Cells?,” Cell Stem Cell 16, no. 3 (2015): 225–238. 25748930 10.1016/j.stem.2015.02.015 PMC4355577

[59] C. D. Mills , L. L. Lenz , and R. A. Harris , “A Breakthrough: Macrophage‐Directed Cancer Immunotherapy,” Cancer Research 76, no. 3 (2016): 513–516. 26772756 10.1158/0008-5472.CAN-15-1737 PMC4738030

[60] T. Kato , K. Noma , T. Ohara , et al., “Cancer‐Associated Fibroblasts Affect Intratumoral CD8(+) and FoxP3(+) T Cells via IL6 in the Tumor Microenvironment,” Clinical Cancer Research: an Official Journal of the American Association for Cancer Research 24, no. 19 (2018): 4820–4833. 29921731 10.1158/1078-0432.CCR-18-0205

[61] J. T. Cheng , Y. N. Deng , H. M. Yi , et al., “Hepatic Carcinoma‐associated Fibroblasts Induce IDO‐producing Regulatory Dendritic Cells Through IL‐6‐mediated STAT3 Activation,” Oncogenesis 5, no. 2 (2016): e198. 26900950 10.1038/oncsis.2016.7 PMC5154347

[62] Q. Zhao , L. Huang , G. Qin , et al., “Cancer‐associated Fibroblasts Induce Monocytic Myeloid‐derived Suppressor Cell Generation via IL‐6/Exosomal miR‐21‐activated STAT3 Signaling to Promote Cisplatin Resistance in Esophageal Squamous Cell Carcinoma,” Cancer Letters 518 (2021): 35–48. 34139285 10.1016/j.canlet.2021.06.009

[63] L. Gorchs , C. Fernández Moro , P. Bankhead , et al., “Human Pancreatic Carcinoma‐Associated Fibroblasts Promote Expression of Co‐inhibitory Markers on CD4(+) and CD8(+) T‐Cells,” Frontiers in Immunology 10 (2019): 847. 31068935 10.3389/fimmu.2019.00847 PMC6491453

[64] D. Goehrig , J. Nigri , R. Samain , et al., “Stromal Protein Βig‐h3 Reprogrammes Tumour Microenvironment in Pancreatic Cancer,” Gut 68, no. 4 (2019): 693–707. 30415234 10.1136/gutjnl-2018-317570 PMC6580775

[65] M. Balsamo , F. Scordamaglia , G. Pietra , et al., “Melanoma‐associated Fibroblasts Modulate NK Cell Phenotype and Antitumor Cytotoxicity,” Proceedings of the National Academy of Sciences of the United States of America 106, no. 49 (2009): 20847–20852. 19934056 10.1073/pnas.0906481106 PMC2791633

[66] T. Li , Y. Yang , X. Hua , et al., “Hepatocellular Carcinoma‐associated Fibroblasts Trigger NK Cell Dysfunction via PGE2 and IDO,” Cancer Letters 318, no. 2 (2012): 154–161. 22182446 10.1016/j.canlet.2011.12.020

[67] S. Gao , T. W. Hsu , and L. I. MO , “Immunity Beyond Cancer Cells: Perspective From Tumor Tissue,” Trends in Cancer 7, no. 11 (2021): 1010–1019. 34305041 10.1016/j.trecan.2021.06.007 PMC8541902

[68] C. Laurent , S. Dietrich , and K. Tarte , “Cell Cross Talk Within the Lymphoma Tumor Microenvironment: Follicular Lymphoma as a Paradigm,” Blood 143, no. 12 (2024): 1080–1090. 38096368 10.1182/blood.2023021000

[69] S. E. Weis‐Banke , T. L. Lisle , M. Perez‐Penco , et al., “Arginase‐2‐specific Cytotoxic T Cells Specifically Recognize Functional Regulatory T Cells,” Journal for Immunotherapy of Cancer 10, no. 10 (2022): e005326. 36316062 10.1136/jitc-2022-005326 PMC9628693

[70] R. Ringquist , D. Ghoshal , R. Jain , et al., “Understanding and Improving Cellular Immunotherapies Against Cancer: From Cell‐manufacturing to Tumor‐immune Models,” Advanced Drug Delivery Reviews 179 (2021): 114003. 34653533 10.1016/j.addr.2021.114003

[71] L. Cassetta , S. Fragkogianni , A. H. Sims , et al., “Human Tumor‐Associated Macrophage and Monocyte Transcriptional Landscapes Reveal Cancer‐Specific Reprogramming, Biomarkers, and Therapeutic Targets,” Cancer Cell 35, no. 4 (2019): 588–602. e510. 30930117 10.1016/j.ccell.2019.02.009 PMC6472943

[72] S. Chevrier , J. H. Levine , V. R. T. Zanotelli , et al., “An Immune Atlas of Clear Cell Renal Cell Carcinoma,” Cell 169, no. 4 (2017): 736–749. e718. 28475899 10.1016/j.cell.2017.04.016 PMC5422211

[73] A. J. Gentles , A. M. Newman , C. L. Liu , et al., “The Prognostic Landscape of Genes and Infiltrating Immune Cells Across human Cancers,” Nature Medicine 21, no. 8 (2015): 938–945. 10.1038/nm.3909 PMC4852857 26193342

[74] J. Wagner , M. A. Rapsomaniki , S. Chevrier , et al., “A Single‐Cell Atlas of the Tumor and Immune Ecosystem of Human Breast Cancer,” Cell 177, no. 5 (2019): 1330–1345. e1318. 30982598 10.1016/j.cell.2019.03.005 PMC6526772

[75] M. F. Cuccarese , J. M. Dubach , C. Pfirschke , et al., “Heterogeneity of Macrophage Infiltration and Therapeutic Response in Lung Carcinoma Revealed by 3D Organ Imaging,” Nature Communications 8 (2017): 14293. 10.1038/ncomms14293 PMC5309815 28176769

[76] Y. Lavin , S. Kobayashi , A. Leader , et al., “Innate Immune Landscape in Early Lung Adenocarcinoma by Paired Single‐Cell Analyses,” Cell 169, no. 4 (2017): 750–765. e717. 28475900 10.1016/j.cell.2017.04.014 PMC5737939

[77] L. Cassetta and J. W. Pollard , “Targeting Macrophages: Therapeutic Approaches in Cancer,” Nature Reviews Drug Discovery 17, no. 12 (2018): 887–904. 30361552 10.1038/nrd.2018.169

[78] C. D. Mills , K. Kincaid , J. M. Alt , et al., “M‐1/M‐2 Macrophages and the Th1/Th2 Paradigm,” Journal of Immunology (Baltimore, Md: 1950) 164, no. 12 (2000): 6166–6173. 10843666 10.4049/jimmunol.164.12.6166

[79] A. Mantovani , F. Marchesi , A. Malesci , et al., “Tumour‐associated Macrophages as Treatment Targets in Oncology,” Nature Reviews Clinical Oncology 14, no. 7 (2017): 399–416. 10.1038/nrclinonc.2016.217 PMC5480600 28117416

[80] M. Wenes , M. Shang , M. Di Matteo , et al., “Macrophage Metabolism Controls Tumor Blood Vessel Morphogenesis and Metastasis,” Cell Metabolism 24, no. 5 (2016): 701–715. 27773694 10.1016/j.cmet.2016.09.008

[81] B. Ruffell and L. M. Coussens , “Macrophages and Therapeutic Resistance in Cancer,” Cancer Cell 27, no. 4 (2015): 462–472. 25858805 10.1016/j.ccell.2015.02.015 PMC4400235

[82] S. B. Coffelt , A. O. Tal , A. Scholz , et al., “Angiopoietin‐2 Regulates Gene Expression in TIE2‐expressing Monocytes and Augments Their Inherent Proangiogenic Functions,” Cancer Research 70, no. 13 (2010): 5270–5280. 20530679 10.1158/0008-5472.CAN-10-0012

[83] D. Laoui , E. Van Overmeire , G. Di Conza , et al., “Tumor Hypoxia Does Not Drive Differentiation of Tumor‐associated Macrophages but Rather Fine‐tunes the M2‐Like Macrophage Population,” Cancer Research 74, no. 1 (2014): 24–30. 24220244 10.1158/0008-5472.CAN-13-1196

[84] J. Forssell , A. Oberg , M. L. Henriksson , et al., “High Macrophage Infiltration Along the Tumor front Correlates With Improved Survival in Colon Cancer,” Clinical Cancer Research: an Official Journal of the American Association for Cancer Research 13, no. 5 (2007): 1472–1479. 17332291 10.1158/1078-0432.CCR-06-2073

[85] M. Yang , D. McKay , J. W. Pollard , et al., “Diverse Functions of Macrophages in Different Tumor Microenvironments,” Cancer Research 78, no. 19 (2018): 5492–5503. 30206177 10.1158/0008-5472.CAN-18-1367 PMC6171744

[86] P. X. Liew and P. Kubes , “The Neutrophil's Role during Health and Disease,” Physiological Reviews 99, no. 2 (2019): 1223–1248. 30758246 10.1152/physrev.00012.2018

[87] Z. G. Fridlender , J. Sun , S. Kim , et al., “Polarization of Tumor‐associated Neutrophil Phenotype by TGF‐beta: “N1” versus “N2” TAN,” Cancer Cell 16, no. 3 (2009): 183–194. 19732719 10.1016/j.ccr.2009.06.017 PMC2754404

[88] D. Bausch , T. Pausch , T. Krauss , et al., “Neutrophil Granulocyte Derived MMP‐9 Is a VEGF Independent Functional Component of the Angiogenic Switch in Pancreatic Ductal Adenocarcinoma,” Angiogenesis 14, no. 3 (2011): 235–243. 21442180 10.1007/s10456-011-9207-3 PMC3688040

[89] S. L. Zhou , Z. J. Zhou , Z. Q. Hu , et al., “Tumor‐Associated Neutrophils Recruit Macrophages and T‐Regulatory Cells to Promote Progression of Hepatocellular Carcinoma and Resistance to Sorafenib,” Gastroenterology 150, no. 7 (2016): 1646–1658. e1617. 26924089 10.1053/j.gastro.2016.02.040

[90] M. Yang , G. Zhang , Y. Wang , et al., “Tumour‐associated Neutrophils Orchestrate Intratumoural IL‐8‐driven Immune Evasion Through Jagged2 Activation in Ovarian Cancer,” British Journal of Cancer 123, no. 9 (2020): 1404–1416. 32778818 10.1038/s41416-020-1026-0 PMC7591527

[91] E. Charafe‐Jauffret , C. Ginestier , F. Iovino , et al., “Breast Cancer Cell Lines Contain Functional Cancer Stem Cells With Metastatic Capacity and a Distinct Molecular Signature,” Cancer Research 69, no. 4 (2009): 1302–1313. 19190339 10.1158/0008-5472.CAN-08-2741 PMC2819227

[92] M. Shimizu and N. Tanaka , “IL‐8‐induced O‐GlcNAc Modification via GLUT3 and GFAT Regulates Cancer Stem Cell‐Like Properties in Colon and Lung Cancer Cells,” Oncogene 38, no. 9 (2019): 1520–1533. 30305725 10.1038/s41388-018-0533-4

[93] D. Cao , H. Xu , X. Xu , et al., “High Tumor Mutation Burden Predicts Better Efficacy of Immunotherapy: A Pooled Analysis of 103078 Cancer Patients,” Oncoimmunology 8, no. 9 (2019): e1629258. 31428527 10.1080/2162402X.2019.1629258 PMC6685508

[94] B. R. Ford , P. D. A. Vignali , N. L. Rittenhouse , et al., “Tumor Microenvironmental Signals Reshape Chromatin Landscapes to Limit the Functional Potential of Exhausted T Cells,” Science Immunology 7, no. 74 (2022): eabj9123. 35930654 10.1126/sciimmunol.abj9123 PMC9851604

[95] J. Galon and D. Bruni , “Approaches to Treat Immune Hot, Altered and Cold Tumours With Combination Immunotherapies,” Nature Reviews Drug Discovery 18, no. 3 (2019): 197–218. 30610226 10.1038/s41573-018-0007-y

[96] L. Xiao , H. Yeung , M. Haber , et al., “Immunometabolism: A ‘Hot’ Switch for ‘Cold’ Pediatric Solid Tumors,” Trends in Cancer 7, no. 8 (2021): 751–777. 34183305 10.1016/j.trecan.2021.05.002

[97] H. E. Ghoneim , Y. Fan , A. Moustaki , et al., “De Novo Epigenetic Programs Inhibit PD‐1 Blockade‐Mediated T Cell Rejuvenation,” Cell 170, no. 1 (2017): 142–157. e119. 28648661 10.1016/j.cell.2017.06.007 PMC5568784

[98] S. K. Vodnala , R. Eil , R. J. Kishton , et al., “T Cell Stemness and Dysfunction in Tumors Are Triggered by a Common Mechanism,” Science (New York, NY) 363, no. 6434 (2019): eaau0135. 10.1126/science.aau0135 PMC8194369 30923193

[99] R. Eil , S. K. Vodnala , D. Clever , et al., “Ionic Immune Suppression Within the Tumour Microenvironment Limits T Cell Effector Function,” Nature 537, no. 7621 (2016): 539–543. 27626381 10.1038/nature19364 PMC5204372

[100] J. Song , X. Yi , R. Gao , et al., “Impact of Drp1‐Mediated Mitochondrial Dynamics on T Cell Immune Modulation,” Frontiers in Immunology 13 (2022): 873834. 35432303 10.3389/fimmu.2022.873834 PMC9008543

[101] J. Pu , Z. Xu , J. Nian , et al., “M2 macrophage‐derived Extracellular Vesicles Facilitate CD8+T Cell Exhaustion in Hepatocellular Carcinoma via the miR‐21‐5p/YOD1/YAP/β‐catenin Pathway,” Cell Death Discovery 7, no. 1 (2021): 182. 34282135 10.1038/s41420-021-00556-3 PMC8289864

[102] C. Kaltenmeier , H. O. Yazdani , K. Morder , et al., “Neutrophil Extracellular Traps Promote T Cell Exhaustion in the Tumor Microenvironment,” Frontiers in Immunology 12 (2021): 785222. 34899751 10.3389/fimmu.2021.785222 PMC8652262

[103] S. Sakaguchi , T. Yamaguchi , T. Nomura , et al., “Regulatory T Cells and Immune Tolerance,” Cell 133, no. 5 (2008): 775–787. 18510923 10.1016/j.cell.2008.05.009

[104] D. Shevyrev and V. Tereshchenko , “Treg Heterogeneity, Function, and Homeostasis,” Frontiers in Immunology 10 (2019): 3100. 31993063 10.3389/fimmu.2019.03100 PMC6971100

[105] S. Sakaguchi , M. Miyara , C. M. Costantino , et al., “FOXP3+ regulatory T Cells in the human Immune System,” Nature Reviews Immunology 10, no. 7 (2010): 490–500. 10.1038/nri2785 20559327

[106] J. Huehn and M. Beyer , “Epigenetic and Transcriptional Control of Foxp3+ Regulatory T Cells,” Seminars in Immunology 27, no. 1 (2015): 10–18. 25801206 10.1016/j.smim.2015.02.002

[107] M. Ono , “Control of Regulatory T‐cell Differentiation and Function by T‐cell Receptor Signalling and Foxp3 Transcription Factor Complexes,” Immunology 160, no. 1 (2020): 24–37. 32022254 10.1111/imm.13178 PMC7160660

[108] Y. You , Y. Li , M. Li , et al., “Ovarian Cancer Stem Cells Promote Tumour Immune Privilege and Invasion via CCL5 and Regulatory T Cells,” Clinical and Experimental Immunology 191, no. 1 (2018): 60–73. 28868628 10.1111/cei.13044 PMC5721255

[109] F. Wang , L. Peng , Y. Wang , et al., “A Meta‐Analysis of Vascular Endothelial Growth Factor for Nasopharyngeal Cancer Prognosis,” Frontiers in Oncology 8 (2018): 486. 30430078 10.3389/fonc.2018.00486 PMC6220117

[110] S. J. Schoenleber , D. M. Kurtz , J. A. Talwalkar , et al., “Prognostic Role of Vascular Endothelial Growth Factor in Hepatocellular Carcinoma: Systematic Review and Meta‐analysis,” British Journal of Cancer 100, no. 9 (2009): 1385–1392. 19401698 10.1038/sj.bjc.6605017 PMC2694418

[111] M. Inoue , J. H. Hager , N. Ferrara , et al., “VEGF‐A Has a Critical, Nonredundant Role in Angiogenic Switching and Pancreatic Beta Cell Carcinogenesis,” Cancer Cell 1, no. 2 (2002): 193–202. 12086877 10.1016/s1535-6108(02)00031-4

[112] S. S. Oladipupo , A. U. Kabir , C. Smith , et al., “Impaired Tumor Growth and Angiogenesis in Mice Heterozygous for Vegfr2 (Flk1),” Scientific Reports 8, no. 1 (2018): 14724. 30283071 10.1038/s41598-018-33037-2 PMC6170482

[113] R. Lugano , M. Ramachandran , and A. Dimberg , “Tumor Angiogenesis: Causes, Consequences, Challenges and Opportunities,” Cellular and Molecular Life Sciences: CMLS 77, no. 9 (2020): 1745–1770. 31690961 10.1007/s00018-019-03351-7 PMC7190605

[114] M. De Palma , D. Biziato , and T. V. Petrova , “Microenvironmental Regulation of Tumour Angiogenesis,” Nature Reviews Cancer 17, no. 8 (2017): 457–474. 28706266 10.1038/nrc.2017.51

[115] J. Li , D. Wang , F. Tang , et al., “Pan‐cancer Integrative Analyses Dissect the Remodeling of Endothelial Cells in human Cancers,” National Science Review 11, no. 9 (2024): nwae231. 39345334 10.1093/nsr/nwae231 PMC11429526

[116] F. Hossain , A. A. Al‐Khami , D. Wyczechowska , et al., “Inhibition of Fatty Acid Oxidation Modulates Immunosuppressive Functions of Myeloid‐Derived Suppressor Cells and Enhances Cancer Therapies,” Cancer Immunology Research 3, no. 11 (2015): 1236–1247. 26025381 10.1158/2326-6066.CIR-15-0036 PMC4636942

[117] S. K. Biswas , P. Allavena , and A. Mantovani , “Tumor‐associated Macrophages: Functional Diversity, Clinical Significance, and Open Questions,” Seminars in Immunopathology 35, no. 5 (2013): 585–600. 23657835 10.1007/s00281-013-0367-7

[118] P. Chen , Y. Huang , R. Bong , et al., “Tumor‐associated Macrophages Promote Angiogenesis and Melanoma Growth via Adrenomedullin in a Paracrine and Autocrine Manner,” Clinical Cancer Research: an Official Journal of the American Association for Cancer Research 17, no. 23 (2011): 7230–7239. 21994414 10.1158/1078-0432.CCR-11-1354

[119] T. Condamine , I. Ramachandran , J. I. Youn , et al., “Regulation of Tumor Metastasis by Myeloid‐derived Suppressor Cells,” Annual Review of Medicine 66 (2015): 97–110. 10.1146/annurev-med-051013-052304 PMC4324727 25341012

[120] T. Condamine , G. A. Dominguez , J. I. Youn , et al., “Lectin‐type Oxidized LDL Receptor‐1 Distinguishes Population of human Polymorphonuclear Myeloid‐derived Suppressor Cells in Cancer Patients,” Science Immunology 1, no. 2 (2016): aaf8943. 28417112 10.1126/sciimmunol.aaf8943 PMC5391495

[121] T. Nagasaki , M. Hara , H. Nakanishi , et al., “Interleukin‐6 Released by Colon Cancer‐associated Fibroblasts Is Critical for Tumour Angiogenesis: Anti‐interleukin‐6 Receptor Antibody Suppressed Angiogenesis and Inhibited Tumour‐stroma Interaction,” British Journal of Cancer 110, no. 2 (2014): 469–478. 24346288 10.1038/bjc.2013.748 PMC3899773

[122] J. Amersfoort , G. Eelen , and P. Carmeliet , “Immunomodulation by Endothelial Cells—partnering up With the Immune System?,” Nature Reviews Immunology 22, no. 9 (2022): 576–588. 10.1038/s41577-022-00694-4 PMC8920067 35288707

[123] Z. R. Huinen , E. J. M. Huijbers , J. R. van Beijnum , et al., “Anti‐angiogenic Agents—overcoming Tumour Endothelial Cell Anergy and Improving Immunotherapy Outcomes,” Nature Reviews Clinical Oncology 18, no. 8 (2021): 527–540. 10.1038/s41571-021-00496-y 33833434

[124] S. Azzi , J. K. Hebda , and J. Gavard , “Vascular Permeability and Drug Delivery in Cancers,” Frontiers in Oncology 3 (2013): 211. 23967403 10.3389/fonc.2013.00211 PMC3744053

[125] T. Stylianopoulos and R. K. Jain , “Combining Two Strategies to Improve Perfusion and Drug Delivery in Solid Tumors,” Proceedings of the National Academy of Sciences of the United States of America 110, no. 46 (2013): 18632–18637. 24167277 10.1073/pnas.1318415110 PMC3832007

[126] P. M. Glassman , J. W. Myerson , L. T. Ferguson , et al., “Targeting Drug Delivery in the Vascular System: Focus on Endothelium,” Advanced Drug Delivery Reviews 157 (2020): 96–117. 32579890 10.1016/j.addr.2020.06.013 PMC7306214

[127] C. Wang , J. Xu , Y. Zhang , et al., “Emerging Nanotechnological Approaches to Regulating Tumor Vasculature for Cancer Therapy,” Journal of Controlled Release: Official Journal of the Controlled Release Society 362 (2023): 647–666. 37703928 10.1016/j.jconrel.2023.09.017

[128] M. I. Setyawati , Q. Wang , N. Ni , et al., “Engineering Tumoral Vascular Leakiness With Gold Nanoparticles,” Nature Communications 14, no. 1 (2023): 4269. 10.1038/s41467-023-40015-4 PMC10352264 37460554

[129] P. W. Janes , A. C. Parslow , D. Cao , et al., “An Anti‐VEGF‐B Antibody Reduces Abnormal Tumor Vasculature and Enhances the Effects of Chemotherapy,” Cancers 16, no. 10 (2024): 1902. 38791979 10.3390/cancers16101902 PMC11119922

[130] E. Lopez‐Vince , C. Wilhelm , and T. Simon‐Yarza , “Vascularized Tumor Models for the Evaluation of Drug Delivery Systems: A Paradigm Shift,” Drug Delivery and Translational Research 14, no. 8 (2024): 2216–2241. 38619704 10.1007/s13346-024-01580-3 PMC11208221

[131] W. Wang , T. Li , Y. Cheng , et al., “Identification of Hypoxic Macrophages in Glioblastoma With Therapeutic Potential for Vasculature Normalization,” Cancer Cell 42, no. 5 (2024): 815–832. e812. 38640932 10.1016/j.ccell.2024.03.013

[132] L. C. Böckelmann and U. Schumacher , “Targeting Tumor Interstitial Fluid Pressure: Will It Yield Novel Successful Therapies for Solid Tumors?,” Expert Opinion on Therapeutic Targets 23, no. 12 (2019): 1005–1014. 31825703 10.1080/14728222.2019.1702974

[133] Q. Chang , O. I. Ornatsky , I. Siddiqui , et al., “Biodistribution of Cisplatin Revealed by Imaging Mass Cytometry Identifies Extensive Collagen Binding in Tumor and Normal Tissues,” Scientific Reports 6 (2016): 36641. 27812005 10.1038/srep36641 PMC5095658

[134] J. D. Martin , G. Seano , and R. K. Jain , “Normalizing Function of Tumor Vessels: Progress, Opportunities, and Challenges,” Annual Review of Physiology 81 (2019): 505–534. 10.1146/annurev-physiol-020518-114700 PMC6571025 30742782

[135] A. G. Sorensen , K. E. Emblem , P. Polaskova , et al., “Increased Survival of Glioblastoma Patients Who Respond to Antiangiogenic Therapy With Elevated Blood Perfusion,” Cancer Research 72, no. 2 (2012): 402–407. 22127927 10.1158/0008-5472.CAN-11-2464 PMC3261301

[136] H. Duan , C. Shao , Z. Luo , et al., “Perioperative sintilimab and Neoadjuvant anlotinib plus Chemotherapy for Resectable Non‐small‐cell Lung Cancer: A Multicentre, Open‐label, Single‐arm, Phase 2 Trial (TD‐NeoFOUR trial),” Signal Transduction and Targeted Therapy 9, no. 1 (2024): 296. 39465257 10.1038/s41392-024-01992-0 PMC11514280

[137] L. Colby , C. Preskitt , J. S. Ho , et al., “Brain Metastasis: A Literary Review of the Possible Relationship between Hypoxia and Angiogenesis in the Growth of Metastatic Brain Tumors,” International Journal of Molecular Sciences 26, no. 15 (2025): 7541. 40806669 10.3390/ijms26157541 PMC12347634

[138] J. Peng , W. Zhu , C. Zhang , et al., “COL4A2 drives ECM Remodeling and Stiffness Increasing to Promote Breast Cancer Metastasis via YAP Signaling Pathway in dECM Induced Models,” Biomaterials Advances 177 (2025): 214430. 40714253 10.1016/j.bioadv.2025.214430

[139] H. Peng , Z. Chao , Z. Wang , et al., “Biomechanics in the Tumor Microenvironment: From Biological Functions to Potential Clinical Applications,” Experimental Hematology & Oncology 14, no. 1 (2025): 4. 39799341 10.1186/s40164-024-00591-7 PMC11724500

[140] O. Maiques , M. C. Sallan , R. Laddach , et al., “Matrix Mechano‐sensing at the Invasive front Induces a Cytoskeletal and Transcriptional Memory Supporting Metastasis,” Nature Communications 16, no. 1 (2025): 1394. 10.1038/s41467-025-56299-7 PMC11829002 39952917

[141] A. Labernadie , T. Kato , A. Brugués , et al., “A Mechanically Active Heterotypic E‐cadherin/N‐cadherin Adhesion Enables Fibroblasts to Drive Cancer Cell Invasion,” Nature Cell Biology 19, no. 3 (2017): 224–237. 28218910 10.1038/ncb3478 PMC5831988

[142] W. J. Chen , C. C. Ho , Y. L. Chang , et al., “Cancer‐associated Fibroblasts Regulate the Plasticity of Lung Cancer Stemness via Paracrine Signalling,” Nature Communications 5 (2014): 3472. 10.1038/ncomms4472 24668028

[143] Y. Yu , C. H. Xiao , L. D. Tan , et al., “Cancer‐associated Fibroblasts Induce Epithelial‐mesenchymal Transition of Breast Cancer Cells Through Paracrine TGF‐β Signalling,” British Journal of Cancer 110, no. 3 (2014): 724–732. 24335925 10.1038/bjc.2013.768 PMC3915130

[144] K. M. Tharp , K. Kersten , O. Maller , et al., “Tumor‐associated Macrophages Restrict CD8(+) T Cell Function Through Collagen Deposition and Metabolic Reprogramming of the Breast Cancer Microenvironment,” Nature Cancer 5, no. 7 (2024): 1045–1062. 38831058 10.1038/s43018-024-00775-4 PMC12204312

[145] M. M. LaRue , S. Parker , J. Puccini , et al., “Metabolic Reprogramming of Tumor‐associated Macrophages by Collagen Turnover Promotes Fibrosis in Pancreatic Cancer,” Proceedings of the National Academy of Sciences of the United States of America 119, no. 16 (2022): e2119168119. 35412885 10.1073/pnas.2119168119 PMC9169723

[146] M. Demers , S. L. Wong , K. Martinod , et al., “Priming of Neutrophils Toward NETosis Promotes Tumor Growth,” Oncoimmunology 5, no. 5 (2016): e1134073. 27467952 10.1080/2162402X.2015.1134073 PMC4910712

[147] M. He , A. Peng , X. Z. Huang , et al., “Peritumoral Stromal Neutrophils Are Essential for c‐Met‐elicited Metastasis in human Hepatocellular Carcinoma,” Oncoimmunology 5, no. 10 (2016): e1219828. 27853643 10.1080/2162402X.2016.1219828 PMC5087290

[148] V. Callao and E. Montoya , “Toxohormone‐Like Factor From Microorganisms With Impaired Respiration,” Science (New York, NY) 134, no. 3495 (1961): 2041–2042. 10.1126/science.134.3495.2041 13875778

[149] L. K. Boroughs and R. J. DeBerardinis , “Metabolic Pathways Promoting Cancer Cell Survival and Growth,” Nature Cell Biology 17, no. 4 (2015): 351–359. 25774832 10.1038/ncb3124 PMC4939711

[150] H. J. Hurley , H. Dewald , Z. S. Rothkopf , et al., “Frontline Science: AMPK Regulates Metabolic Reprogramming Necessary for Interferon Production in human Plasmacytoid Dendritic Cells,” Journal of Leukocyte Biology 109, no. 2 (2021): 299–308. 32640499 10.1002/JLB.3HI0220-130

[151] L. Sun , L. Song , Q. Wan , et al., “cMyc‐mediated Activation of Serine Biosynthesis Pathway Is Critical for Cancer Progression Under Nutrient Deprivation Conditions,” Cell Research 25, no. 4 (2015): 429–444. 25793315 10.1038/cr.2015.33 PMC4387561

[152] Z. T. Schug , J. Vande Voorde , and E. Gottlieb , “The Metabolic Fate of Acetate in Cancer,” Nature Reviews Cancer 16, no. 11 (2016): 708–717. 27562461 10.1038/nrc.2016.87 PMC8992383

[153] Z. T. Schug , B. Peck , D. T. Jones , et al., “Acetyl‐CoA Synthetase 2 Promotes Acetate Utilization and Maintains Cancer Cell Growth Under Metabolic Stress,” Cancer Cell 27, no. 1 (2015): 57–71. 25584894 10.1016/j.ccell.2014.12.002 PMC4297291

[154] T. Mashimo , K. Pichumani , V. Vemireddy , et al., “Acetate Is a Bioenergetic Substrate for human Glioblastoma and Brain Metastases,” Cell 159, no. 7 (2014): 1603–1614. 25525878 10.1016/j.cell.2014.11.025 PMC4374602

[155] E. L. Pearce and E. J. Pearce , “Metabolic Pathways in Immune Cell Activation and Quiescence,” Immunity 38, no. 4 (2013): 633–643. 23601682 10.1016/j.immuni.2013.04.005 PMC3654249

[156] C. H. Chang , J. Qiu , D. O'Sullivan , et al., “Metabolic Competition in the Tumor Microenvironment Is a Driver of Cancer Progression,” Cell 162, no. 6 (2015): 1229–1241. 26321679 10.1016/j.cell.2015.08.016 PMC4864363

[157] K. Singer , M. Kastenberger , E. Gottfried , et al., “Warburg Phenotype in Renal Cell Carcinoma: High Expression of Glucose‐transporter 1 (GLUT‐1) Correlates With Low CD8(+) T‐cell Infiltration in the Tumor,” International Journal of Cancer 128, no. 9 (2011): 2085–2095. 20607826 10.1002/ijc.25543

[158] J. Zhang , J. Yang , C. Lin , et al., “Endoplasmic Reticulum Stress‐dependent Expression of ERO1L Promotes Aerobic Glycolysis in Pancreatic Cancer,” Theranostics 10, no. 18 (2020): 8400–8414. 32724477 10.7150/thno.45124 PMC7381747

[159] A. Domiński , M. Krawczyk , T. Konieczny , et al., “Biodegradable pH‐responsive Micelles Loaded With 8‐hydroxyquinoline Glycoconjugates for Warburg Effect Based Tumor Targeting,” European Journal of Pharmaceutics and Biopharmaceutics: Official Journal of Arbeitsgemeinschaft Fur Pharmazeutische Verfahrenstechnik eV 154 (2020): 317–329. 10.1016/j.ejpb.2020.07.019 32717390

[160] B. J. Altman , Z. E. Stine , and C. V. Dang , “From Krebs to Clinic: Glutamine Metabolism to Cancer Therapy,” Nature Reviews Cancer 16, no. 11 (2016): 749. 10.1038/nrc.2016.114 28704361

[161] B. I. Reinfeld , M. Z. Madden , M. M. Wolf , et al., “Cell‐programmed Nutrient Partitioning in the Tumour Microenvironment,” Nature 593, no. 7858 (2021): 282–288. 33828302 10.1038/s41586-021-03442-1 PMC8122068

[162] B. Peck and A. Schulze , “Lipid Metabolism at the Nexus of Diet and Tumor Microenvironment,” Trends in Cancer 5, no. 11 (2019): 693–703. 31735288 10.1016/j.trecan.2019.09.007

[163] A. M. Kleinfeld and C. Okada , “Free Fatty Acid Release From human Breast Cancer Tissue Inhibits Cytotoxic T‐lymphocyte‐mediated Killing,” Journal of Lipid Research 46, no. 9 (2005): 1983–1990. 15961785 10.1194/jlr.M500151-JLR200

[164] X. Ma , E. Bi , Y. Lu , et al., “Cholesterol Induces CD8(+) T Cell Exhaustion in the Tumor Microenvironment,” Cell Metabolism 30, no. 1 (2019): 143–156. e145. 31031094 10.1016/j.cmet.2019.04.002 PMC7061417

[165] F. Lopes‐Coelho , S. André , A. Félix , et al., “Breast Cancer Metabolic Cross‐talk: Fibroblasts Are Hubs and Breast Cancer Cells Are Gatherers of Lipids,” Molecular and Cellular Endocrinology 462 (2018): 93–106. Pt B. 28119133 10.1016/j.mce.2017.01.031

[166] A. Santi , A. Caselli , F. Ranaldi , et al., “Cancer Associated Fibroblasts Transfer Lipids and Proteins to Cancer Cells Through Cargo Vesicles Supporting Tumor Growth,” Biochimica Et Biophysica Acta 1853, no. 12 (2015): 3211–3223. 26384873 10.1016/j.bbamcr.2015.09.013

[167] Y. Zhang , R. Kurupati , L. Liu , et al., “Enhancing CD8(+) T Cell Fatty Acid Catabolism Within a Metabolically Challenging Tumor Microenvironment Increases the Efficacy of Melanoma Immunotherapy,” Cancer Cell 32, no. 3 (2017): 377–391. e379. 28898698 10.1016/j.ccell.2017.08.004 PMC5751418

[168] S. Romero‐Garcia , M. M. Moreno‐Altamirano , H. Prado‐Garcia , et al., “Lactate Contribution to the Tumor Microenvironment: Mechanisms, Effects on Immune Cells and Therapeutic Relevance,” Frontiers in Immunology 7 (2016): 52. 26909082 10.3389/fimmu.2016.00052 PMC4754406

[169] O. R. Colegio , N. Q. Chu , A. L. Szabo , et al., “Functional Polarization of Tumour‐associated Macrophages by Tumour‐derived Lactic Acid,” Nature 513, no. 7519 (2014): 559–563. 25043024 10.1038/nature13490 PMC4301845

[170] L. Paolini , C. Adam , C. Beauvillain , et al., “Lactic Acidosis Together With GM‐CSF and M‐CSF Induces Human Macrophages Toward an Inflammatory Protumor Phenotype,” Cancer Immunology Research 8, no. 3 (2020): 383–395. 31924656 10.1158/2326-6066.CIR-18-0749

[171] H. Jeong , S. Kim , B. J. Hong , et al., “Tumor‐Associated Macrophages Enhance Tumor Hypoxia and Aerobic Glycolysis,” Cancer Research 79, no. 4 (2019): 795–806. 30610087 10.1158/0008-5472.CAN-18-2545

[172] A. Angelin , L. Gil‐de‐Gómez , S. Dahiya , et al., “Foxp3 Reprograms T Cell Metabolism to Function in Low‐Glucose, High‐Lactate Environments,” Cell Metabolism 25, no. 6 (2017): 1282–1293. e1287. 28416194 10.1016/j.cmet.2016.12.018 PMC5462872

[173] H. Han , S. Wang , L. Ma , et al., “ASH2L‐K312‐Lac Stimulates Angiogenesis in Tumors to Expedite the Malignant Progression of Hepatocellular Carcinoma,” Advanced Science (Weinheim, Baden‐Wurttemberg, Germany) 12, no. 40 (2025): e09477. 40726441 10.1002/advs.202509477 PMC12561209

[174] F. Ghadyani , P. Zandi , and S. Ghafouri‐Fard , “Histone Lactylation: A New Target for Overcoming Immune Evasion and Therapy Resistance,” Medical Oncology (Northwood, London, England) 42, no. 9 (2025): 399. 40753273 10.1007/s12032-025-02940-w

[175] H. Ren , Y. Tang , and D. Zhang , “The Emerging Role of Protein L‐lactylation in Metabolic Regulation and Cell Signalling,” Nature Metabolism 7, no. 4 (2025): 647–664. 10.1038/s42255-025-01259-0 40175761

[176] S. Missiroli , M. Perrone , I. Genovese , et al., “Cancer Metabolism and Mitochondria: Finding Novel Mechanisms to Fight Tumours,” EBioMedicine 59 (2020): 102943. 32818805 10.1016/j.ebiom.2020.102943 PMC7452656

[177] F. Weinberg , N. Ramnath , and D. Nagrath , “Reactive Oxygen Species in the Tumor Microenvironment: An Overview,” Cancers 11, no. 8 (2019): 1191. 31426364 10.3390/cancers11081191 PMC6721577

[178] N. S. Chandel , E. Maltepe , E. Goldwasser , et al., “Mitochondrial Reactive Oxygen Species Trigger Hypoxia‐induced Transcription,” Proceedings of the National Academy of Sciences of the United States of America 95, no. 20 (1998): 11715–11720. 9751731 10.1073/pnas.95.20.11715 PMC21706

[179] N. S. Chandel , D. S. McClintock , C. E. Feliciano , et al., “Reactive Oxygen Species Generated at Mitochondrial Complex III Stabilize Hypoxia‐inducible Factor‐1alpha During Hypoxia: A Mechanism of O2 Sensing,” The Journal of Biological Chemistry 275, no. 33 (2000): 25130–25138. 10833514 10.1074/jbc.M001914200

[180] L. A. Sena , S. Li , A. Jairaman , et al., “Mitochondria Are Required for Antigen‐specific T Cell Activation Through Reactive Oxygen Species Signaling,” Immunity 38, no. 2 (2013): 225–236. 23415911 10.1016/j.immuni.2012.10.020 PMC3582741

[181] A. Costa , A. Scholer‐Dahirel , and F. Mechta‐Grigoriou , “The Role of Reactive Oxygen Species and Metabolism on Cancer Cells and Their Microenvironment,” Seminars in Cancer Biology 25 (2014): 23–32. 24406211 10.1016/j.semcancer.2013.12.007

[182] M. S. Nakazawa , B. Keith , and M. C. Simon , “Oxygen Availability and Metabolic Adaptations,” Nature Reviews Cancer 16, no. 10 (2016): 663–673. 27658636 10.1038/nrc.2016.84 PMC5546320

[183] J. C. García‐Cañaveras , L. Chen , and J. D. Rabinowitz , “The Tumor Metabolic Microenvironment: Lessons From Lactate,” Cancer Research 79, no. 13 (2019): 3155–3162. 31171526 10.1158/0008-5472.CAN-18-3726 PMC6606343

[184] E. Boedtkjer and S. F. Pedersen , “The Acidic Tumor Microenvironment as a Driver of Cancer,” Annual Review of Physiology 82 (2020): 103–126. 10.1146/annurev-physiol-021119-034627 31730395

[185] C. Corbet and O. Feron , “Tumour Acidosis: From the Passenger to the Driver's Seat,” Nature Reviews Cancer 17, no. 10 (2017): 577–593. 28912578 10.1038/nrc.2017.77

[186] C. Magnon , S. J. Hall , J. Lin , et al., “Autonomic Nerve Development Contributes to Prostate Cancer Progression,” Science (New York, NY) 341, no. 6142 (2013): 1236361. 10.1126/science.1236361 23846904

[187] J. L. Saloman , K. M. Albers , A. D. Rhim , et al., “Can Stopping Nerves, Stop Cancer?,” Trends in Neurosciences 39, no. 12 (2016): 880–889. 27832915 10.1016/j.tins.2016.10.002 PMC5148708

[188] S. W. Cole , A. S. Nagaraja , S. K. Lutgendorf , et al., “Sympathetic Nervous System Regulation of the Tumour Microenvironment,” Nature Reviews Cancer 15, no. 9 (2015): 563–572. 26299593 10.1038/nrc3978 PMC4828959

[189] S. Faulkner , P. Jobling , B. March , et al., “Tumor Neurobiology and the War of Nerves in Cancer,” Cancer Discovery 9, no. 6 (2019): 702–710. 30944117 10.1158/2159-8290.CD-18-1398

[190] P. Jobling , J. Pundavela , S. M. Oliveira , et al., “Nerve‐Cancer Cell Cross‐talk: A Novel Promoter of Tumor Progression,” Cancer Research 75, no. 9 (2015): 1777–1781. 25795709 10.1158/0008-5472.CAN-14-3180

[191] N. M. E. Ayad and V. M. Weaver , “Tension in Tumour Cells Keeps Metabolism High,” Nature 578, no. 7796 (2020): 517–518. 32094916 10.1038/d41586-020-00314-y

[192] Y. Liu , J. Lv , X. Liang , et al., “Fibrin Stiffness Mediates Dormancy of Tumor‐Repopulating Cells via a Cdc42‐Driven Tet2 Epigenetic Program,” Cancer Research 78, no. 14 (2018): 3926–3937. 29764867 10.1158/0008-5472.CAN-17-3719

[193] J. S. Park , C. J. Burckhardt , R. Lazcano , et al., “Mechanical Regulation of Glycolysis via Cytoskeleton Architecture,” Nature 578, no. 7796 (2020): 621–626. 32051585 10.1038/s41586-020-1998-1 PMC7210009

[194] T. A. Ulrich , J. E. M. de Pardo , and S. Kumar , “The Mechanical Rigidity of the Extracellular Matrix Regulates the Structure, Motility, and Proliferation of Glioma Cells,” Cancer Research 69, no. 10 (2009): 4167–4174. 19435897 10.1158/0008-5472.CAN-08-4859 PMC2727355

[195] K. M. Wisdom , K. Adebowale , J. Chang , et al., “Matrix Mechanical Plasticity Regulates Cancer Cell Migration Through Confining Microenvironments,” Nature Communications 9, no. 1 (2018): 4144. 10.1038/s41467-018-06641-z PMC6175826 30297715

[196] P. Sonveaux , F. Végran , T. Schroeder , et al., “Targeting Lactate‐fueled Respiration Selectively Kills Hypoxic Tumor Cells in Mice,” The Journal of Clinical Investigation 118, no. 12 (2008): 3930–3942. 19033663 10.1172/JCI36843 PMC2582933

[197] J. Chiche , M. C. Brahimi‐Horn , and J. Pouysségur , “Tumour Hypoxia Induces a Metabolic Shift Causing Acidosis: A Common Feature in Cancer,” Journal of Cellular and Molecular Medicine 14, no. 4 (2010): 771–794. 20015196 10.1111/j.1582-4934.2009.00994.x PMC3823111

[198] A. H. Zahalka and P. S. Frenette , “Nerves in Cancer,” Nature Reviews Cancer 20, no. 3 (2020): 143–157. 31974491 10.1038/s41568-019-0237-2 PMC7709871

[199] C. Liebig , G. Ayala , J. A. Wilks , et al., “Perineural Invasion in Cancer: A Review of the Literature,” Cancer 115, no. 15 (2009): 3379–3391. 19484787 10.1002/cncr.24396

[200] H. You , W. Shang , X. Min , et al., “Sight and Switch off: Nerve Density Visualization for Interventions Targeting Nerves in Prostate Cancer,” Science Advances 6, no. 6 (2020): eaax6040. 32076639 10.1126/sciadv.aax6040 PMC7002130

[201] C. Cao , “Targeting Tumor‐associated Nerves Enhances Cancer Immunotherapy,” Neuron 113, no. 19 (2025): 3076–3078. 41038157 10.1016/j.neuron.2025.09.005

[202] N. Khanmammadova , S. Islam , P. Sharma , et al., “Neuro‐immune Interactions and Immuno‐oncology,” Trends in Cancer 9, no. 8 (2023): 636–649. 37258398 10.1016/j.trecan.2023.05.002 PMC10524972

[203] M. Amit , T. Eichwald , A. Roger , et al., “Neuro‐immune Cross‐talk in Cancer,” Nature Reviews Cancer 25, no. 8 (2025): 573–589. 40523971 10.1038/s41568-025-00831-w PMC13142818

[204] L. B. Darragh , A. Nguyen , T. T. Pham , et al., “Sensory Nerve Release of CGRP Increases Tumor Growth in HNSCC by Suppressing TILs,” Med (New York, NY) 5, no. 3 (2024): 254–270. e258. 10.1016/j.medj.2024.02.002 PMC10939743 38423011

[205] A. A. Martel Matos and N. N. Scheff , “Sensory Neurotransmission and Pain in Solid Tumor Progression,” Trends in Cancer 11, no. 4 (2025): 309–320. 39884880 10.1016/j.trecan.2025.01.003 PMC12100539

[206] Y. Liu , H. Wang , X. Zhao , et al., “Targeting the Immunoglobulin IGSF9 Enhances Antitumor T‐cell Activity and Sensitivity to Anti‐PD‐1 Immunotherapy,” Cancer Research 83, no. 20 (2023): 3385–3399. 37506192 10.1158/0008-5472.CAN-22-3115

[207] K. Y. Lee , Y. Mei , H. Liu , et al., “CD137‐expressing Regulatory T Cells in Cancer and Autoimmune Diseases,” Molecular Therapy: the Journal of the American Society of Gene Therapy 33, no. 1 (2025): 51–70. 39668561 10.1016/j.ymthe.2024.12.010 PMC11764688

[208] M. L. Dixon , L. Luo , S. Ghosh , et al., “Remodeling of the Tumor Microenvironment via Disrupting Blimp1(+) Effector Treg Activity Augments Response to Anti‐PD‐1 Blockade,” Molecular Cancer 20, no. 1 (2021): 150. 34798898 10.1186/s12943-021-01450-3 PMC8605582

[209] T. Pu , J. Sun , G. Ren , et al., “Neuro‐immune Crosstalk in Cancer: Mechanisms and Therapeutic Implications,” Signal Transduction and Targeted Therapy 10, no. 1 (2025): 176. 40456735 10.1038/s41392-025-02241-8 PMC12130251

[210] C. Shen , J. Liu , D. Hu , et al., “Tumor‐intrinsic ENO1 Inhibition Promotes Antitumor Immune Response and Facilitates the Efficacy of Anti‐PD‐L1 Immunotherapy in Bladder Cancer,” Journal of Experimental & Clinical Cancer Research: CR 44, no. 1 (2025): 207. 40665335 10.1186/s13046-025-03464-x PMC12261641

[211] Y. Zhu , K. F. Brulois , T. T. Dinh , et al., “COUP‐TFII‐mediated Reprogramming of the Vascular Endothelium Counteracts Tumor Immune Evasion,” Nature Communications 16, no. 1 (2025): 7457. 10.1038/s41467-025-62399-1 PMC12343902 40796746

[212] A. Nagelkerke , J. Bussink , A. E. Rowan , et al., “The Mechanical Microenvironment in Cancer: How Physics Affects Tumours,” Seminars in Cancer Biology 35 (2015): 62–70. 26343578 10.1016/j.semcancer.2015.09.001

[213] R. Kalluri and M. Zeisberg , “Fibroblasts in Cancer,” Nature Reviews Cancer 6, no. 5 (2006): 392–401. 16572188 10.1038/nrc1877

[214] G. S. Offeddu , E. Cambria , S. E. Shelton , et al., “Personalized Vascularized Models of Breast Cancer Desmoplasia Reveal Biomechanical Determinants of Drug Delivery to the Tumor,” Advanced Science (Weinheim, Baden‐Wurttemberg, Germany) 11, no. 38 (2024): e2402757. 39041892 10.1002/advs.202402757 PMC11481247

[215] T. Panciera , L. Azzolin , M. Cordenonsi , et al., “Mechanobiology of YAP and TAZ in Physiology and Disease,” Nature Reviews Molecular Cell Biology 18, no. 12 (2017): 758–770. 28951564 10.1038/nrm.2017.87 PMC6192510

[216] L. Vermeulen , E. M. F. De Sousa , M. van der Heijden , et al., “Wnt Activity Defines Colon Cancer Stem Cells and Is Regulated by the Microenvironment,” Nature Cell Biology 12, no. 5 (2010): 468–476. 20418870 10.1038/ncb2048

[217] F. Calvo , N. Ege , A. Grande‐Garcia , et al., “Mechanotransduction and YAP‐dependent Matrix Remodelling Is Required for the Generation and Maintenance of Cancer‐associated Fibroblasts,” Nature Cell Biology 15, no. 6 (2013): 637–646. 23708000 10.1038/ncb2756 PMC3836234

[218] M. R. Junttila and F. J. de Sauvage , “Influence of Tumour Micro‐environment Heterogeneity on Therapeutic Response,” Nature 501, no. 7467 (2013): 346–354. 24048067 10.1038/nature12626

[219] M. Li , X. Zhang , M. Wang , et al., “Activation of Piezo1 Contributes to Matrix Stiffness‐induced Angiogenesis in Hepatocellular Carcinoma,” Cancer Communications (London, England) 42, no. 11 (2022): 1162–1184. 36181398 10.1002/cac2.12364 PMC9648387

[220] P. V. Taufalele , W. Wang , A. J. Simmons , et al., “Matrix Stiffness Enhances Cancer‐macrophage Interactions and M2‐Like Macrophage Accumulation in the Breast Tumor Microenvironment,” Acta Biomaterialia 163 (2023): 365–377. 35483629 10.1016/j.actbio.2022.04.031 PMC9592676

[221] K. Esbona , D. Inman , S. Saha , et al., “COX‐2 Modulates Mammary Tumor Progression in Response to Collagen Density,” Breast Cancer Research: BCR 18, no. 1 (2016): 35. 27000374 10.1186/s13058-016-0695-3 PMC4802888

[222] Z. Zhao , F. Han , S. Yang , et al., “Oxamate‐mediated Inhibition of Lactate Dehydrogenase Induces Protective Autophagy in Gastric Cancer Cells: Involvement of the Akt‐mTOR Signaling Pathway,” Cancer Letters 358, no. 1 (2015): 17–26. 25524555 10.1016/j.canlet.2014.11.046

[223] J. Papaconstantinou and S. P. Colowick , “The Role of Glycolysis in the Growth of Tumor Cells. II. The Effect of Oxamic Acid on the Growth of HeLa Cells in Tissue Culture,” The Journal of Biological Chemistry 236 (1961): 285–288. 13732587

[224] A. Boudreau , H. E. Purkey , A. Hitz , et al., “Metabolic Plasticity Underpins Innate and Acquired Resistance to LDHA Inhibition,” Nature Chemical Biology 12, no. 10 (2016): 779–786. 27479743 10.1038/nchembio.2143

[225] V. L. Payen , E. Mina , and V. F. Van Hée , “Monocarboxylate Transporters in Cancer,” Molecular Metabolism 33 (2020): 48–66. 31395464 10.1016/j.molmet.2019.07.006 PMC7056923

[226] M. Kobayashi , K. Narumi , A. Furugen , et al., “Transport Function, Regulation, and Biology of human Monocarboxylate Transporter 1 (hMCT1) and 4 (hMCT4),” Pharmacology & Therapeutics 226 (2021): 107862. 33894276 10.1016/j.pharmthera.2021.107862

[227] N. Draoui , O. Schicke , E. Seront , et al., “Antitumor Activity of 7‐aminocarboxycoumarin Derivatives, a New Class of Potent Inhibitors of Lactate Influx but Not Efflux,” Molecular Cancer Therapeutics 13, no. 6 (2014): 1410–1418. 24672058 10.1158/1535-7163.MCT-13-0653

[228] C. Corbet , E. Bastien , N. Draoui , et al., “Interruption of Lactate Uptake by Inhibiting Mitochondrial Pyruvate Transport Unravels Direct Antitumor and Radiosensitizing Effects,” Nature Communications 9, no. 1 (2018): 1208. 10.1038/s41467-018-03525-0 PMC5865202 29572438

[229] X. Guan , V. Rodriguez‐Cruz , and M. E. Morris , “Cellular Uptake of MCT1 Inhibitors AR‐C155858 and AZD3965 and Their Effects on MCT‐Mediated Transport of L‐Lactate in Murine 4T1 Breast Tumor Cancer Cells,” The AAPS Journal 21, no. 2 (2019): 13. 30617815 10.1208/s12248-018-0279-5 PMC6466617

[230] M. Quanz , E. Bender , C. Kopitz , et al., “Preclinical Efficacy of the Novel Monocarboxylate Transporter 1 Inhibitor BAY‐8002 and Associated Markers of Resistance,” Molecular Cancer Therapeutics 17, no. 11 (2018): 2285–2296. 30115664 10.1158/1535-7163.MCT-17-1253

[231] M. J. Watson , P. D. A. Vignali , S. J. Mullett , et al., “Metabolic Support of Tumour‐infiltrating Regulatory T Cells by Lactic Acid,” Nature 591, no. 7851 (2021): 645–651. 33589820 10.1038/s41586-020-03045-2 PMC7990682

[232] M. M. G. Kes , J. Van den Bossche , A. W. Griffioen , et al., “Oncometabolites Lactate and Succinate Drive Pro‐angiogenic Macrophage Response in Tumors,” Biochimica Et Biophysica Acta Reviews on Cancer 1874, no. 2 (2020): 188427. 32961257 10.1016/j.bbcan.2020.188427

[233] K. Renner , C. Bruss , A. Schnell , et al., “Restricting Glycolysis Preserves T Cell Effector Functions and Augments Checkpoint Therapy,” Cell Reports 29, no. 1 (2019): 135–150. e139. 31577944 10.1016/j.celrep.2019.08.068

[234] H. Li , X. Li , S. Liu , et al., “Programmed Cell Death‐1 (PD‐1) Checkpoint Blockade in Combination With a Mammalian Target of rapamycin Inhibitor Restrains Hepatocellular Carcinoma Growth Induced by Hepatoma Cell‐intrinsic PD‐1,” Hepatology (Baltimore, Md) 66, no. 6 (2017): 1920–1933. 10.1002/hep.29360 28732118

[235] E. Vitali , I. Boemi , G. Tarantola , et al., “Metformin and Everolimus: A Promising Combination for Neuroendocrine Tumors Treatment,” Cancers 12, no. 8 (2020): 2143. 32748870 10.3390/cancers12082143 PMC7464161

[236] S. E. Weinberg and N. S. Chandel , “Mitochondria Reactive Oxygen Species Signaling‐dependent Immune Responses in Macrophages and T Cells,” Immunity 58, no. 8 (2025): 1904–1921. 40763730 10.1016/j.immuni.2025.07.012 PMC12371701

[237] Y. Lv , Z. Li , S. Liu , et al., “Metabolic Checkpoints in Immune Cell Reprogramming: Rewiring Immunometabolism for Cancer Therapy,” Molecular Cancer 24, no. 1 (2025): 210. 40753443 10.1186/s12943-025-02407-6 PMC12318400

[238] J. Alexandre , Y. Hu , W. Lu , et al., “Novel Action of paclitaxel Against Cancer Cells: Bystander Effect Mediated by Reactive Oxygen Species,” Cancer Research 67, no. 8 (2007): 3512–3517. 17440056 10.1158/0008-5472.CAN-06-3914

[239] T. Pecchillo Cimmino , R. Ammendola , F. Cattaneo , et al., “NOX Dependent ROS Generation and Cell Metabolism,” International Journal of Molecular Sciences 24, no. 3 (2023): 2086. 36768405 10.3390/ijms24032086 PMC9916913

[240] T. Ishimoto , O. Nagano , T. Yae , et al., “CD44 variant Regulates Redox Status in Cancer Cells by Stabilizing the xCT Subunit of System Xc(‐) and Thereby Promotes Tumor Growth,” Cancer Cell 19, no. 3 (2011): 387–400. 21397861 10.1016/j.ccr.2011.01.038

[241] B. Chen , Y. Song , Y. Zhan , et al., “Fangchinoline Inhibits Non‐small Cell Lung Cancer Metastasis by Reversing Epithelial‐mesenchymal Transition and Suppressing the Cytosolic ROS‐related Akt‐mTOR Signaling Pathway,” Cancer Letters 543 (2022): 215783. 35700820 10.1016/j.canlet.2022.215783

[242] K. Ford , C. J. Hanley , M. Mellone , et al., “NOX4 Inhibition Potentiates Immunotherapy by Overcoming Cancer‐Associated Fibroblast‐Mediated CD8 T‐cell Exclusion From Tumors,” Cancer Research 80, no. 9 (2020): 1846–1860. 32122909 10.1158/0008-5472.CAN-19-3158 PMC7611230

[243] D. Trachootham , G. Chen , W. Zhang , et al., “Loss of p53 in Stromal Fibroblasts Promotes Epithelial Cell Invasion Through Redox‐mediated ICAM1 Signal,” Free Radical Biology & Medicine 58 (2013): 1–13. 23376231 10.1016/j.freeradbiomed.2013.01.011 PMC3622735

[244] Z. Huang , Q. Su , W. Li , et al., “Suppressed Mitochondrial Respiration via NOX5‐mediated Redox Imbalance Contributes to the Antitumor Activity of anlotinib in Oral Squamous Cell Carcinoma,” Journal of Genetics and Genomics = Yi Chuan Xue Bao 48, no. 7 (2021): 582–594. 34373220 10.1016/j.jgg.2021.06.014

[245] X. Liu , H. Cui , M. Li , et al., “Tumor Killing by a Dietary Curcumin Mono‐carbonyl Analog That Works as a Selective ROS Generator via TrxR Inhibition,” European Journal of Medicinal Chemistry 250 (2023): 115191. 36758308 10.1016/j.ejmech.2023.115191

[246] S. Peng , S. Yu , J. Zhang , et al., “6‐Shogaol as a Novel Thioredoxin Reductase Inhibitor Induces Oxidative‐Stress‐Mediated Apoptosis in HeLa Cells,” International Journal of Molecular Sciences 24, no. 5 (2023): 4966. 36902397 10.3390/ijms24054966 PMC10003455

[247] X. Liu , Y. Zhang , W. Lu , et al., “Mitochondrial TXNRD3 Confers Drug Resistance via Redox‐mediated Mechanism and Is a Potential Therapeutic Target in Vivo,” Redox Biology 36 (2020): 101652. 32750669 10.1016/j.redox.2020.101652 PMC7397405

[248] X. Ma , L. Xiao , L. Liu , et al., “CD36‐mediated Ferroptosis Dampens Intratumoral CD8(+) T Cell Effector Function and Impairs Their Antitumor Ability,” Cell Metabolism 33, no. 5 (2021): 1001–1012. e1005. 33691090 10.1016/j.cmet.2021.02.015 PMC8102368

[249] F. Veglia , V. A. Tyurin , M. Blasi , et al., “Fatty Acid Transport Protein 2 Reprograms Neutrophils in Cancer,” Nature 569, no. 7754 (2019): 73–78. 30996346 10.1038/s41586-019-1118-2 PMC6557120

[250] M. Zhang , J. S. Di Martino , R. L. Bowman , et al., “Adipocyte‐Derived Lipids Mediate Melanoma Progression via FATP Proteins,” Cancer Discovery 8, no. 8 (2018): 1006–1025. 29903879 10.1158/2159-8290.CD-17-1371 PMC6192670

[251] K. J. Thompson , R. G. Austin , S. S. Nazari , et al., “Altered Fatty Acid‐binding Protein 4 (FABP4) Expression and Function in human and Animal Models of Hepatocellular Carcinoma,” Liver International: Official Journal of the International Association for the Study of the Liver 38, no. 6 (2018): 1074–1083. 29171144 10.1111/liv.13639

[252] N. Osinalde , J. Mitxelena , V. Sánchez‐Quiles , et al., “Nuclear Phosphoproteomic Screen Uncovers ACLY as Mediator of IL‐2‐induced Proliferation of CD4+ T Lymphocytes,” Molecular & Cellular Proteomics: MCP 15, no. 6 (2016): 2076–2092. 27067055 10.1074/mcp.M115.057158 PMC5083085

[253] D. Trachootham , H. Zhang , W. Zhang , et al., “Effective Elimination of Fludarabine‐resistant CLL Cells by PEITC Through a Redox‐mediated Mechanism,” Blood 112, no. 5 (2008): 1912–1922. 18574029 10.1182/blood-2008-04-149815 PMC2518893

[254] A. Lam‐Ubol , A. L. Fitzgerald , A. Ritdej , et al., “Sensory Acceptable Equivalent Doses of β‐phenylethyl Isothiocyanate (PEITC) Induce Cell Cycle Arrest and Retard the Growth of p53 Mutated Oral Cancer in Vitro and in Vivo,” Food & Function 9, no. 7 (2018): 3640–3656. 29923573 10.1039/c8fo00865e

[255] Y. Sun , N. Berleth , W. Wu , et al., “Fin56‐induced Ferroptosis Is Supported by Autophagy‐mediated GPX4 Degradation and Functions Synergistically With mTOR Inhibition to Kill Bladder Cancer Cells,” Cell Death & Disease 12, no. 11 (2021): 1028. 34716292 10.1038/s41419-021-04306-2 PMC8556316

[256] G. Augello , A. Azzolina , F. Rossi , et al., “New Insights Into the Behavior of NHC‐Gold Complexes in Cancer Cells,” Pharmaceutics 15, no. 2 (2023): 466. 36839788 10.3390/pharmaceutics15020466 PMC9963827

[257] G. Pascual , A. Avgustinova , S. Mejetta , et al., “Targeting Metastasis‐initiating Cells Through the Fatty Acid Receptor CD36,” Nature 541, no. 7635 (2017): 41–45. 27974793 10.1038/nature20791

[258] M. Dominguez , B. Brüne , and D. Namgaladze , “Exploring the Role of ATP‐Citrate Lyase in the Immune System,” Frontiers in Immunology 12 (2021): 632526. 33679780 10.3389/fimmu.2021.632526 PMC7930476

[259] C. Granchi , “ATP Citrate Lyase (ACLY) Inhibitors: An Anti‐cancer Strategy at the Crossroads of Glucose and Lipid Metabolism,” European Journal of Medicinal Chemistry 157 (2018): 1276–1291. 30195238 10.1016/j.ejmech.2018.09.001

[260] C. J. Li , Y. H. Chiu , C. Chang , et al., “Acetyl Coenzyme A Synthase 2 Acts as a Prognostic Biomarker Associated With Immune Infiltration in Cervical Squamous Cell Carcinoma,” Cancers 13, no. 13 (2021): 3125. 34206705 10.3390/cancers13133125 PMC8269092

[261] D. Cao , J. Yang , Y. Deng , et al., “Discovery of a Mammalian FASN Inhibitor Against Xenografts of Non‐small Cell Lung Cancer and Melanoma,” Signal Transduction and Targeted Therapy 7, no. 1 (2022): 273. 36002450 10.1038/s41392-022-01099-4 PMC9402528

[262] S. Zheng , Q. Song , and P. Zhang , “Metabolic Modifications, Inflammation, and Cancer Immunotherapy,” Frontiers in Oncology 11 (2021): 703681. 34631531 10.3389/fonc.2021.703681 PMC8497755

[263] J. Gautam , J. Wu , J. S. V. Lally , et al., “ACLY Inhibition Promotes Tumour Immunity and Suppresses Liver Cancer,” Nature 645, no. 8080 (2025): 507–517. 40739358 10.1038/s41586-025-09297-0 PMC12422966

[264] S. Huang , Y. Liu , M. Zhao , et al., “Copy Number Amplification of TTPAL Promotes Cholesterol Biosynthesis and Esophageal Squamous Cell Carcinoma Progression via Elevating NSUN2‐mediated m5C Modification of SREBP2 mRNA,” Journal of Experimental & Clinical Cancer Research: CR 44, no. 1 (2025): 220. 40713894 10.1186/s13046-025-03483-8 PMC12297799

[265] G. L. Semenza , “Development of Small Molecule Inhibitors of Hypoxia‐inducible Factors for Cancer Therapy,” Pharmacological Reviews 77, no. 5 (2025): 100075. 40743978 10.1016/j.pharmr.2025.100075

[266] J. M. Ebos and R. S. Kerbel , “Antiangiogenic Therapy: Impact on Invasion, Disease Progression, and Metastasis,” Nature Reviews Clinical Oncology 8, no. 4 (2011): 210–221. 10.1038/nrclinonc.2011.21 PMC4540336 21364524

[267] Y. Huang , S. Goel , D. G. Duda , et al., “Vascular Normalization as an Emerging Strategy to Enhance Cancer Immunotherapy,” Cancer Research 73, no. 10 (2013): 2943–2948. 23440426 10.1158/0008-5472.CAN-12-4354 PMC3655127

[268] Y. Huang , J. Yuan , E. Righi , et al., “Vascular Normalizing Doses of Antiangiogenic Treatment Reprogram the Immunosuppressive Tumor Microenvironment and Enhance Immunotherapy,” Proceedings of the National Academy of Sciences of the United States of America 109, no. 43 (2012): 17561–17566. 23045683 10.1073/pnas.1215397109 PMC3491458

[269] F. S. Hodi , D. Lawrence , C. Lezcano , et al., “Bevacizumab plus ipilimumab in Patients With Metastatic Melanoma,” Cancer Immunology Research 2, no. 7 (2014): 632–642. 24838938 10.1158/2326-6066.CIR-14-0053 PMC4306338

[270] R. K. Shrimali , Z. Yu , M. R. Theoret , et al., “Antiangiogenic Agents Can Increase Lymphocyte Infiltration Into Tumor and Enhance the Effectiveness of Adoptive Immunotherapy of Cancer,” Cancer Research 70, no. 15 (2010): 6171–6180. 20631075 10.1158/0008-5472.CAN-10-0153 PMC2912959

[271] Y. Chen , T. Ohara , Y. Hamada , et al., “HIF‐PH Inhibitors Induce Pseudohypoxia in T Cells and Suppress the Growth of Microsatellite Stable Colorectal Cancer by Enhancing Antitumor Immune Responses,” Cancer Immunology, Immunotherapy: CII 74, no. 7 (2025): 192. 40343532 10.1007/s00262-025-04067-3 PMC12064516

[272] Y. Wang , C. Wei , X. Zhao , et al., “A pH‐sensitive Peptide Amphiphilic‐based Drug Delivery System Inhibits Hepatocellular Carcinoma Growth by Suppressing Hepatic Stellate Cell Activation,” Materials Today Bio 32 (2025): 101821. 10.1016/j.mtbio.2025.101821 PMC12124664 40453820

[273] R. Cai , M. Wang , M. Pan , et al., “Inhibition of ARH2 by pH/ROS‐responsive Nanosystem for Improved Lung Adenocarcinoma Immunochemotherapy,” Bioactive Materials 53 (2025): 737–753. 40801020 10.1016/j.bioactmat.2025.07.042 PMC12341593

[274] H. Huang , N. Li , L. Zeng , et al., “Smart Biomimetic “Nano‐med‐fireman” Blocking Inflammation and Lactate Metabolism Crosstalk for Normalized Spatiotemporal Photo‐immunotherapy,” Bioactive Materials 51 (2025): 431–449. 40496624 10.1016/j.bioactmat.2025.05.012 PMC12149550

[275] D. Secci , S. Distinto , A. Onali , et al., “New Structural Features of Isatin Dihydrothiazole Hybrids for Selective Carbonic Anhydrase Inhibitors,” ACS Medicinal Chemistry Letters 15, no. 11 (2024): 1860–1865. 39563820 10.1021/acsmedchemlett.4c00280 PMC11571004

[276] W. H. Yang , Y. Qiu , O. Stamatatos , et al., “Enhancing the Efficacy of Glutamine Metabolism Inhibitors in Cancer Therapy,” Trends in Cancer 7, no. 8 (2021): 790–804. 34020912 10.1016/j.trecan.2021.04.003 PMC9064286

[277] Y. Fujiwara , S. Kato , M. K. Nesline , et al., “Indoleamine 2,3‐dioxygenase (IDO) Inhibitors and Cancer Immunotherapy,” Cancer Treatment Reviews 110 (2022): 102461. 36058143 10.1016/j.ctrv.2022.102461 PMC12187009

[278] C. H. Mochamat , M. Marinova , et al., “A Systematic Review on the Role of Vitamins, Minerals, Proteins, and Other Supplements for the Treatment of Cachexia in Cancer: A European Palliative Care Research Centre Cachexia Project,” Journal of Cachexia, Sarcopenia and Muscle 8, no. 1 (2017): 25–39. 27897391 10.1002/jcsm.12127 PMC5326814

[279] Q. Wang , J. Liu , M. Yang , et al., “Targeting AKR1B1 Inhibits Metabolic Reprogramming to Reverse Systemic Therapy Resistance in Hepatocellular Carcinoma,” Signal Transduction and Targeted Therapy 10, no. 1 (2025): 244. 40750772 10.1038/s41392-025-02321-9 PMC12317016

[280] H. Xu , Q. Liang , X. Xu , et al., “Afatinib Combined With anlotinib in the Treatment of Lung Adenocarcinoma Patient With Novel HER2 Mutation: A Case Report and Review of the Literature,” World Journal of Surgical Oncology 19, no. 1 (2021): 330. 34794435 10.1186/s12957-021-02444-7 PMC8600784

[281] S. C. Yu , S. S. Tong , Y. L. Chen , et al., “Efficacy and Safety of Cisplatin + Docetaxel + 5‐FU + Leucovorin + Methotrexate and Epirubicin Combination Chemotherapy for Advanced Esophageal Cancer,” PLoS ONE 20, no. 6 (2025): e0326056. 40570003 10.1371/journal.pone.0326056 PMC12200775

[282] C. Ye , M. Mi , S. Shi , et al., “ROS‐Responsive Hydrogel for Localized Delivery of Nampt and Stat3 Inhibitors Exhibits Synergistic Antitumor Effects in Colorectal Cancer through Ferroptosis Induction and Immune Microenvironment Remodeling,” Advanced Science (Weinheim, Baden‐Wurttemberg, Germany) 12, no. 33 (2025): e06599. 40492508 10.1002/advs.202506599 PMC12412517

[283] B. C. Özdemir , T. Pentcheva‐Hoang , J. L. Carstens , et al., “Depletion of Carcinoma‐associated Fibroblasts and Fibrosis Induces Immunosuppression and Accelerates Pancreas Cancer With Reduced Survival,” Cancer Cell 25, no. 6 (2014): 719–734. 24856586 10.1016/j.ccr.2014.04.005 PMC4180632

[284] P. S. Petrova , N. N. Viller , M. Wong , et al., “TTI‐621 (SIRPαFc): A CD47‐Blocking Innate Immune Checkpoint Inhibitor With Broad Antitumor Activity and Minimal Erythrocyte Binding,” Clinical Cancer Research: an Official Journal of the American Association for Cancer Research 23, no. 4 (2017): 1068–1079. 27856600 10.1158/1078-0432.CCR-16-1700

[285] A. Mantovani , “The Yin‐yang of Tumor‐associated Neutrophils,” Cancer Cell 16, no. 3 (2009): 173–174. 19732714 10.1016/j.ccr.2009.08.014

[286] C. Iliadi , L. Verset , C. Bouchart , et al., “The Current Understanding of the Immune Landscape Relative to Radiotherapy Across Tumor Types,” Frontiers in Immunology 14 (2023): 1148692. 37006319 10.3389/fimmu.2023.1148692 PMC10060828

[287] M. Jarosz‐Biej , R. Smolarczyk , T. Cichoń , et al., “Tumor Microenvironment as a “Game Changer” in Cancer Radiotherapy,” International Journal of Molecular Sciences 20, no. 13 (2019): 3212. 31261963 10.3390/ijms20133212 PMC6650939

[288] C. Zhang , Z. Liang , S. Ma , et al., “Radiotherapy and Cytokine Storm: Risk and Mechanism,” Frontiers in Oncology 11 (2021): 670464. 34094967 10.3389/fonc.2021.670464 PMC8173139

[289] B. C. Burnette , H. Liang , Y. Lee , et al., “The Efficacy of Radiotherapy Relies Upon Induction of Type i Interferon‐dependent Innate and Adaptive Immunity,” Cancer Research 71, no. 7 (2011): 2488–2496. 21300764 10.1158/0008-5472.CAN-10-2820 PMC3070872

[290] H. Liang , L. Deng , Y. Hou , et al., “Host STING‐dependent MDSC Mobilization Drives Extrinsic Radiation Resistance,” Nature Communications 8, no. 1 (2017): 1736. 10.1038/s41467-017-01566-5 PMC5701019 29170400

[291] L. B. Darragh , A. J. Oweida , and S. D. Karam , “Overcoming Resistance to Combination Radiation‐Immunotherapy: A Focus on Contributing Pathways within the Tumor Microenvironment,” Frontiers in Immunology 9 (2018): 3154. 30766539 10.3389/fimmu.2018.03154 PMC6366147

[292] M. Mondini , P. L. Loyher , P. Hamon , et al., “CCR2‐Dependent Recruitment of Tregs and Monocytes Following Radiotherapy Is Associated With TNFα‐Mediated Resistance,” Cancer Immunology Research 7, no. 3 (2019): 376–387. 30696630 10.1158/2326-6066.CIR-18-0633

[293] T. J. Curiel , G. Coukos , L. Zou , et al., “Specific Recruitment of Regulatory T Cells in Ovarian Carcinoma Fosters Immune Privilege and Predicts Reduced Survival,” Nature Medicine 10, no. 9 (2004): 942–949. 10.1038/nm1093 15322536

[294] S. W. Wang and Y. M. Sun , “The IL‐6/JAK/STAT3 Pathway: Potential Therapeutic Strategies in Treating Colorectal Cancer (Review),” International Journal of Oncology 44, no. 4 (2014): 1032–1040. 24430672 10.3892/ijo.2014.2259

[295] I. Larionova , N. Cherdyntseva , T. Liu , et al., “Interaction of Tumor‐associated Macrophages and Cancer Chemotherapy,” Oncoimmunology 8, no. 7 (2019): 1596004. 31143517 10.1080/2162402X.2019.1596004 PMC6527283

[296] D. G. DeNardo , D. J. Brennan , E. Rexhepaj , et al., “Leukocyte Complexity Predicts Breast Cancer Survival and Functionally Regulates Response to Chemotherapy,” Cancer Discovery 1, no. 1 (2011): 54–67. 22039576 10.1158/2159-8274.CD-10-0028 PMC3203524

[297] R. Hughes , B. Z. Qian , C. Rowan , et al., “Perivascular M2 Macrophages Stimulate Tumor Relapse After Chemotherapy,” Cancer Research 75, no. 17 (2015): 3479–3491. 26269531 10.1158/0008-5472.CAN-14-3587 PMC5024531

[298] E. S. Nakasone , H. A. Askautrud , T. Kees , et al., “Imaging Tumor‐stroma Interactions During Chemotherapy Reveals Contributions of the Microenvironment to Resistance,” Cancer Cell 21, no. 4 (2012): 488–503. 22516258 10.1016/j.ccr.2012.02.017 PMC3332002

[299] Y. Yang , S. Li , Y. Wang , et al., “Protein Tyrosine Kinase Inhibitor Resistance in Malignant Tumors: Molecular Mechanisms and Future Perspective,” Signal Transduction and Targeted Therapy 7, no. 1 (2022): 329. 36115852 10.1038/s41392-022-01168-8 PMC9482625

[300] S. Koyama , E. A. Akbay , Y. Y. Li , et al., “Adaptive Resistance to Therapeutic PD‐1 Blockade Is Associated With Upregulation of Alternative Immune Checkpoints,” Nature Communications 7 (2016): 10501. 10.1038/ncomms10501 PMC4757784 26883990

[301] J. Gao , J. F. Ward , C. A. Pettaway , et al., “VISTA Is an Inhibitory Immune Checkpoint That Is Increased After ipilimumab Therapy in Patients With Prostate Cancer,” Nature Medicine 23, no. 5 (2017): 551–555. 10.1038/nm.4308 PMC5466900 28346412

[302] F. S. Hodi , S. Lee , D. F. McDermott , et al., “Ipilimumab plus sargramostim vs ipilimumab Alone for Treatment of Metastatic Melanoma: A Randomized Clinical Trial,” Jama 312, no. 17 (2014): 1744–1753. 25369488 10.1001/jama.2014.13943 PMC4336189

[303] J. Pol , N. Bloy , A. Buqué , et al., “Trial Watch: Peptide‐based Anticancer Vaccines,” Oncoimmunology 4, no. 4 (2015): e974411. 26137405 10.4161/2162402X.2014.974411 PMC4485775

[304] H. L. Kaufman , D. W. Kim , G. DeRaffele , et al., “Local and Distant Immunity Induced by Intralesional Vaccination With an Oncolytic herpes Virus Encoding GM‐CSF in Patients With Stage IIIc and IV Melanoma,” Annals of Surgical Oncology 17, no. 3 (2010): 718–730. 19915919 10.1245/s10434-009-0809-6

[305] R. H. Andtbacka , H. L. Kaufman , F. Collichio , et al., “Talimogene Laherparepvec Improves Durable Response Rate in Patients with Advanced Melanoma,” Journal of Clinical Oncology: Official Journal of the American Society of Clinical Oncology 33, no. 25 (2015): 2780–2788. 26014293 10.1200/JCO.2014.58.3377

[306] J. Pol , G. Kroemer , and L. Galluzzi , “First Oncolytic Virus Approved for Melanoma Immunotherapy,” Oncoimmunology 5, no. 1 (2016): e1115641. 26942095 10.1080/2162402X.2015.1115641 PMC4760283

[307] W. Zou , J. D. Wolchok , and L. Chen , “PD‐L1 (B7‐H1) and PD‐1 Pathway Blockade for Cancer Therapy: Mechanisms, Response Biomarkers, and Combinations,” Science Translational Medicine 8, no. 328 (2016): 328rv324. 10.1126/scitranslmed.aad7118 PMC4859220 26936508

[308] C. X. Dominguez , S. Müller , S. Keerthivasan , et al., “Single‐Cell RNA Sequencing Reveals Stromal Evolution Into LRRC15(+) Myofibroblasts as a Determinant of Patient Response to Cancer Immunotherapy,” Cancer Discovery 10, no. 2 (2020): 232–253. 31699795 10.1158/2159-8290.CD-19-0644

[309] E. Elyada , M. Bolisetty , P. Laise , et al., “Cross‐Species Single‐Cell Analysis of Pancreatic Ductal Adenocarcinoma Reveals Antigen‐Presenting Cancer‐Associated Fibroblasts,” Cancer Discovery 9, no. 8 (2019): 1102–1123. 31197017 10.1158/2159-8290.CD-19-0094 PMC6727976

[310] P. Guo , L. Mao , Y. Chen , et al., “Multiplexed Spatial Mapping of Chromatin Features, Transcriptome and Proteins in Tissues,” Nature Methods 22, no. 3 (2025): 520–529. 39870864 10.1038/s41592-024-02576-0 PMC11906265

[311] A. Fiore , G. Yu , J. J. Northey , et al., “Live Imaging of the Extracellular Matrix With a Glycan‐binding Fluorophore,” Nature Methods 22, no. 5 (2025): 1070–1080. 39915692 10.1038/s41592-024-02590-2 PMC12074998

[312] B. H. Kann , D. F. Hicks , S. Payabvash , et al., “Multi‐Institutional Validation of Deep Learning for Pretreatment Identification of Extranodal Extension in Head and Neck Squamous Cell Carcinoma,” Journal of Clinical Oncology: Official Journal of the American Society of Clinical Oncology 38, no. 12 (2020): 1304–1311. 31815574 10.1200/JCO.19.02031

[313] S. Y. Yoo , H. E. Park , J. H. Kim , et al., “Whole‐Slide Image Analysis Reveals Quantitative Landscape of Tumor‐Immune Microenvironment in Colorectal Cancers,” Clinical Cancer Research: an Official Journal of the American Association for Cancer Research 26, no. 4 (2020): 870–881. 31757879 10.1158/1078-0432.CCR-19-1159

[314] V. H. Koelzer , K. Sirinukunwattana , J. Rittscher , et al., “Precision Immunoprofiling by Image Analysis and Artificial Intelligence,” Virchows Archiv: an International Journal of Pathology 474, no. 4 (2019): 511–522. 30470933 10.1007/s00428-018-2485-z PMC6447694

[315] M. Wiesweg , F. Mairinger , H. Reis , et al., “Machine Learning‐based Predictors for Immune Checkpoint Inhibitor Therapy of Non‐small‐cell Lung Cancer,” Annals of Oncology: Official Journal of the European Society for Medical Oncology 30, no. 4 (2019): 655–657. 30753264 10.1093/annonc/mdz049

[316] D. J. Irvine and E. L. Dane , “Enhancing Cancer Immunotherapy With Nanomedicine,” Nature Reviews Immunology 20, no. 5 (2020): 321–334. 10.1038/s41577-019-0269-6 PMC7536618 32005979

[317] K. Natrajan , M. Kaushal , B. George , et al., “FDA Approval Summary: Ciltacabtagene Autoleucel for Relapsed or Refractory Multiple Myeloma,” Clinical Cancer Research: an Official Journal of the American Association for Cancer Research 30, no. 14 (2024): 2865–2871. 38713595 10.1158/1078-0432.CCR-24-0378 PMC11249607

[318] K. Broos , Q. Lecocq , C. Xavier , et al., “Evaluating a Single Domain Antibody Targeting Human PD‐L1 as a Nuclear Imaging and Therapeutic Agent,” Cancers 11, no. 6 (2019): 872. 31234464 10.3390/cancers11060872 PMC6628009

[319] J. Heremans , R. Maximilian Awad , and J. Bridoux , “Sustained Release of a human PD‐L1 Single‐domain Antibody Using Peptide‐based Hydrogels,” European Journal of Pharmaceutics and Biopharmaceutics: Official Journal of Arbeitsgemeinschaft Fur Pharmazeutische Verfahrenstechnik eV 196 (2024): 114183. 10.1016/j.ejpb.2024.114183 38246566

[320] R. M. Awad , Q. Lecocq , K. Zeven , et al., “Formatting and Gene‐based Delivery of a human PD‐L1 Single Domain Antibody for Immune Checkpoint Blockade,” Molecular Therapy Methods & Clinical Development 22 (2021): 172–182. 34485603 10.1016/j.omtm.2021.05.017 PMC8397838

[321] Q. Li , J. Liu , Q. Zhang , et al., “The Anti‐PD‐L1/CTLA‐4 Bispecific Antibody KN046 in Combination With Nab‐paclitaxel in First‐line Treatment of Metastatic Triple‐negative Breast Cancer: A Multicenter Phase II Trial,” Nature Communications 15, no. 1 (2024): 1015. 10.1038/s41467-024-45160-y PMC10838317 38310192

[322] Y. Ma , J. Xue , Y. Zhao , et al., “Phase I Trial of KN046, a Novel Bispecific Antibody Targeting PD‐L1 and CTLA‐4 in Patients With Advanced Solid Tumors,” Journal for Immunotherapy of Cancer 11, no. 6 (2023): e006654. 37263673 10.1136/jitc-2022-006654 PMC10254625

[323] G. R. Khosravi , S. Mostafavi , S. Bastan , et al., “Immunologic Tumor Microenvironment Modulators for Turning Cold Tumors Hot,” Cancer Communications (London, England) 44, no. 5 (2024): 521–553. 38551889 10.1002/cac2.12539 PMC11110955

[324] X. Yu , Y. Long , B. Chen , et al., “PD‐L1/TLR7 Dual‐targeting Nanobody‐drug Conjugate Mediates Potent Tumor Regression via Elevating Tumor Immunogenicity in a Host‐expressed PD‐L1 Bias‐dependent Way,” Journal for Immunotherapy of Cancer 10, no. 10 (2022): e004590. 36253000 10.1136/jitc-2022-004590 PMC9577932

[325] L. Ma , X. Wang , Y. Wu , et al., “Controlled Release of Manganese and Magnesium Ions by Microsphere‐encapsulated Hydrogel Enhances Cancer Immunotherapy,” Journal of Controlled Release: Official Journal of the Controlled Release Society 372 (2024): 682–698. 38950681 10.1016/j.jconrel.2024.06.067

[326] V. Trivedi , C. Yang , K. Klippel , et al., “mRNA‐based Precision Targeting of Neoantigens and Tumor‐associated Antigens in Malignant Brain Tumors,” Genome Medicine 16, no. 1 (2024): 17. 38268001 10.1186/s13073-024-01281-z PMC10809449

[327] C. Cheng , W. Jiang , Y. Luo , et al., “NIR Activated Multimodal Therapeutics Based on Metal‐Phenolic Networks‐Functionalized Nanoplatform for Combating Against Multidrug Resistance and Metastasis,” Small (Weinheim an Der Bergstrasse, Germany) 19, no. 14 (2023): e2206174. 36651135 10.1002/smll.202206174

[328] W. Wang , F. Yang , L. Zhang , et al., “Targeting DNA Damage and Repair Machinery via Delivering WEE1 Inhibitor and Platinum (IV) Prodrugs to Stimulate STING Pathway for Maximizing Chemo‐Immunotherapy in Bladder Cancer,” Advanced Materials (Deerfield Beach, Fla) 36, no. 1 (2024): e2308762. 37849029 10.1002/adma.202308762

[329] X. Li , L. Luo , M. Jiang , et al., “Cocktail Strategy for ‘Cold’ tumors Therapy via Active Recruitment of CD8+ T Cells and Enhancing Their Function,” Journal of Controlled Release: Official Journal of the Controlled Release Society 334 (2021): 413–426. 33964366 10.1016/j.jconrel.2021.05.002

[330] Y. T. Liu and Z. J. Sun , “Turning Cold Tumors Into Hot Tumors by Improving T‐cell Infiltration,” Theranostics 11, no. 11 (2021): 5365–5386. 33859752 10.7150/thno.58390 PMC8039952

[331] X. Li , J. Wu , R. Xu , et al., “High Intensity Forced Ultrasound‐driven Ferroptosis as a Strategy for Anti‐tumor Immune Priming,” Acta Pharmaceutica Sinica B 15, no. 7 (2025): 3788–3804. 40698150 10.1016/j.apsb.2025.05.006 PMC12278624

[332] Q. Chen , T. Sun , and C. Jiang , “Recent Advancements in Nanomedicine for ‘Cold’ Tumor Immunotherapy,” Nano‐micro Letters 13, no. 1 (2021): 92. 34138315 10.1007/s40820-021-00622-6 PMC8006526

[333] P. Zheng , J. He , Y. Fu , et al., “Engineered Bacterial Biomimetic Vesicles Reprogram Tumor‐Associated Macrophages and Remodel Tumor Microenvironment to Promote Innate and Adaptive Antitumor Immune Responses,” ACS Nano 18, no. 9 (2024): 6863–6886. 38386537 10.1021/acsnano.3c06987

[334] R. Peng , Q. Huang , L. Wang , et al., “G‐Quadruplex RNA Based PROTAC Enables Targeted Degradation of RNA Binding Protein FMRP for Tumor Immunotherapy,” Angewandte Chemie (International Ed in English) 63, no. 47 (2024): e202402715. 39135270 10.1002/anie.202402715

[335] D. Kim , Y. Wu , G. Shim , et al., “Genome‐Editing‐Mediated Restructuring of Tumor Immune Microenvironment for Prevention of Metastasis,” ACS Nano 15, no. 11 (2021): 17635–17656. 34723493 10.1021/acsnano.1c05420

[336] J. Zhang , D. Huang , P. E. Saw , et al., “Turning Cold Tumors Hot: From Molecular Mechanisms to Clinical Applications,” Trends in Immunology 43, no. 7 (2022): 523–545. 35624021 10.1016/j.it.2022.04.010

[337] X. Meng , Z. Liu , L. Deng , et al., “Hydrogen Therapy Reverses Cancer‐Associated Fibroblasts Phenotypes and Remodels Stromal Microenvironment to Stimulate Systematic Anti‐Tumor Immunity,” Advanced Science (Weinheim, Baden‐Wurttemberg, Germany) 11, no. 28 (2024): e2401269. 38757665 10.1002/advs.202401269 PMC11267370

[338] Z. Zhang , Y. Zhou , S. Zhao , et al., “Nanomedicine‐Enabled/Augmented Cell Pyroptosis for Efficient Tumor Nanotherapy,” Advanced Science (Weinheim, Baden‐Wurttemberg, Germany) 9, no. 35 (2022): e2203583. 36266982 10.1002/advs.202203583 PMC9762308

[339] K. Gao , W. Xi , J. Ni , et al., “Genetically Modified Extracellular Vesicles Loaded With Activated Gasdermin D Potentially Inhibit Prostate‐specific Membrane Antigen‐positive Prostate Carcinoma Growth and Enhance Immunotherapy,” Biomaterials 315 (2025): 122894. 39461061 10.1016/j.biomaterials.2024.122894

[340] Z. Yang , D. Gao , X. Guo , et al., “Fighting Immune Cold and Reprogramming Immunosuppressive Tumor Microenvironment With Red Blood Cell Membrane‐Camouflaged Nanobullets,” ACS Nano 14, no. 12 (2020): 17442–17457. 33166111 10.1021/acsnano.0c07721

[341] H. Zhang , K. Feng , M. Han , et al., “Homologous Magnetic Targeted Immune Vesicles for Amplifying Immunotherapy via Ferroptosis Activation Augmented Photodynamic Therapy Against Glioblastoma,” Journal of Controlled Release: Official Journal of the Controlled Release Society 383 (2025): 113816. 40334815 10.1016/j.jconrel.2025.113816

[342] Z. Wang , T. Sha , J. Li , et al., “Turning Foes to Friends: Advanced “in Situ Nanovaccine” With Dual Immunoregulation for Enhanced Immunotherapy of Metastatic Triple‐negative Breast Cancer,” Bioactive Materials 39 (2024): 612–629. 38883315 10.1016/j.bioactmat.2024.04.023 PMC11179173

[343] W. Liu , X. Zhang , H. Xu , et al., “Microbial Community Heterogeneity within Colorectal Neoplasia and Its Correlation with Colorectal Carcinogenesis,” Gastroenterology 160, no. 7 (2021): 2395–2408. 33581124 10.1053/j.gastro.2021.02.020

[344] Y. Xie , F. Xie , X. Zhou , et al., “Microbiota in Tumors: From understanding to Application,” Advanced Science (Weinheim, Baden‐Wurttemberg, Germany) 9, no. 21 (2022): e2200470. 35603968 10.1002/advs.202200470 PMC9313476

[345] L. Yang , A. Li , Y. Wang , et al., “Intratumoral Microbiota: Roles in Cancer Initiation, Development and Therapeutic Efficacy,” Signal Transduction and Targeted Therapy 8, no. 1 (2023): 35. 36646684 10.1038/s41392-022-01304-4 PMC9842669

[346] Y. Ping , J. Shan , H. Qin , et al., “PD‐1 Signaling Limits Expression of Phospholipid Phosphatase 1 and Promotes Intratumoral CD8(+) T Cell Ferroptosis,” Immunity 57, no. 9 (2024): 2122–2139. e2129. 39208806 10.1016/j.immuni.2024.08.003

[347] Y. Chen , L. Yang , Y. Huang , et al., “Intratumoral Microbiota Predicts the Response to Neoadjuvant Chemoimmunotherapy in Triple‐negative Breast Cancer,” Journal for Immunotherapy of Cancer 13, no. 4 (2025): e010365. 40280564 10.1136/jitc-2024-010365 PMC12035477

[348] H. Wu , X. Leng , Q. Liu , et al., “Intratumoral Microbiota Composition Regulates Chemoimmunotherapy Response in Esophageal Squamous Cell Carcinoma,” Cancer Research 83, no. 18 (2023): 3131–3144. 37433041 10.1158/0008-5472.CAN-22-2593

[349] A. Elkrief , M. Montesion , S. Sivakumar , et al., “Intratumoral Escherichia Is Associated with Improved Survival to Single‐Agent Immune Checkpoint Inhibition in Patients with Advanced Non‐Small‐Cell Lung Cancer,” Journal of Clinical Oncology: Official Journal of the American Society of Clinical Oncology 42, no. 28 (2024): 3339–3349. 39038258 10.1200/JCO.23.01488 PMC11600405

